# Elevated HIV Viral Load is Associated with Higher Recombination Rate In Vivo

**DOI:** 10.1093/molbev/msad260

**Published:** 2024-01-10

**Authors:** Elena V Romero, Alison F Feder

**Affiliations:** Department of Genome Sciences, University of Washington, Seattle, WA, USA; Department of Genome Sciences, University of Washington, Seattle, WA, USA; Herbold Computational Biology Program, Fred Hutchinson Cancer Center, Seattle, WA, USA

**Keywords:** recombination, HIV, coinfection, virus, intrahost, density

## Abstract

HIV’s exceptionally high recombination rate drives its intrahost diversification, enabling immune escape and multidrug resistance within people living with HIV. While we know that HIV’s recombination rate varies by genomic position, we have little understanding of how recombination varies throughout infection or between individuals as a function of the rate of cellular coinfection. We hypothesize that denser intrahost populations may have higher rates of coinfection and therefore recombination. To test this hypothesis, we develop a new approach (recombination analysis via time series linkage decay or RATS-LD) to quantify recombination using autocorrelation of linkage between mutations across time points. We validate RATS-LD on simulated data under short read sequencing conditions and then apply it to longitudinal, high-throughput intrahost viral sequencing data, stratifying populations by viral load (a proxy for density). Among sampled viral populations with the lowest viral loads (<26,800 copies/mL), we estimate a recombination rate of $1.5\times 10^{-5}$ events/bp/generation (95% CI: $7\times 10^{-6}$ to $2.9\times 10^{-5}$), similar to existing estimates. However, among samples with the highest viral loads (>82,000 copies/mL), our median estimate is approximately 6 times higher. In addition to co-varying across individuals, we also find that recombination rate and viral load are associated within single individuals across different time points. Our findings suggest that rather than acting as a constant, uniform force, recombination can vary dynamically and drastically across intrahost viral populations and within them over time. More broadly, we hypothesize that this phenomenon may affect other facultatively asexual populations where spatial co-localization varies.

## Introduction

Recombination is a key evolutionary driver, permitting organisms to purge deleterious mutations and combine beneficial ones. These functions are critical in HIV (Onafuwa-Nuga and Telesnitsky 2009) where intrahost recombination promotes viral diversification, immune escape (Ritchie et al. 2014), multidrug resistance (Kellam and Larder 1995; Moutouh et al. 1996; Nora et al. 2007) and the maintenance of genomic integrity in spite of an exceptionally high viral mutation rate (King et al. 2008; Rawson et al. 2018). While average HIV recombination rates have been previously estimated on the order of $10^{-5}$/bp/generation or higher (Shriner et al. 2004; Neher and Leitner 2010; Batorsky et al. 2011), new investigations have revealed that this average fails to capture the full variation in recombination rate in multiple settings (Schlub et al. 2010; Smyth et al. 2014; Song et al. 2018). Because of recombination’s critical role in HIV’s evolutionary success, it is important to investigate the factors that underlie its variation.

**Fig. 1.**
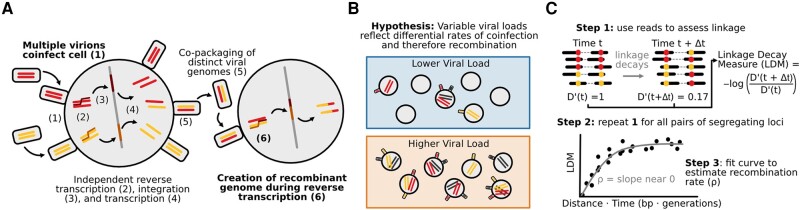
Drivers of HIV recombination rate. A) Cartoon schematic of HIV replication cycle leading to recombination between distinct viral genomes. Steps that determine the rate of recombination are shown in bold. B) HIV populations with higher viral loads (lower box) may experience higher rates of viral coinfection and therefore higher rates of recombination than HIV populations with lower viral loads (upper box). C) Linkage decay patterns enable recombination rate estimation. $D^{\prime }$ values quantify genetic linkage between pairs of SNPs at a given time point. Negative log ratios of $D^{\prime }$ values (here termed linkage decay measures or LDMs) across two sampled time points are positively associated with the product of the time between sample points and distance between SNPs. The slope of this relationship can be used to estimate the recombination rate, *ρ*. We call this method RATS-LD.

Two processes govern HIV’s recombination rate: cellular coinfection and reverse transcriptase template switching (Fig. 1A). When multiple viruses co-infect the same cell, the virions emerging from that cell can contain two distinct but copackaged viral genomes (as opposed to virions produced by singly infected cells which contain two identical genomes) (Jung et al. 2002; Chen et al. 2009). When those virions infect new cells, template switching between the two copackaged genomes can result in a recombinant genome inheriting alleles from both parent strands (Panganiban and Fiore 1988; Hu and Temin 1990; Hu and Hughes 2012). Increased rates of coinfection should result in more virions with distinct copackaged genomes, in which recombination can create new viral genotypes, while increased rates of template switching should result in more recombination events per genome pair.

Variation in the template switching rate has already been recognized as an important mediator of viral recombination both in vitro and in vivo. Template switching and, by extension, recombination can be suppressed by decreased sequence similarity between the two template strands (Balakrishnan et al. 2001; An and Telesnitsky 2002; Balakrishnan et al. 2003; Nikolaitchik et al. 2011). This effect is dose dependent (An and Telesnitsky 2002; Nikolaitchik et al. 2011) and extremely low levels of sequence similarity (<63%) near fully block template switching and recombination, leading to a breakdown of genomic integrity (Rawson et al. 2018). Recent sequence context aware work from human-derived HIV-1 sequences has identified particular genomic positions as hot and cold spots for template switching and recombination, which impact the ways in which viral populations diversify (Moumen et al. 2001; Schlub et al. 2010; Smyth et al. 2014; Song et al. 2018).

Despite its potential importance, we know little about how variation in coinfection rates impacts viral recombination in vivo. While studies of HIV in mouse models and cell cultures have demonstrated that increased coinfection is associated with an increase in recombinant viruses (Levy et al. 2004), it is unknown if this effect persists among naturally occurring HIV infections in humans. We expect coinfection to be mediated by the abundances and locations of both viruses and the CD4+ T-cells they infect (Jung et al. 2002; Chen et al. 2009). Therefore, we expect viral load—a proxy for overall viral abundance—to be related to the rate at which coinfection occurs, even if non-linearly. While previous studies have found a weak relationship between viral load and coinfection rate in chronic infection, it is unclear how generalizeable this relationship is and what effect, if any, it has on viral recombination rate (Josefsson et al. 2011). Since viral load and CD4+ T-cell count vary across individuals and within them over time, we hypothesize that viral intrahost demography modulates coinfection rates and may therefore play an important but previously uncharacterized role in determining the viral recombination rate in human hosts (Fig. 1B).

Existing statistical approaches to estimate recombination rate have made this effect challenging to detect in vivo. Current approaches to estimate the HIV recombination rate fall into two broad categories: identifying specific breakpoints in sequencing data (Siepel et al. 1995; Maydt and Lengauer 2006; Song et al. 2018; Martin et al. 2020), or employing overall patterns of linkage disequilibrium to infer intrahost recombination rates (breakpoint-free) (Shriner et al. 2004; Neher and Leitner 2010; Batorsky et al. 2011). Both rely on the analysis of single genome amplification and sequencing data (Salazar-Gonzalez et al. 2008), which substantially limits sampling depth and makes quantitative comparisons across different viral load groups challenging. Although Song et al. (2018) found no differences in recombination breakpoint counts at different viral loads, the number of breakpoints visible at any specific point in time is small which limits statistical power. While bulk-sequencing data enables deeper sampling than single genome sequencing, the relatively short reads commonly used in bulk-sequencing protocols limit linkage measurement except between nearby sites. However, HIV’s high recombination rate results in significant breakdown of genetic linkage even within the relatively short length of a typical bulk-sequencing paired-end read. Therefore, adapting breakpoint-free methods to work with bulk-sequencing data has the potential to more sensitively quantify the effects of recombination, even when stratifying datasets by viral load.

Here, we test for viral load-associated differences in recombination rate through developing a new approach called recombination analysis via time series linkage decay (RATS-LD) which quantifies recombination rates from longitudinal bulk-sequencing data. We validate that this method can successfully recover recombination rates in simulated short read paired-end data. To test for positive associations between HIV viral loads and recombination rate, we apply RATS-LD to longitudinal intrahost HIV bulk-sequencing data from individuals with varying viral loads sequenced with careful controls for polymerase chain reaction (PCR) recombination (Zanini et al. 2015, 2017). We find that while HIV populations with viral loads in the lowest third of dataset (<26,800 copies/mL) have recombination rates in line with previous estimates ($1.5\times 10^{-5}$/bp/generation), populations with viral loads in the upper third (>82,000 copies/mL) have a median recombination rate that is nearly 6-fold higher. Furthermore, within single individuals we observe patterns of viral load and effective recombination rate increasing concurrently. These findings demonstrate that recombination rate is not a static parameter, and intrahost conditions can mediate the strength of its effect both across and within individuals over time. Since recombination mechanisms generally require that two genomes physically find each other in space, such contact-network mediated effects are also likely to occur in populations beyond HIV.

## Results

### Measuring Intrahost Recombination Rates Using Short Read Data

We developed an approach to leverage short read sequencing data to quantify recombination rates in longitudinally sampled intrahost viral populations. To do so, we exploit the decay in genetic linkage over time between pairs of single nucleotide polymorphisms (SNPs) with elevated $D^{\prime }$ values (Lewontin 1988). $D^{\prime }$ quantifies the difference between the expected haplotype frequencies of two SNPs at linkage equilibrium and the observed haplotype frequencies, equaling 0 when the frequencies match and 1 when they diverge maximally. Many factors govern the equilibrium value of $D^{\prime }$ in a population, including recombination, mutation, and genetic drift and draft (Kimura and Ohta 1971; Charlesworth and Charlesworth 2010). Linkage-increasing forces can elevate $D^{\prime }$ between pairs of loci at specific points in time, but recombination will disassociate these linked mutations back towards linkage equilibrium. This disassociation will be faster if the SNPs are separated by greater physical distances (*d*, measured in nucleotides), as the increased distance provides more opportunities for recombination events between the SNPs. Specifically, for a pair of segregating SNPs with elevated linkage, the linkage decay after a period of time Δt with a recombination rate *ρ* and no countervailing forces is given as follows (Hartl et al. 1997):


(1) $$D^{\prime }(t+\Delta t)=D^{\prime }(t)e^{-\rho d\Delta t}.$$

At long time intervals, this approximation breaks down, failing to account for increases in linkage caused by mutation, selection and drift (Kimura and Ohta 1971; Charlesworth and Charlesworth 2010). However, over shorter timescales, equation (1) captures the temporal decay of $D^{\prime }$ between two initially highly linked SNPs, and potentially permits estimation of *ρ* as follows:


(2) $$\text{Linkage decay measure (LDM) }=-\log\;\left(\frac{D^{\prime }(t+\Delta t)}{D^{\prime }(t)}\right)=\rho d\Delta t.$$

We compute the negative log ratio on the left-hand side of equation (2) (which we call the linkage decay measure or LDM) over many pairs of SNPs between multiple time points. We can then use these LDMs to derive an empirical relationship between the linkage decay and the time and distance separating each LDM’s corresponding pair of SNPs (Fig. 1C). The slope of this empirical relationship is the estimated recombination rate $\hat{\rho }$. This approach, which we call recombination analysis via time series linkage decay (RATS-LD), is a generalization of existing methods for recombination rate estimation (Neher and Leitner 2010) which permits application in short read sequencing data.

### Validating RATS-LD on Simulated Data under Neutrality

**Fig. 2.**
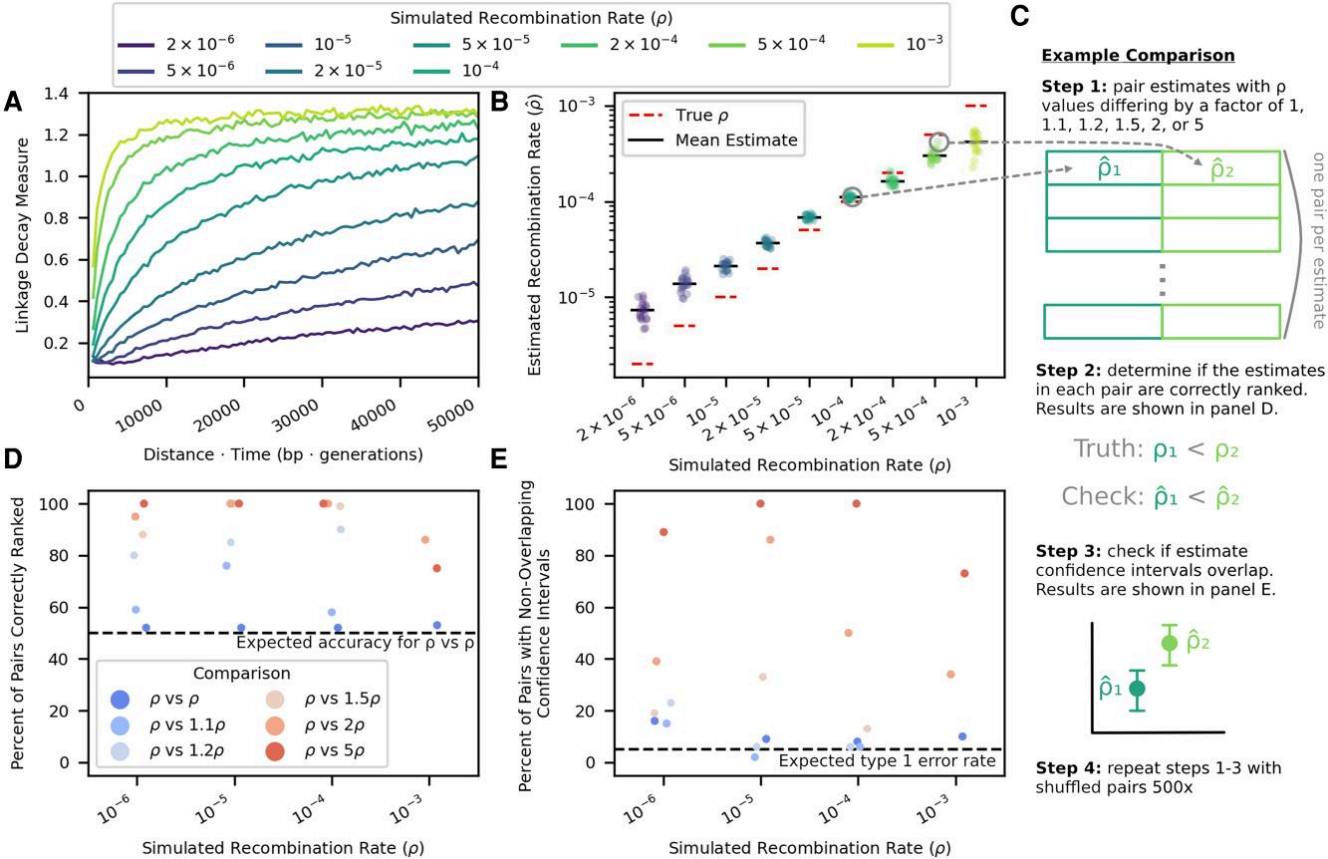
RATS-LD recaptures true recombination rates in simulated data and accurately distinguishes between different rates. A) Linkage decay curves calculated from simulated data exhibit saturation patterns with respect to dΔt that depend on the underlying recombination rate, *ρ* (moving average displayed, window size = 500 bp × generations). B) The slopes of these curves at dΔt=0 serve as RATS-LD’s estimates and recapture the known *ρ* value in simulated data. Each point represents the median of a bootstrapped distribution of 1,000 estimates. C) We performed a discrimination analysis to confirm that RATS-LD can distinguish different recombination rates. We simulated data with set recombination rates and estimated $\hat{\rho }$ using RATS-LD. We then randomly paired simulations with recombination rates that differed by a factor of 1, 1.1, 1.2, 1.5, 2, or 5 to assess how often RATS-LD correctly distinguished underlying recombination rates via simple rank order D) and non-overlapping bootstrapped 95% confidence intervals E).

RATS-LD relies on capturing differential rates of linkage decay at different distances within a given time interval to estimate the recombination rate. Because it relies on an approximation [equation (1)] that diverges from the expected linkage behavior at long time-scaled distances, we first sought to assess if RATS-LD could accurately estimate *ρ* given the sampling time intervals (150 to 300 generations), detectable distances between SNPs (1 to 400 bp), and evolutionary dynamics observed in longitudinal viral sequencing studies (Shankarappa et al. 1999; Zanini et al. 2015). We simulated intrahost evolution (initially neutrally) with a range of different recombination rates and tested RATS-LD’s ability to recover these rates. We centered our simulated recombination rates around existing HIV intrahost estimated recombination rates [ $\rho =2\times 10^{-6}$ to $10^{-3}$ (Shriner et al. 2004; Neher and Leitner 2010; Batorsky et al. 2011), see Materials and methods for full simulation and sampling details]. In these simulations, we observed that within our sampling times, the relationship between the linkage decay measure for a pair of initially highly linked SNPs ($D^{\prime }(t)>0.2$) and the time-scaled physical distance (dΔt) initially increased, reflecting the breakdown of linkage at greater distances. This suggests that over the examined sampling time intervals, nearby sites contain information about recombination driven linkage breakdown. Following this initial increase, the linkage relationship asymptotically approached a given LDM at large dΔt values, indicating a return to background linkage levels. The rate at which this asymptote is approached depends strongly on the recombination rate (Fig. 2A, supplementary Fig. S1, Supplementary Material online).

To account for this asymptotic return to equilibrium, we modified our approach for estimating *ρ* by fitting a function of the form


(3) $$\hat{\text{LDM}}(d\Delta t)=c_{0}+c_{1}(1-e^{-c_{2}d\Delta t}).$$

The behavior of $\hat{\text{LDM}}$ near dΔt=0 represents the portion of the curve shaped most strongly by recombination-driven linkage breakdown, as opposed to population equilibrium linkage levels which are governed by several forces in addition to recombination. Therefore, in analog to using the slope of equation (1), we use the derivative of $\hat{\text{LDM}}$ with respect to dΔt at 0 as our estimator of *ρ*, and find that it recaptures the true recombination rate in simulated data from neutrally evolving populations (Fig. 2B). We noted that estimation at high or low values of *ρ* was biased downward or upward, respectively.

This minor bias emerged due to challenges fitting linkage decay curves with limited paired-end data in scenarios where the degree of linkage breakdown on the sampling timescale returned too quickly or too slowly to equilibrium to accurately measure the rate. To demonstrate that the slope of the linkage decay measure at 0 does indeed recapture the true recombination rate, we fit linear relationships between dΔt and the linkage decay measure for subsets of the data near dΔt=0 (i.e. precurve saturation). We found that these linear fits accurately recaptured the true recombination rate when only presaturation data was included in the fit (supplementary Figs. S2 and S3, Supplementary Material online). However, the linkage decay curves saturate at different dΔt for different values of *ρ*, rendering these linear fits unsuitable when the true recombination rate is unknown (Fig. 2A and supplementary Fig. S3, Supplementary Material online). In contrast, the curve-fitting approach can be applied without a known dΔt saturation point. For low recombination rates, the linkage decay curves do not saturate in the time-scaled distances available from the relatively short simulated reads (all pairs of SNPs less than 700 bp apart) (Fig. 2A). As a result, RATS-LD underestimates the asymptote of equation (3), which results in a slight overestimation of *ρ* (Fig. 2B). Among very high recombination rates, little data is available before curve saturation, resulting in challenges fitting the behavior of equation (3) near dΔt=0 and underestimation of *ρ*. For full details on how these fits were performed, see Materials and methods. See Discussion for a description of best practices for assessing whether or not a RATS-LD fit should be considered reliable.

Despite the challenges of quantifying the recombination rate at more extreme *ρ* values, we found that RATS-LD could readily discriminate between differences in recombination rates in simulated data, particularly in the range of previously estimated HIV recombination rates ($10^{-5}$ to $10^{-4}$). We assessed this in two ways: first, we compared RATS-LD estimates ($\hat{\rho }$ values) across pairs of simulations and checked if the simulation with the higher true *ρ* also had the higher estimated $\hat{\rho }$ (Fig. 2C and D). In the range of $10^{-5}$ to $10^{-4}$, we found that pairs of simulations with a 1.5-fold or greater difference in *ρ* were nearly always ordered correctly using RATS-LD. Outside of this range (at values of $10^{-6}$ and $10^{-3}$), recombination rate differences must be larger for RATS-LD to successfully order them due to lower method resolution in these regimes. Second, for each pair of simulations, we checked whether their bootstrapped $\hat{\rho }$ confidence intervals overlapped (Fig. 2E). We found that we had a low probability of type I errors (i.e. assessing differences in recombination rates when no such differences existed). At recombination rates of $10^{-3}$ and $10^{-6}$, RATS-LD could often discriminate simulations with true *ρ* values separated by a factor of 2 or 5. In the range of $10^{-5}$ to $10^{-4}$, RATS-LD could nearly always discriminate simulations with true *ρ* values a factor of 5 apart and could often discriminate *ρ* values a factor of 2 apart, but was not well powered to detect more subtle differences in recombination rate.

### Validating RATS-LD under Settings That Better Recapitulate Intrahost HIV

**Fig. 3.**
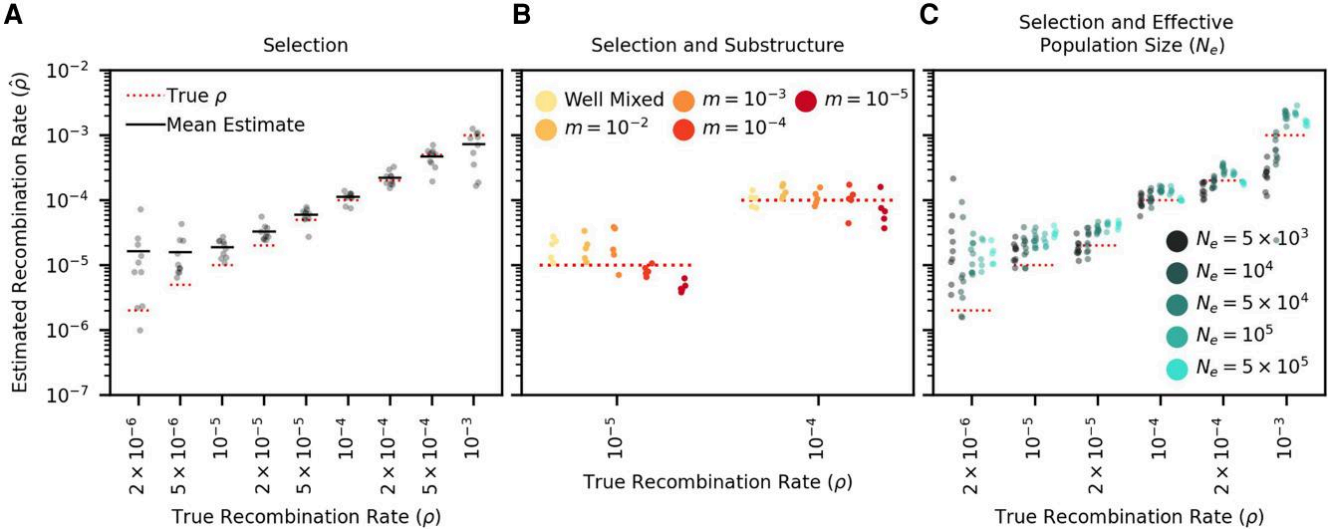
RATS-LD can estimate recombination rates in conditions that mimic HIV intrahost evolution. A) Recombination rate estimates from simulations performed with HIV-like selective conditions recapture known *ρ* values. B) Recombination rate estimates from simulated subdivided populations interconnected by bidirectional migration. Each generation, a proportion *m* of each subpopulation emigrates from the other subpopulation ($m=10^{-2},10^{-3},10^{-4},10^{-5}$). C) Recombination rate estimates in datasets simulated under a range of effective population sizes ($N_{e}=5\times 10^{3}$ to $5\times 10^{5}$). We plot ten estimation groups for $N_{e}$ values $<10^{5}$ and five estimation groups for $N_{e}$ values $\geq 10^{5}$. In all three panels, each point represents the median estimate over 1,000 bootstraps. For full details on all of the specific simulation scenarios, see Materials and methods.

While RATS-LD can discriminate between recombination rates in neutrally simulated populations, intrahost HIV is under strong selective pressures which will affect the equilibrium linkage levels and could consequently affect the rate of linkage decay under a given *ρ*. We therefore extended our simulations to mimic these pressures, adding both positive and negative selection (*s* ranges from −0.1 to 0.05, see Materials and methods for full distribution of fitness effects). We chose these values based on estimates from the literature (Zanini et al. 2017), and confirmed that they match in vivo intrahost divergence and diversity over the first 8 years of infection in a longitudinally sampled cohort (Zanini et al. 2015, supplementary Fig. S4, Supplementary Material online). We found that selection simulations produced noisier linkage decay curves than neutral simulations (supplementary Fig. S5, Supplementary Material online), but RATS-LD was still able to recapture underlying recombination rates (Fig. 3A). Similar to the neutrally simulated data, RATS-LD successfully estimated recombination rates in the $10^{-5}$ to $10^{-4}$ range but lost resolution at more extreme values.

Another complication of intrahost HIV evolution is population structure between different viral subpopulations in the body, which can maintain longer range linkage and potentially affect estimation. To examine the impact of population structure, we simulated two subpopulations of HIV interconnected by migration at rate *m*. We chose a range of *m* values to broadly encompass extremely low migration rates considered in the literature (Lythgoe et al. 2016) up to well-mixed populations.

We found that RATS-LD estimation was minimally impacted by migration rates greater than $10^{-4}$, although extremely low migration rates ($m=10^{-5}$) did depress estimates slightly (Fig. 3B). To understand if this underestimation was likely to impact application of RATS-LD to in vivo data with an unknown amount of population substructure, we compared the average linkage in the simulated structured data to the in vivo data from Zanini et al. (2015) used above as a reference for genetic divergence and diversity. Notably, this dataset contained two superinfected individuals who displayed long range linkage suggestive of population structure. Even in these individuals, D’ decayed faster over distance than the simulated $m=10^{-5}$ populations (supplementary Fig. S6, Supplementary Material online). This suggests that these participants did not possess migration rates low enough to substantially alter RATS-LD’s performance.

Finally, we considered whether variation in effective population size ($N_{e}$) would change linkage patterns strongly enough to alter RATS-LD’s accuracy. We therefore ran selection simulations for four additional values of $N_{e}$ spanning the range of $5\times 10^{3}$ to $5\times 10^{5}$, which captures variation in estimated short-term $N_{e}$ of intrahost HIV in the literature (Achaz et al. 2004; Feder et al. 2017).

Generally, we found that while increases in the effective population size resulted in moderately faster linkage breakdown, the effect on our estimation was relatively small, especially in the range of recombination rates surrounding existing HIV estimates (Fig. 3C). For example, the average estimated recombination rates for $N_{e}$ values a factor of 100 apart only differed by a factor of ≈2.5 [$N_{e}=5\times 10^{3}$ vs. $5\times 10^{5}$ at $\rho =2\times 10^{-5}$/bp/generation, a rate near the prevailing estimate (Neher and Leitner 2010)]. We do note that at very high values of $N_{e}$ and *ρ*, very rapid linkage decay renders the sampling scheme (time points 150 to 300 generations apart) too coarse to collect a baseline measurement of linkage decay near d=0. As a result, our fits for the linkage decay curve’s *y*-intercept are pushed upwards which biases our estimates for the recombination rate downwards for these simulations. We note more broadly that these values are unlikely to be relevant for the intrahost data, as simulations with very high $N_{e}$ fail to match key metrics of the Zanini et al. (2015) in vivo dataset in terms of population diversity and divergence (supplementary Fig. S4, Supplementary Material online). These findings indicate that while RATS-LD estimates are not agnostic to differences in effective population size, variation in effective population size is unlikely to be the sole driver of large differences in RATS-LD estimates, especially in conditions similar to intrahost HIV dynamics.

### Applying RATS-LD to In Vivo HIV Data

Thus established, we sought to use RATS-LD to quantify how recombination rate varied within and across intrahost HIV populations with different viral loads. We analyzed viral sequences from a cohort of 10 people living with HIV, who were sampled longitudinally over 6 to 12 years and were not on antiretroviral therapy (ART) (Zanini et al. 2015), using a workflow illustrated in supplementary Fig. S7, Supplementary Material online. Although the sampling scheme spans a much longer time period, nearly all (≈96%) of the time point pairs included in the analysis were separated by fewer than 300 generations since very few alleles segregated for longer. We chose this dataset because the authors recorded contemporaneous viral load measurements and sequenced these data via a workflow that minimized PCR recombination (see Discussion) (Zanini et al. 2017). Therefore, we expect the detected signals of recombination in these data to be driven by in vivo processes, rather than amplification artifacts. This dataset also had a sampling depth (median ≈800) far deeper than was necessary for accurate RATS-LD estimation (supplementary Fig. S8A, Supplementary Material online). RATS-LD estimated the average recombination rate across this dataset to be $3.6\times 10^{-5}$ events/bp/generation (95% CI $2.8\times 10^{-5}$ to $4.7\times 10^{-5}$) which is slightly higher than previous estimates (Shriner et al. 2004; Neher and Leitner 2010; Batorsky et al. 2011), but consistent under RATS-LD’s slight upward bias in this range.

**Fig. 4.**
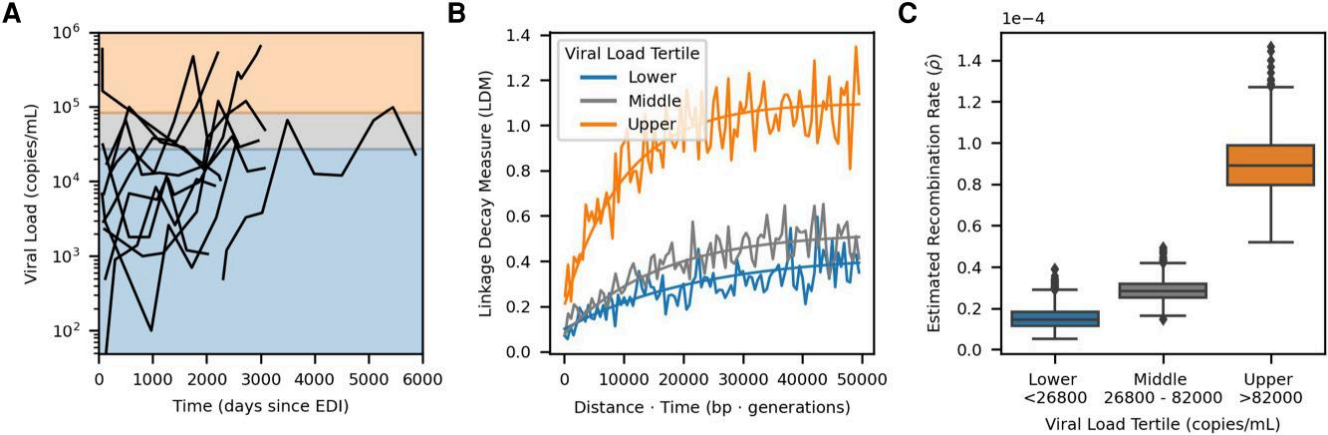
Elevated recombination rates are associated with high viral load in vivo. A) Viral load time courses for each participant are plotted. The background shading and horizontal demarcations indicate the viral load tertiles: <26,800 (lower/blue), 26,800 to 82,000 (middle/gray), and >82,000 (upper/orange) which divide the linkage decay measures (LDMs) into three approximately equal sized groups (≍ 20k LDMs per group). While viral loads at single time points are shown here, pairs of time points were used to form the groups for estimation and were sorted based on their average viral load (see supplementary Figs. S9 and S10, Supplementary Material online). B) Curve fits used by RATS-LD for estimation. Curve fits representing the median estimates of the bootstrap distribution (1,000 bootstraps) for each group are overlaid on the binned average of the LDMs from the in vivo data (moving average used for display purposes only, window size = 500 bp × generations). C) Recombination rate estimates $\hat{\rho }$ with 1,000 bootstraps are shown separately for the three tertiles. Each box represents the quartiles of the $\hat{\rho }$ distribution and the median is indicated by the central line.

Within this dataset, viral loads varied within and between individuals (supplementary Fig. S9, Supplementary Material online), as has been observed in other cohorts (Shankarappa et al. 1999; Robb et al. 2016). For each pair of time points within an individual, we computed the mean viral load (supplementary Fig. S10A, Supplementary Material online). Time points did not need to be consecutive to be compared, but we excluded any time point pairs where an intervening viral load measurement did not fall between the measurements at the two compared time points (see Materials and methods). Next, we associated each linkage decay measure to the mean viral load between the two time points (*t* and t+Δt). The distribution of LDMs and their associated average viral load measures is shown in supplementary Fig. S10B, Supplementary Material online. Then, we split the data based on the viral load tertiles which divided the data into three groups with approximately equal numbers of LDMs. This provided a sufficient number of loci for estimation (supplementary Table S1; Fig. S11B and C, Supplementary Material online). In Fig. 4A, we show the viral load time courses for each participant with shading corresponding to the viral load tertiles.

We used RATS-LD to compute $\hat{\rho }$ separately for each of the viral load groups (Fig. 4B–C). We found that $\hat{\rho }$ among time point pairs below the first tertile (<26,800 copies/mL) was $1.5\times 10^{-5}$ recombination events/bp/generation (bootstrapped confidence interval $7\times 10^{-6}$ to $2.9\times 10^{-5}$), a rate nearly identical to existing recombination rate estimates for HIV (Neher and Leitner 2010). However, among pairs of time points with the highest average viral loads (>82,000 copies/mL), the estimated median recombination rate was nearly 6 times higher: $8.9\times 10^{-5}$ recombination events/bp/generation (bootstrapped confidence interval $6.5\times 10^{-5}$ to $1.2\times 10^{-4}$). The middle tertile fell between the other two, although substantially closer to the lower tertile (bootstrapped confidence interval $2\times 10^{-5}$ to $4\times 10^{-5}$).

We performed several analyses to ensure the robustness of this result. First, we demonstrated that the observed differences were not solely driven by the data of any individual participant by conducting a leave-one-out analysis (supplementary Fig. S12, Supplementary Material online). Second, we performed a variation of this analysis where we left out data from two superinfected individuals (Participants 4 and 7). These individuals exhibited long range linkage patterns that are potentially consistent with population substructure maintained by limited migration, although the degree of linkage does not suggest that we are outside of the range of population structure where RATS-LD can estimate accurately (supplementary Fig. S6, Supplementary Material online, Fig. 3B). Regardless, the results from this analysis were also consistent with the findings displayed in Fig. 4 and showed a significant difference between the lower and upper viral load tertile estimates (supplementary Fig. S12, Supplementary Material online). Third, we attempted to verify this result with a biological replicate from a different dataset (Caskey et al. 2017). Caskey et al. (2017) sequenced HIV from longitudinally sampled viral populations rebounding after an infusion of broadly neutralizing antibodies. As a result of this context, populations possessed relatively low viral loads (12,766±9,859 copies/mL, mean ± SD). The amount of data available and low sampling depth permitted only a single, noisier estimate as opposed to the viral load-segregated analysis of Zanini et al. (2015). However, the estimate from Caskey et al. (2017) was consistent with the lowest tertile data from Zanini et al. (2015), in line with their comparable viral loads (supplementary Fig. S8B, Supplementary Material online).

Fourth, to further quantify the relationship between viral load and estimated recombination rate, we also conducted an analysis in which we grouped LDMs depending on their average viral load meeting a given threshold (supplementary Figs. S13 and S14, Supplementary Material online). This permitted us to further determine if viral load has a dose-dependent effect on recombination rate. The estimated recombination rate became more extreme at higher viral load thresholds, and time points with a mean viral load above 200,000 copies/mL showed an estimated $\hat{\rho }$ nearly an order of magnitude higher than prevailing estimates ($1.1\times 10^{-4}$, confidence interval $7.5\times 10^{-5}$ to $1.6\times 10^{-4}$), although small sample sizes led to substantial variation in curve fitting and correspondingly large bootstrapped confidence intervals at more extreme thresholds. Taken together, these results suggest that HIV populations at high viral loads may be recombining significantly more often than previous estimates.

**Fig. 5.**
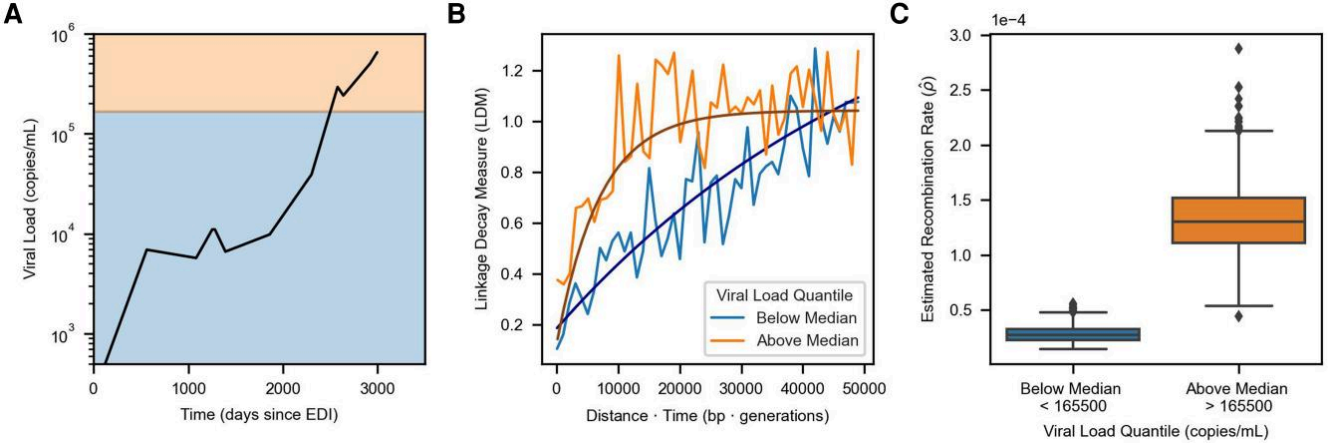
High viral load is also associated with elevated recombination rate in Participant 1. A) Viral load trajectory over the number of days since estimated date of infection (EDI) for Participant 1. The background shading indicates the two groups (above the horizontal median line in orange and below the line in blue) that pairs of time points were divided into based on their average viral load. Each group contains approximately 6,000 LDMs. B) Curve fits representing the median estimates from each group’s corresponding bootstrap distribution (1,000 bootstraps) are overlaid on the binned average of the LDMs in the in vivo data for Participant 1 (moving average used for display purposes only, window size = 1,000 bp × generations). C) Recombination rate estimates for times with average viral load measurements above or below the median using only data from Participant 1. Each box indicates the interquartile range of the bootstrapped distribution of $\hat{\rho }$ for the given viral load group (1,000 Bootstraps). The median estimate is indicated via the central line. Identical analyses for Participants 3, 4, and 7 are shown in supplementary Fig. S15, Supplementary Material online.

The analyses above suggest that higher viral loads are correlated with faster recombination across individuals, but viral loads can also vary significantly within individuals over time. We therefore set out to examine if RATS-LD could detect differences in recombination rates within single individuals. Only one individual (Participant 1) in our dataset had enough data above the threshold at which we saw substantial differences in $\hat{\rho }$ (>50k copies/mL, supplementary Fig. S13, Supplementary Material online) to perform recombination rate estimation according to our power analysis. Participant 1 possessed both extensive genetic variation (≈12,000 LDMs) and a broad range of viral load measurements ($<10^{3}$ to $>10^{6}$ copies/mL). We divided the LDMs for Participant 1 into two equally sized groups based on their viral loads and found a significant difference in recombination rates between time points with viral loads above and below the median of 165,500 copies/mL (${\hat{\rho }}_{<165\text{k}}=2.8\times 10^{-5}$, confidence interval = $1.7\times 10^{-5}$ to $4.5\times 10^{-5}$ and ${\hat{\rho }}_{>165\text{k}}=1.3\times 10^{-4}$, confidence interval = $7.4\times 10^{-5}$ to $2\times 10^{-4}$) as shown in Fig. 5. Although we did not expect to be well powered to detect recombination rate differences in any other participants, we repeated this analysis with Participants 3, 4, and 7, who had the next largest numbers of LDMs available for estimation (supplementary Tables S1 and S2, Supplementary Material online). Consistent with the results from Participant 1, time points with viral loads below the median had lower estimated recombination rates than those with viral loads above the median (supplementary Fig. S15, Supplementary Material online), although the bootstrapped confidence intervals between the high and low viral load groups overlapped in all three participants. These results are not unexpected as Participants 3, 4, and 7 had much narrower ranges of viral loads than Participant 1 and RATS-LD is not well powered to detect small differences in recombination rate (Fig. 2) especially at smaller sample sizes (supplementary Fig. S11, Supplementary Material online). Nevertheless, the analyses within individuals collectively suggest that as a population’s viral load varies over time, evolutionarily important parameters such as the recombination rate can vary concurrently.

## Discussion

HIV evolution within a host is a complex process driven by shifting selection pressures and variation in mutational availability as viral loads expand and contract over orders of magnitude. Here, we show that the recombination rate of HIV in vivo can also change dynamically over the course of infection in concert with changes in the viral load and effective population density. We introduce an approach, RATS-LD, which estimates significantly elevated recombination rates in viral populations with the highest viral loads. In addition to quantifying differences across viral populations, we also detect a nearly order of magnitude difference in recombination rates within a single viral population as its viral load increases over time. Although HIV recombination has long been appreciated to be a common event, these results suggest that in certain intrahost contexts, rates of recombination can be even more extreme than previously recognized (Shriner et al. 2004; Neher and Leitner 2010; Batorsky et al. 2011).

Our simulations with varying effective population sizes ($N_{e}$) suggest that these higher recombination rates among higher viral load populations are not simply caused by positive correlations between $N_{e}$ and viral load. We found that changing HIV $N_{e}$ within a plausible range of values resulted in much smaller differences in estimated recombination rates than the ones we observed between high and low viral load groups in vivo (Fig. 3B). That being said, an increase in $N_{e}$ in populations at high viral loads may be one contributing factor to the increased estimate of *ρ*.

We find that recombination rate increases most drastically in populations with the highest viral loads, consistent with a study that found only modest variation in in vivo CD4+ T-cell coinfection rates among viral populations with viral loads less than $10^{5}$ copies/mL, but elevated coinfection rates in a population with viral load above $10^{6}$ copies/mL (Josefsson et al. 2011). Our study not only suggests that these results hold in an independent cohort, but further links variable rates of coinfection to quantitatively different viral recombination rates in vivo. While this effect has previously been demonstrated in cell culture where viral density can be experimentally manipulated (Levy et al. 2004), this represents the first evidence that intrahost viral density varies extensively enough to cause substantial changes to the recombination rate in vivo in human hosts.

This previously unappreciated recombination rate variation likely impacts both intrahost evolutionary dynamics and also interpretations of intrahost viral data. While most of the sequencing data we investigated were not from acute infection, the initial HIV expansion brings the viral load its most extreme values (Selik and Linley 2018). If elevated recombination rates broadly accompany high viral loads, recombination may drive the creation of novel viral haplotypes to a previously unrecognized extent, particularly in populations with multiple founders. The importance of this effect may be counteracted by relatively low population diversity in the early stages of infection (Zanini et al. 2015). This interaction merits future investigation. Recent work has also suggested that elevated recombination rates could mask signatures of spatial compartmentalization in intrahost HIV populations (Sarkar et al. 2023). In contrast, low viral loads in individuals on incompletely suppressive ART may lead to correspondingly low recombination rates, mitigating recombination of distinct drug resistance mutations on different genetic backgrounds as a path to multidrug resistance (speculated in Feder et al. 2021). Lower rates of recombination in lower viral load populations may also make phylogenetic approaches that rely on treelike evolutionary divergence more tractable to apply without requiring ancestral recombination graphs (Castro et al. 2023). While we did not have access to samples with extremely low viral loads near the limit of detection, future investigations could determine if previously reported average recombination rates ($\approx 1.4\times 10^{-5}$) can explain low viral load patterns of linkage decay.

Viral load is the most accessible proxy for measuring population density, but it does not capture the complex dynamics of HIV metapopulations which can span distinct anatomical locations and may operate differently across individuals (Lythgoe et al. 2016). Since most T-cells in a person living with HIV are uninfected, local population density of infected cells is likely critically important for determining *ρ*. Future investigation into this hypothesis would be best served by localized measurements of coinfection and gene exchange in lymphatic tissues at different viral densities. These samples are invasive to collect, especially longitudinally, so approaches that consider aggregate measurements at the population level are valuable proofs of principle. The fact that positive associations with recombination rate can be observed even for high level measurements like viral load underscores density’s importance as a mediator of in vivo recombination.

Like other in vivo methods, RATS-LD relies on naturally segregating sites as genetic markers to track the breakdown of linkage. While the rate of this breakdown is not affected by population diversity, our ability to accurately quantify its average does depend on the number of segregating sites we can identify. Therefore, having more or fewer mutations stands only to impact the confidence of RATS-LD rather than bias the estimated recombination rates themselves. Less diverse populations require more pooling to have the requisite number of pairs of data points. This limits our ability to powerfully estimate recombination rates in smaller, less diverse populations.

Importantly, we cannot detect recombination events between identical sequences. If genetically identical viral bursts are largely confined to a given spatial location, rates of coinfection and recombination may be high but undetectable to this method. Similar effects could be driven by cell-to-cell transmission (Kreger et al. 2021). While important for understanding the viral life history and phenomena like complementation (Gelderblom et al. 2008) and replication fidelity (King et al. 2008; Rawson et al. 2018), these undetectable recombination events are unlikely to contribute meaningfully to the population’s evolution because they do not generate new viral haplotypes. As such, our estimated $\hat{\rho }$ values reflect the effective rates of recombination and mixing discounting population structure.

Although we analyzed data with careful controls for PCR recombination, we cannot completely rule it out as a complicating factor (Salazar-Gonzalez et al. 2008). In order to minimize PCR recombination, Zanini et al. (2015, 2017) only used one round of PCR (rather than a more standard nested approach) and optimized polymerases and extension times, among other measures. They validated this approach by comparing linkage decay in a 50/50 mix of two laboratory strains. While no linkage decay was observed in this mixture, the reduced sequence similarity between the two strains may also have discouraged recombination (Balakrishnan et al. 2001, 2003). As further validation, they analyzed a natural control of an intrahost population founded by two distinct viruses. Despite the distinct founder viruses having high sequence similarity, nearly complete linkage was preserved after a single round of PCR amplification. Additionally, these control samples were from relatively high viral load time points ($\approx 10^{5}$ copies/mL) indicating that PCR recombination is extremely low even in the high viral load samples we analyzed. The agreement between the estimates from the Caskey et al. (2017) data (produced via single genome sequencing) and the low viral load tertile of the Zanini et al. data also supports this point. While we cannot definitively say that PCR recombination did not in any way affect our results, these controls provide reassurance that rates of in vitro PCR recombination were extremely low.

It is important to note several additional caveats with our analysis: (i) Correlation does not imply causation. Although we report an association between recombination rate and viral load, it is possible that rapid recombination potentiates a higher viral load, and not that higher viral loads cause more coinfection and subsequent recombination. It is also possible that a third underlying factor causes both higher viral load and more frequent recombination. While we cannot directly disentangle causation in this study, the association still suggests that we must consider how recombination rates vary within and between individuals. (ii) We analyzed a relatively small cohort of untreated participants, many of whom showed some degree of virologic control (Zanini et al. 2015). The same features that may permit these individuals to control the virus may also affect recombination processes within these viral populations. However, we note that individuals with low viral loads showed recombination rates consistent with other estimates in the literature (Neher and Leitner 2010; Batorsky et al. 2011), while time points with less control exhibited elevated recombination rates more consistent with in vitro studies (Levy et al. 2004; Schlub et al. 2010). (iii) Most of our analysis derives from only a single data set (Zanini et al. 2015) and we could not locate other datasets with the necessary characteristics (longitudinal, deeply sequenced, abundant segregating sites, minimal PCR recombination, range of viral loads) for a full biological replicate. However, application to Caskey et al. (2017) did produce a consistent estimate with the low viral load group. We are hopeful that as sequencing technologies proliferate, new data will be collected that permit further verification.

While RATS-LD is a broadly useful technique for quantifying rapid recombination from time series data, the sequencing strategy (especially the read length and sampling time intervals) limits the recombination rates that can be effectively inferred. Similar limitations about read lengths have been noted in other work estimating linkage structure from pooled read data (Feder et al. 2012). Low recombination rates may permit few or no recombination events to occur within the context of single reads. As a result, little breakdown of linkage will be observable and longer read sequencing data might be necessary to capture these events. As these sequencing approaches proliferate (Gallardo et al. 2021), we expect the utility of linkage-based techniques for quantifying recombination and other evolutionary forces to expand, even in populations with lower recombination rates. In the opposite direction, high recombination rates can result in extremely rapid decay of linkage which may saturate even between nearly adjacent loci if the sampling time points are insufficiently dense (see $\rho =10^{-3}$). This problem is potentially resolvable by denser sampling, although we note that the recombination rates explored here are some of the highest measured in vivo (Pérez-Losada et al. 2015). More broadly, if intervals between samples are too long and the population has returned to equilibrium, differential rates of recombination between SNPs separated by different distances will not be visible. In this way, distance and time affect linkage signals differently, despite appearing together in equation (1). For the purpose of HIV, RATS-LD is well suited to the current state of short read paired-end next generation sequencing data and the 150-300 generation time intervals examined.

Although we found that RATS-LD was appropriate for our uses in this paper, we offer a series of recommendations for assessing its appropriateness in other settings and the trustworthiness of its estimates. *(i) Assess sampling characteristics:* we found RATS-LD performed best with at least 500 segregating sites and a sampling depth above 40 (supplementary Figs. S8A and S11, Supplementary Material online). *(ii) Understand the $\hat{\rho }$ performance range:* with paired-end data spanning approximately 700 base pairs sampled on the order of 150 to 300 generations apart, we found that RATS-LD can discriminate well among recombination rates in the range of $5\times 10^{-6}$ to $5\times 10^{-4}$. Rates estimated outside of this range are generally untrustworthy under these sampling conditions. Longer range or temporally denser sequencing data may expand this accuracy range in the future. *(iii) Visualize linkage decay curves:* the raw data underlying these curves can be noisy, so we found binning by 500 bp × generation moving windows for visualization balanced stochasticity and signal. If these running average curves appear flat, RATS-LD will likely be unable to accurately estimate recombination rates. *(iv) Examine bootstraps:* bimodal bootstraps or those spanning multiple orders of magnitude reflect poor curve fitting and should be treated with caution.

More broadly, the biological phenomenon that we report here - association between higher recombination rate and higher population density - could be much more widespread in facultatively asexual populations where physical proximity can mediate the rate of recombination. Potentially relevant cases include viral homologous recombination (as shown here) and reassortment, but also rates of plasmid conjugation in bacteria, or sexual reproduction in hermaphroditic organisms or plants that do not self or outcross exclusively. Explicit attention to population density in mediating recombination rates stands to impact our understanding of species diversification in viruses and more broadly.

## Materials and Methods

### Measuring Linkage Decay over Time to Estimate *ρ*

RATS-LD quantifies the linkage between pairs of segregating loci using $D^{\prime }$ (Lewontin 1988):


(4) $$D^{\prime }=\frac{\left|p_{12}-p_{1}p_{2}\right|}{D_{\max}}$$

where $p_{1}$ and $p_{2}$ are the frequencies of the majority nucleotide at the corresponding locus, $p_{12}$ is the frequency of the haplotype made up by the majority nucleotides at the two loci, and $D_{\max}$ is given by


$$D_{\max}=\left\{ \begin{array}{cc} \text{min}(p_{1}p_{2},(1-p_{1})(1-p_{2})) & \text{if}\;p_{12}<p_{1}p_{2}\\ \text{min}(p_{1}(1-p_{2}),(1-p_{1})p_{2}) & \text{if}\;p_{12}>p_{1}p_{2}. \end{array}\right.$$

Estimation of *ρ* can also be performed using *D* rather than $D^{\prime }$, but we found $D^{\prime }$ was less affected by noise (supplementary Fig. S16, Supplementary Material online). Because linkage information is necessary to compute $D^{\prime }$, pairs of SNPs must be close enough to be spanned by a sequencing read.

We applied stringent filtering criteria (see below) to build a set of SNPs separated by a given distance (*d*) and sampled at two different times Δt generations apart for which time point-specific $D^{\prime }$ values could be computed. We computed LDMs for each pair of SNPs across two time points using the negative log ratio of their $D^{\prime }$ values, as given in equation (2). For the in vivo analysis, we converted the number of days between samples to generations by assuming an approximate rate of 1 generation per 2 days (Perelson et al. 1996; Rodrigo et al. 1999).

Using this set of SNP pairs, RATS-LD quantifies the recombination rate by fitting the relationship between LDM and time-scaled distance (dΔt) then calculating the derivative of the fit near dΔt=0. Because the linkage decay between any individual pair of SNPs is noisy over time, we aggregate LDMs across many pairs of SNPs over different distances to improve the fit. Instead of fitting the simple decay equation given in equation (2), we fit LDMs in simulated and in vivo data to the form of equation (3) which better captures the stationary linkage dynamics between two loci at longer time-scaled distances. Our estimate of the recombination rate *ρ* is the derivative of equation (3) at dΔt=0, which is given by $c_{1}\times c_{2}$.

### Curve Fitting

For all analyses except those shown in supplementary Figs. S2 and S3, Supplementary Material online, we fit the curve form (3) to the LDM data via non-linear least squares (Virtanen et al. 2020). Prior to performing the fits, we restricted the data to pairs of SNPs with $d\Delta t<5\times 10^{4}\text{bp}\times \text{generations}$ to boost RATS-LD’s performance in the range of previous HIV recombination rate estimates (supplementary Fig. S11A, Supplementary Material online).

To confirm that the slope of the linkage decay curve near dΔt=0 does capture the true recombination rate, we performed linear fits to the LDM data which are shown in supplementary Figs. S2 and S3, Supplementary Material online. Since the linear fits are only accurate when considering LDMs prior to saturation, we performed these fits to data only including LDMs with dΔt<500,1,000,2,000,10,000,or50,000bp×generations so that at least one dΔt fit threshold would capture the presaturation behavior for each value of *ρ* in the simulated data. We fit these relationships with linear regression (Virtanen et al. 2020) and the slope of the fit was the estimated $\hat{\rho }$.

### Bootstrapped Confidence Intervals

To create bootstrapped confidence intervals for RATS-LD’s recombination rate estimates, we sampled the segregating loci with replacement and repeated estimation using only the LDMs where both loci were sampled. Each bootstrap sample included the same number of segregating loci as was initially used for estimation. The example fits displayed for the bootstrapped confidence intervals use the set of coefficients that provided the estimate at the given quantile (2.5%, 50%, 97.5%) of the $\hat{\rho }$ distribution (rather than the quantiles of the coefficient distributions themselves). The primary $\hat{\rho }$ reported for each analysis is the median of the bootstrapped distribution.

### Simulated Data

To validate RATS-LD’s ability to quantify recombination rates, we simulated populations with known recombination rates using SLiM (Haller and Messer 2019). We performed RATS-LD’s initial validation on a simulated genomic region of length 700 bp, with a mutation rate of $\mu =10^{-6}$ mutations/bp/generation (Achaz et al. 2004; Abram et al. 2010; Sanjuán et al. 2010) and effective population size of $N_{e}=10^{4}$ (Achaz et al. 2004; Feder et al. 2017). The simulated recombination rates *ρ* ranged from $2\times 10^{-6}$ to $10^{-3}$ recombinations/bp/generation to broadly encapsulate the range of previously estimated HIV recombination rates (Shriner et al. 2004; Neher and Leitner 2010; Batorsky et al. 2011). For the purpose of the discrimination analysis only (Fig. 2D and E), we also ran neutral simulations in an expanded range of $\rho =10^{-6}$ to $\rho =5\times 10^{-3}$. Each simulation was run for $5\times 10^{4}$ generations before samples from five evenly spaced time points were collected at intervals of 150 generations. Each sample contained M=800 genomes to approximately match the median number of templates in each of the in vivo sequencing reactions (Zanini et al. 2015). RATS-LD requires many pairs of SNPs across multiple time points to fit equation (3). Therefore, data pooling across simulations is required for estimation. Since our power analysis indicated that estimation is most accurate when there are >500 loci available for estimation (supplementary Fig. S11C, Supplementary Material online), for each recombination rate estimate we grouped 5 simulations and sampled 200 segregating loci from each simulation to form groups with ≈1,000 segregating loci (supplementary Table S1, Supplementary Material online).

To assess RATS-LD’s performance in the presence of selection, we performed additional simulations with selection coefficients designed to mimic intrahost HIV evolution more closely. These selection simulations included a genomic region of length 700 bp, mutation rate $\mu =10^{-5}$ mutations/bp/generation (Achaz et al. 2004; Abram et al. 2010; Sanjuán et al. 2010), and effective population size $N_{e}=10^{4}$ (Achaz et al. 2004; Feder et al. 2017). Selection coefficients were drawn from the following distribution of fitness effects: a small number of mutations (≈0.1%) were strongly beneficial with s=0.05. Most mutations (≈70%) were non-synonymous mutations (Kimura 1968) and were strongly deleterious with selection coefficient s=−0.1 (Zanini et al. 2017). The remaining ≈30% of mutations represented synonymous mutations and were neutral with s=0 (frequency ≈10%) or slightly deleterious with s=−0.01 (frequency ≈20%) (Zanini et al. 2017). Each simulation was run for 50 generations before 9 evenly spaced samples of M=800 templates each were collected at intervals of 150 generations. The number of sampling time points was increased to 9 so that the datasets would encompass the maximum length of sampling for participants in the in vivo data set (Zanini et al. 2015). Under these simulated conditions, the proportion of segregating sites per time point pair in the selection simulations matched the in vivo data much more closely than the neutral simulations (supplementary Fig. S17, Supplementary Material online). For each estimate, we grouped 50 simulation runs which provided 700 to 2,000 segregating loci to ensure that RATS-LD was well powered (supplementary Table S1, Supplementary Material online). Due to the simulations’ shorter genomic length (700 bp) vs. the average in vivo fragment length (mean ≍ 1,800 bp), a group of 10 simulations is approximately equivalent to the data from one participant. The selection simulations described in this paragraph are referred to as the “baseline selection simulations.”

We also performed selection simulations to test for impacts of other population factors on RATS-LD estimates. First, we tested the impact of population substructure by simulating populations consisting of two separate subpopulations at $\rho =10^{-4}$ and $\rho =10^{-5}$. Viruses migrated between the two subpopulations with backward migration rates of $m=10^{-5}$, $10^{-4}$, $10^{-3}$, and $10^{-2}$. The extremes of these values were drawn from the literature (Lythgoe et al. 2016; Feder et al. 2019). Each subpopulation had size $5\times 10^{3}$ resulting in full population sizes of $N_{e}=10^{4}$ (consistent with the baseline selection simulations). Both subpopulations contributed equally to the sampled viruses. All other parameters in the substructure simulations matched those in the baseline selection simulations. Second, we tested the impact of varying effective population size. These simulations had the same parameters as the baseline selection simulations except that we simulated a wider range of effective population sizes ($N_{e}=5\times 10^{3}$, $10^{4}$, $5\times 10^{4}$, $10^{5}$, and $5\times 10^{5}$) for a subset of recombination rates ($\rho =2\times 10^{-6}$, $10^{-5}$, $2\times 10^{-5}$, $10^{-4}$, $2\times 10^{-4}$, and $10^{-3}$). These $N_{e}$ values were selected based on variation in estimates of short-term $N_{e}$ of intrahost HIV in the literature (Achaz et al. 2004; Feder et al. 2017). An $N_{e}$ value of $10^{3}$ was also tested, but these simulations yielded linkage decay curves that were too noisy for estimation under RATS-LD’s standard filtering conditions. Third, we tested the impact of varying sampling depths. These simulations have the same parameters as the baseline selection simulations, but we varied the sampling depth of *M* to be 10, 20, 40, and 80. At lower sampling depths, many more simulations (up to 5,000 per estimation at M=10) were required to form groups of 500 to 1,000 segregating loci. Since larger numbers of independent observations reduce noise and improve RATS-LD accuracy, these estimates represent best case scenarios for each sampling depth.

To match the sequencing coverage in the in vivo data, we created a procedure to downsample the simulations to mimic the pairwise coverage of two loci *d* nucleotides apart. To do this, we created an empirical distribution of reads overlapping a locus at an arbitrary position 0 (supplementary Fig. S18A, Supplementary Material online). First, we simulated the expected positions of $10^{5}$ reads with start positions uniformly distributed between genomic positions −700 and 0 (in bp). For each start position, we uniformly sampled a total end to end read length between 400 and 700 bp, which was the observed read length distribution in Zanini et al. (2015). In addition, we only considered 300 bp of coverage extending inwards from each end of the read, to match the paired-end sequencing study design (Zanini et al. 2015, 2017). As a result, longer reads did not have coverage in their centers. We discarded any simulated reads that did not capture genomic position 0. At this point, we formed the empirical distribution by computing the number of reads that overlapped position 0 and a position *d* nucleotides away in either direction. The resulting empirical distribution had high coverage for nearby loci and decreasing coverage as *d* increased (supplementary Fig. S18C, Supplementary Material online). This paired-end read sampling technique mimicked the in vivo sampling scheme and provided coverage similar to the in vivo data (Zanini et al. 2015) (supplementary Fig. S18B, Supplementary Material online). Then, for each pair of SNPs in the SLiM data at a distance of *d* nucleotides apart, we downsampled their coverage to match the empirical number of overlapping reads for two SNPs at a distance of *d* nucleotides. This procedure was used to downsample coverage for all simulation scenarios described.

### In Vivo Data

We used longitudinal HIV deep-sequencing data from Zanini et al. (2015) for in vivo recombination rate estimation. The samples were collected from the blood plasma of 11 participants who were diagnosed with HIV-1 in Sweden from 1990 to 2003 and were not on antiretroviral treatment. Six to twelve time points are included per participant with both sequencing and viral load information at most time points. The sequences span the full HIV genome and were generated using 6 overlapping amplicons, but we excluded fragment 5 (which contains *env*) in our analysis due to its lower rates of template recovery. The sequencing reactions used paired-end reads amplified via a protocol designed to minimize PCR recombination (see Discussion). In particular, the protocol used only a single round of PCR (rather than a nested approach). We used arrays of co-occurrences of alleles across pairs of loci (available at https://hiv.biozentrum.unibas.ch), which had undergone Zanini et al.’s quality assurance process for read mapping and filtering. This process included rigorous checks for base quality, mapping errors, and sample contamination. We also used the estimated dates of infection (EDIs) as provided by Zanini et al., and each time point was measured relative to the EDI for the corresponding participant.

To assess if higher viral load is associated with a difference in recombination rate, we sorted pairs of time points into groups based on their average viral load. We started by matching genotype information from each sampling time point with a viral load measurement near the same time. For the majority of the data, the date of sample collection exactly matched the date of the viral load measurement. However, some samples from participants 4 and 7 did not have a concurrent viral load measurement and were therefore matched to viral load measurements within 100 days of the sample’s collection. Additionally, data from Participant 10 was omitted from the analysis due to nearly all of the time points lacking viral load measurements within 100 days. For each pair of time points, we calculated the average viral load by taking the mean of the viral load measurements matched to those two samples. For pairs of time points with intervening viral load measurements, we required that those viral loads stayed within the bounds of the viral loads measured at the starting and ending time points. Pairs of time points with an intervening measurement that violated these boundaries were excluded from the analysis to ensure that the average viral loads used to group the data are as accurate as possible.

For the quantile analyses, the LDMs were labeled with the average viral loads across their corresponding time point pairs and divided based on average load to make two (Fig. 5 and supplementary Fig. S15, Supplementary Material online) or three (Fig. 4 and supplementary Fig. S12, Supplementary Material online) groups with approximately equal numbers of LDMs (supplementary Table S1, Supplementary Material online). Since a single pair of time points could have many corresponding LDMs, the size of the groups sometimes varied by ≈1,000 measurements since all LDMs per time point pair were included in the same group. For the threshold analysis shown in supplementary Figs. S13 and S14, Supplementary Material online, the pairs of time points were placed into groups based on whether their average viral load was above or below a given threshold, and we then applied RATS-LD separately to the high and low viral load groups.

We also performed three additional checks to confirm our observations. First, to ensure the pattern observed in Fig. 4C was not solely driven by data from a single participant, we performed a leave-one-out analysis in which we repeated estimation excluding all data from one participant at a time. Second, to ensure that our results were not driven by two individuals who experienced superinfection, we performed an analysis leaving out both of these individuals simultaneously. Third, we analyzed a dataset of viral sequences described in Caskey et al. (2017), which served as a partial biological replicate. These sequences were collected from 15 study participants with rebounding viral populations after a single infusion of a broadly neutralizing antibody treatment. All participants were either ART naive or were off ART for 8 weeks prior to the study start. We used only the data produced via single genome sequencing, which provided an average sequencing depth of ≈23 reads. We aligned the sequences using the LANL Codon Alignment v2.1.0 tool (https://www.hiv.lanl.gov) and manually checked alignments before proceeding with the estimation process. Additionally, we used only the data starting at 4 weeks post-treatment, when viral populations were rebounding. Prior to the estimation, we grouped the full data set due to the low sequencing depth and relatively low viral loads (supplementary Fig. S8B, Supplementary Material online).

### Data Filtering

We started with data from all time points and all pairs of SNPs which were close enough to be spanned by sequencing reads. Then, we used several filtering steps in both the simulated and in vivo datasets to reduce noise. (i) We required that at least 10 reads span a pair of loci for that pair to be included in the analysis. (ii) SNPs at each time point were included only if the minor allele had a frequency >1% at that time point. We chose this cutoff since Zanini et al. (2015) report that SNP frequencies can be estimated down to 1% accuracy in the in vivo dataset we analyzed. Any SNPs transiently under 1% frequency were not included in the analysis at the corresponding time point, but were included at other time points where they reached the frequency threshold. In the Caskey et al. dataset, we used a >10% allele frequency cutoff due to its lower sampling depth. For the linkage analysis displayed in supplementary Fig. S6, Supplementary Material online, we required a minor allele frequency >20% to replicate the filtering conditions used to generate supplementary Fig. 7 of Zanini et al. (2015). Filtering step 2 prevented false linkage signals from very low frequency alleles appearing and disappearing between time points. (iii) All four possible haplotype combinations of the major and minor alleles at a pair of loci had to be observed at a given time point to be included in the analysis at that time point. Exception: for the analysis with the *D* statistic (supplementary Fig. S16, Supplementary Material online), we did not require that all four possible haplotypes were present. (iv) Because the investigation focused on the decay of linkage across pairs of time points, we required that $D^{\prime }>0.2$ at the first time point in each pair (or D>0.0075 for the analysis using D). RATS-LD’s ability to discriminate between underlying recombination rates is robust to changes in this threshold. (v) Lastly, we excluded a small number of time point pairs that spanned less than 50 days, since the signal of linkage decay at such short timescales is also very susceptible to noise. While we did not explicitly filter out large time step differences between time point pairs, nearly all LDMs (92% of the in vivo LDMs, 99.29% of the neutral simulated LDMs, and 99.53% of the selected simulated LDMs) were from SNP pairs separated by 130 to 300 generations. An additional 4% on in vivo LDMs were separated by 50 to 130 generations. For counts of segregating loci and their corresponding LDMs which passed these filtering steps, consult supplementary Tables S1 and S2, Supplementary Material online.

### Discrimination Analysis

To validate that RATS-LD can discriminate between different recombination rates, we randomly paired simulations with recombination rates that were a factor of 1, 1.1, 1.2, 1.5, 2, or 5 apart. For each value of *ρ* we used 20 datasets of ≈1,000 neutrally evolving loci which were simulated in SLiM as described above. We applied RATS-LD to each simulated dataset to produce a $\hat{\rho }$ estimate given by the median of its corresponding bootstrapped distribution (1,000 bootstraps). To compare two given *ρ* values, we randomly paired these estimates to create 20 matched pairs. For each matched pair with true recombination rates $\rho_{1}$ and $\rho_{2}$, we computed two comparison metrics. (i) Simple rank order: we determined whether the individual estimates $\hat{\rho_{1}}$ and $\hat{\rho_{2}}$ were correctly ordered such that $\hat{\rho_{1}}>\hat{\rho_{2}}$ if $\rho_{1}>\rho_{2}$. If $\rho_{1}=\rho_{2}$, we checked agreement against an arbitrary ordering. (ii) Bootstrapped significance: we determined whether the bootstrapped $\hat{\rho }$ distributions from the datasets overlapped. We reshuffled the pairings and recomputed the two metrics 500 times.

## Supplementary Material

msad260_Supplementary_Data

## Data Availability

The primary dataset used in this study (viral loads and paired participant read counts from Zanini et al. 2015) can be downloaded from https://hiv.biozentrum.unibas.ch/. The single genome sequencing data from Caskey et al. (2017) can be downloaded from genbank (accession numbers KY323724–KY324834), while the viral load measurements from the study are listed in Supplementary Table 3 of Caskey et al. (2017). All code to reproduce these analyses can be found at https://github.com/federlab/hiv-recombination.

## References

[msad260-B1] Abram ME , Ferris AL, Shao W, Alvord WG, Hughes SH. Nature, position, and frequency of mutations made in a single cycle of HIV-1 replication. J Virol. 2010:84(19):9864–9878. 10.1128/JVI.00915-10. 20660205 PMC2937799

[msad260-B2] Achaz G , Palmer S, Kearney M, Maldarelli F, Mellors JW, Coffin JM, Wakeley J. A robust measure of HIV-1 population turnover within chronically infected individuals. Mol Biol Evol. 2004:21(10):1902–1912. 10.1093/molbev/msh196. 15215321

[msad260-B3] An W , Telesnitsky A. Effects of varying sequence similarity on the frequency of repeat deletion during reverse transcription of a human immunodeficiency virus type 1 vector. J Virol. 2002:76(15):7897–7902. 10.1128/JVI.76.15.7897-7902.2002. 12097604 PMC136404

[msad260-B4] Balakrishnan M , Fay PJ, Bambara RA. The kissing hairpin sequence promotes recombination within the HIV-I $5^{\prime }$ leader region. J Biol Chem. 2001:276(39):36482–36492. 10.1074/jbc.M102860200. 11432862

[msad260-B5] Balakrishnan M , Roques BP, Fay PJ, Bambara RA. Template dimerization promotes an acceptor invasion-induced transfer mechanism during human immunodeficiency virus type 1 minus-strand synthesis. J Virol. 2003:77(8):4710–4721. 10.1128/JVI.77.8.4710-4721.2003. 12663778 PMC152154

[msad260-B6] Batorsky R , Kearney MF, Palmer SE, Maldarelli F, Rouzine IM, Coffin JM. Estimate of effective recombination rate and average selection coefficient for HIV in chronic infection. Proc Natl Acad Sci U S A. 2011:108(14):5661–5666. 10.1073/pnas.1102036108. 21436045 PMC3078368

[msad260-B7] Caskey M , Schoofs T, Gruell H, Settler A, Karagounis T, Kreider EF, Murrell B, Pfeifer N, Nogueira L, Oliveira TY, et al. Antibody 10-1074 suppresses viremia in HIV-1-infected individuals. Nat Med. 2017:23(2):185–191. 10.1038/nm.4268. 28092665 PMC5467219

[msad260-B8] Castro LA , Leitner T, Romero-Severson E. Recombination smooths the time signal disrupted by latency in within-host HIV phylogenies. Virus Evol. 2023:9(1):vead032. 10.1093/ve/vead032. 37397911 PMC10313349

[msad260-B9] Charlesworth B , Charlesworth D. *Elements of evolutionary genetics*. Greenwood Village (CO): Roberts & Company; 2010.

[msad260-B10] Chen J , Nikolaitchik O, Singh J, Wright A, Bencsics CE, Coffin JM, Ni N, Lockett S, Pathak VK, Hu W-S. High efficiency of HIV-1 genomic RNA packaging and heterozygote formation revealed by single virion analysis. Proc Natl Acad Sci U S A. 2009:106(32):13535–13540. 10.1073/pnas.0906822106. 19628694 PMC2714765

[msad260-B11] Feder AF , Kline C, Polacino P, Cottrell M, Kashuba ADM, Keele BF, Hu S-L, Petrov DA, Pennings PS, Ambrose Z. A spatio-temporal assessment of simian/human immunodeficiency virus (SHIV) evolution reveals a highly dynamic process within the host. PLoS Pathog. 2017:13(5):e1006358. 10.1371/journal.ppat.1006358. 28542550 PMC5444849

[msad260-B12] Feder AF , Pennings PS, Hermisson J, Petrov DA. Evolutionary dynamics in structured populations under strong population genetic forces. G3: Genes Genome Genet. 2019:9(10):3395–3407. 10.1534/g3.119.400605. PMC6778802 31462443

[msad260-B13] Feder AF , Pennings PS, Petrov DA. The clarifying role of time series data in the population genetics of HIV. PLoS Genet. 2021:17(1):e1009050. 10.1371/journal.pgen.1009050. 33444376 PMC7808693

[msad260-B14] Feder AF , Petrov DA, Bergland AO. LDx: estimation of linkage disequilibrium from high-throughput pooled resequencing data. PLoS One. 2012:7(11):e48588. 10.1371/journal.pone.0048588. 23152785 PMC3494690

[msad260-B15] Gallardo CM , Wang S, Montiel-Garcia DJ, Little SJ, Smith DM, Routh AL, Torbett BE. MrHAMER yields highly accurate single molecule viral sequences enabling analysis of intra-host evolution. Nucleic Acids Res. 2021:49(12):e70. 10.1093/nar/gkab231. 33849057 PMC8266615

[msad260-B16] Gelderblom HC , Vatakis DN, Burke SA, Lawrie SD, Bristol GC, Levy DN. Viral complementation allows HIV-1 replication without integration. Retrovirology. 2008:5(1):1–18. 18613957 10.1186/1742-4690-5-60 PMC2474848

[msad260-B17] Haller BC , Messer PW. SLiM 3: forward genetic simulations beyond the Wright–Fisher model. Mol Biol Evol. 2019:36(3):632–637. 10.1093/molbev/msy228. 30517680 PMC6389312

[msad260-B18] Hartl DL , Clark AG, Clark AG. Principles of population genetics. Vol. 116. Sunderland: Sinauer Associates; 1997.

[msad260-B19] Hu W-S , Hughes SH. HIV-1 reverse transcription. Cold Spring Harb Perspect Med. 2012:2(10):a006882. 10.1101/cshperspect.a006882. 23028129 PMC3475395

[msad260-B20] Hu WS , Temin HM. Genetic consequences of packaging two RNA genomes in one retroviral particle: pseudodiploidy and high rate of genetic recombination. Proc Natl Acad Sci U S A. 1990:87(4):1556–1560. 10.1073/pnas.87.4.1556. 2304918 PMC53514

[msad260-B21] Josefsson L , King MS, Makitalo B, Brännström J, Shao W, Maldarelli F, Kearney MF, Hu W-S, Chen J, Gaines H, et al. Majority of CD4+ T cells from peripheral blood of HIV-1 infected individuals contain only one HIV DNA molecule. Proc Natl Acad Sci U S A. 2011:108(27):11199–11204. 10.1073/pnas.1107729108. 21690402 PMC3131354

[msad260-B22] Jung A , Maier R, Vartanian J-P, Bocharov G, Jung V, Fischer U, Meese E, Wain-Hobson S, Meyerhans A. Multiply infected spleen cells in HIV patients. Nature. 2002:418(6894):144. 10.1038/418144a. 12110879

[msad260-B23] Kellam P , Larder BA. Retroviral recombination can lead to linkage of reverse transcriptase mutations that confer increased zidovudine resistance. J Virol. 1995:69(2):669–674. 10.1128/jvi.69.2.669-674.1995. 7529334 PMC188627

[msad260-B24] Kimura M . Genetic variability maintained in a finite population due to mutational production of neutral and nearly neutral isoalleles. Genet Res. 1968:11(3):247–270. 10.1017/S0016672300011459. 5713805

[msad260-B25] Kimura M , Ohta T. *Theoretical aspects of population genetics*. Princeton (NJ): Princeton University Press; 1971. 5162676

[msad260-B26] King SR , Duggal NK, Ndongmo CB, Pacut C, Telesnitsky A. Pseudodiploid genome organization aids full-length human immunodeficiency virus type 1 DNA synthesis. J Virol. 2008:82(5):2376–2384. 10.1128/JVI.02100-07. 18094172 PMC2258930

[msad260-B27] Kreger J , Garcia J, Zhang H, Komarova NL, Wodarz D, Levy DN. Quantifying the dynamics of viral recombination during free virus and cell-to-cell transmission in HIV-1 infection. Virus Evol. 2021:7(1):veab026. 10.1093/ve/veab026. 34012557 PMC8117450

[msad260-B28] Levy DN , Aldrovandi GM, Kutsch O, Shaw GM. Dynamics of HIV-1 recombination in its natural target cells. Proc Natl Acad Sci U S A. 2004:101(12):4204–4209. 10.1073/pnas.0306764101. 15010526 PMC384719

[msad260-B29] Lewontin RC . On measures of gametic disequilibrium. Genetics. 1988:120(3):849–852. 10.1093/genetics/120.3.849. 3224810 PMC1203562

[msad260-B30] Lythgoe KA , Blanquart F, Pellis L, Fraser C. Large variations in HIV-1 viral load explained by shifting-mosaic metapopulation dynamics. PLoS Biol. 2016:14(10):e1002567. 10.1371/journal.pbio.1002567. 27706164 PMC5051940

[msad260-B31] Martin DP , Varsani A, Roumagnac P, Botha G, Maslamoney S, Schwab T, Kelz Z, Kumar V, Murrell B. RDP5: a computer program for analyzing recombination in, and removing signals of recombination from, nucleotide sequence datasets. Virus Evol. 2020:7(1):veaa087. 10.1093/ve/veaa087. 33936774 PMC8062008

[msad260-B32] Maydt J , Lengauer T. Recco: recombination analysis using cost optimization. Bioinformatics. 2006:22(9):1064–1071. 10.1093/bioinformatics/btl057. 16488909

[msad260-B33] Moumen A , Polomack L, Roques B, Buc H, Negroni M. The HIV-1 repeated sequence R as a robust hot-spot for copy-choice recombination. Nucleic Acids Res. 2001:29(18):3814–3821. 10.1093/nar/29.18.3814. 11557813 PMC55921

[msad260-B34] Moutouh L , Corbeil J, Richman DD. Recombination leads to the rapid emergence of HIV-1 dually resistant mutants under selective drug pressure. Proc Natl Acad Sci U S A. 1996:93(12):6106–6111. 10.1073/pnas.93.12.6106. 8650227 PMC39197

[msad260-B35] Neher RA , Leitner T. Recombination rate and selection strength in HIV intra-patient evolution. PLoS Comput Biol. 2010:6(1):e1000660. 10.1371/journal.pcbi.1000660. 20126527 PMC2813257

[msad260-B36] Nikolaitchik OA , Galli A, Moore MD, Pathak VK, Hu W-S. Multiple barriers to recombination between divergent HIV-1 variants revealed by a dual-marker recombination assay. J Mol Biol. 2011:407(4):521–531. 10.1016/j.jmb.2011.01.052. 21295586 PMC3065980

[msad260-B37] Nora T , Charpentier C, Tenaillon O, Hoede C, Clavel F, Hance AJ. Contribution of recombination to the evolution of human immunodeficiency viruses expressing resistance to antiretroviral treatment. J Virol. 2007:81(14):7620–7628. 10.1128/JVI.00083-07. 17494080 PMC1933369

[msad260-B38] Onafuwa-Nuga A , Telesnitsky A. The remarkable frequency of human immunodeficiency virus type 1 genetic recombination. Microbiol Mol Biol Rev. 2009:73(3):451–480. 10.1128/MMBR.00012-09. 19721086 PMC2738136

[msad260-B39] Panganiban AT , Fiore D. Ordered interstrand and intrastrand DNA transfer during reverse transcription. Science. 1988:241(4869):1064–1069. 10.1126/science.2457948. 2457948

[msad260-B40] Perelson AS , Neumann AU, Markowitz M, Leonard JM, Ho DD. HIV-1 dynamics in vivo: virion clearance rate, infected cell life-span, and viral generation time. Science. 1996:271(5255):1582–1586. 10.1126/science.271.5255.1582. 8599114

[msad260-B41] Pérez-Losada M , Arenas M, Galán JC, Palero F, González-Candelas F. Recombination in viruses: mechanisms, methods of study, and evolutionary consequences. Infect Genet Evol. 2015:30:296–307. 10.1016/j.meegid.2014.12.022. 25541518 PMC7106159

[msad260-B42] Rawson JM , Nikolaitchik OA, Keele BF, Pathak VK, Hu W-S. Recombination is required for efficient HIV-1 replication and the maintenance of viral genome integrity. Nucleic Acids Res. 2018:46(20):10535–10545. 10.1093/nar/gky910. 30307534 PMC6237782

[msad260-B43] Ritchie AJ , Cai F, Smith NM, Chen S, Song H, Brackenridge S, Abdool Karim SS, Korber BT, McMichael AJ, Gao F, et al. Recombination-mediated escape from primary CD8+ T cells in acute HIV-1 infection. Retrovirology. 2014:11(1):69. 10.1186/s12977-014-0069-9. 25212771 PMC4180588

[msad260-B44] Robb ML , Eller LA, Kibuuka H, Rono K, Maganga L, Nitayaphan S, Kroon E, Sawe FK, Sinei S, Sriplienchan S, et al. Prospective study of acute HIV-1 infection in adults in East Africa and Thailand. N Engl J Med. 2016:374(22):2120–2130. 10.1056/NEJMoa1508952. 27192360 PMC5111628

[msad260-B45] Rodrigo AG , Shpaer EG, Delwart EL, Iversen AKN, Gallo MV, Brojatsch J, Hirsch MS, Walker BD, Mullins JI. Coalescent estimates of HIV-1 generation time in vivo. Proc Natl Acad Sci U S A. 1999:96(5):2187–2191. 10.1073/pnas.96.5.2187. 10051616 PMC26758

[msad260-B46] Salazar-Gonzalez JF , Bailes E, Pham KT, Salazar MG, Guffey MB, Keele BF, Derdeyn CA, Farmer P, Hunter E, Allen S, et al. Deciphering human immunodeficiency virus type 1 transmission and early envelope diversification by single-genome amplification and sequencing. J Virol. 2008:82(8):3952–3970. 10.1128/JVI.02660-07. 18256145 PMC2293010

[msad260-B47] Sanjuán R , Nebot MR, Chirico N, Mansky LM, Belshaw R. Viral mutation rates. J Virol. 2010:84(19):9733–9748. 10.1128/JVI.00694-10. 20660197 PMC2937809

[msad260-B48] Sarkar S , Romero-Severson E, Leitner T. Migration coupled with recombination explains disparate HIV-I anatomical compartmentalization signals. *bioRxiv*. 2023. 10.1101/2023.04.22.537949.

[msad260-B49] Schlub TE , Smyth RP, Grimm AJ, Mak J, Davenport MP. Accurately measuring recombination between closely related HIV-1 genomes. PLoS Comput Biol. 2010:6(4):e1000766. 10.1371/journal.pcbi.1000766. 20442872 PMC2861704

[msad260-B50] Selik RM , Linley L. Viral loads within 6 weeks after diagnosis of HIV infection in early and later stages: observational study using national surveillance data. JMIR Public Health Surveill. 2018:4(4):e10770. 10.2196/10770. 30401660 PMC6246969

[msad260-B51] Shankarappa R , Margolick JB, Gange SJ, Rodrigo AG, Upchurch D, Farzadegan H, Gupta P, Rinaldo CR, Learn GH, He X, et al. Consistent viral evolutionary changes associated with the progression of human immunodeficiency virus type 1 infection. J Virol. 1999:73(12):10489–10502. 10.1128/JVI.73.12.10489-10502.1999. 10559367 PMC113104

[msad260-B52] Shriner D , Rodrigo AG, Nickle DC, Mullins JI. Pervasive genomic recombination of HIV-1 in vivo. Genetics. 2004:167(4):1573–1583. 10.1534/genetics.103.023382. 15342499 PMC1470992

[msad260-B53] Siepel AC , Halpern AL, Macken C, Korber BT. A computer program designed to screen rapidly for HIV type 1 intersubtype recombinant sequences. AIDS Res Hum Retroviruses. 1995:11(11):1413–1416. 10.1089/aid.1995.11.1413. 8573400

[msad260-B54] Smyth RP , Schlub TE, Grimm AJ, Waugh C, Ellenberg P, Chopra A, Mallal S, Cromer D, Mak J, Davenport MP. Identifying recombination hot spots in the HIV-1 genome. J Virol. 2014:88(5):2891–2902. 10.1128/JVI.03014-13. 24371048 PMC3958072

[msad260-B55] Song H , Giorgi EE, Ganusov VV, Cai F, Athreya G, Yoon H, Carja O, Hora B, Hraber P, Romero-Severson E. Tracking HIV-1 recombination to resolve its contribution to HIV-1 evolution in natural infection. Nat Commun. 2018:9(1):1–15. 10.1038/s41467-018-04217-5. 29765018 PMC5954121

[msad260-B56] Virtanen P , Gommers R, Oliphant TE, Haberland M, Reddy T, Cournapeau D, Burovski E, Peterson P, Weckesser W, Bright J, et al. SciPy 1.0: fundamental algorithms for scientific computing in python. Nat Methods. 2020:17:261–272. 10.1038/s41592-019-0686-2. 32015543 PMC7056644

[msad260-B57] Zanini F , Brodin J, Albert J, Neher RA. Error rates, PCR recombination, and sampling depth in HIV-1 whole genome deep sequencing. Virus Res. 2017:239:106–114. 10.1016/j.virusres.2016.12.009. 28039047

[msad260-B58] Zanini F , Brodin J, Thebo L, Lanz C, Bratt G, Albert J, Neher RA. Population genomics of intrapatient HIV-1 evolution. Elife. 2015:4:e11282. 10.7554/eLife.11282. 26652000 PMC4718817

[msad260-B59] Zanini F , Puller V, Brodin J, Albert J, Neher RA. In vivo mutation rates and the landscape of fitness costs of HIV-1. Virus Evol. 2017:3(1):vex003. 10.1093/ve/vex003. 28458914 PMC5399928

