# Supplementary material for: Elevated HIV Viral Load is Associated with Higher Recombination Rate In Vivo
Source: Mol Biol Evol. 2024 Jan 10;41(1):msad260. doi: 10.1093/molbev/msad260 (PMC10777272; doi:10.1093/molbev/msad260)
Supplement: msad260_Supplementary_Data [file msad260_supplementary_data.zip › RATS-LD_supplement_Final.pdf]

Table S1: **Counts of segregating loci and linkage decay measures per estimation group.** The recorded number of segregating sites includes all unique loci segregating at any time point in the estimation group (i.e., upper half or lower half). As a result, *in vivo* loci can be counted in multiple groups if they segregate in both those groups. The mean and standard deviation of counts are recorded for simulated data.

Estimation Group	# of Segregating Sites	# of LDMs
Neutral Simulations	996 ± 9	66,465 ± 13,591
Selection Simulations	1,812 ± 798	3,993 ± 1,793
Lower Tertile (Fig. 4 Data)	3,285	25,579
Middle Tertile (Fig. 4 Data)	2,530	19,353
Upper Tertile (Fig. 4 Data)	2,306	23,778
Upper Half (Fig. 5 Data Par.1)	758	8,708
Lower Half (Fig. 5 Data Par.1)	570	5,111
Upper Half (Fig. S15 Data Par.3)	670	5,196
Lower Half (Fig. S15 Data Par.3)	558	4,594
Upper Half (Fig. S15 Data Par.4)	556	5,661
Lower Half (Fig. S15 Data Par.4)	525	3,119
Upper Half (Fig. S15 Data Par.7)	996	14,800
Lower Half (Fig. S15 Data Par.7)	876	10,045

Table S2: **Counts of segregating loci and linkage decay measures per participant.** The recorded number of segregating sites includes all unique loci that were segregating at any time point.

	# of Segregating Sites	# of LDMs
Participant 1	823	12,194
Participant 2	308	1,335
Participant 3	768	8,492
Participant 4	663	8,217
Participant 5	400	3,433
Participant 6	172	546
Participant 7	1,096	23,577
Participant 8	348	1,943
Participant 9	422	4,197
Participant 11	500	4,775

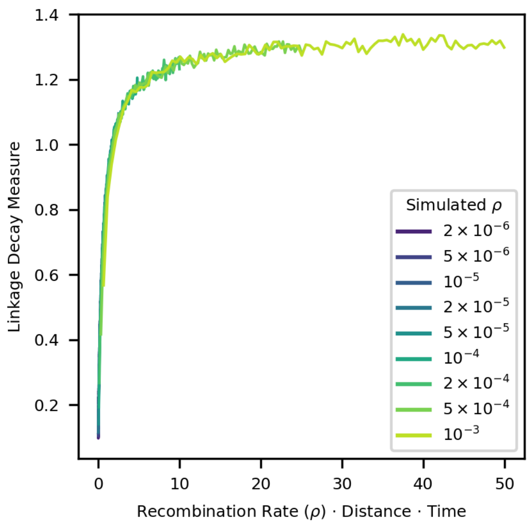

Figure S1: **Average linkage decay measure as a function of recombination rate · distance · time reveals similar decay patterns across simulations with varying ρ.** Binned average LDMs across the neutrally simulated data sets are shown (window size = 500 bp · generations). As expected, these curves collapse on top of each other when the known recombination rate ρ from the simulation is also used as a factor on the x-axis, verifying that the varying saturation patterns in Figure 2A result from recombination rate variation.

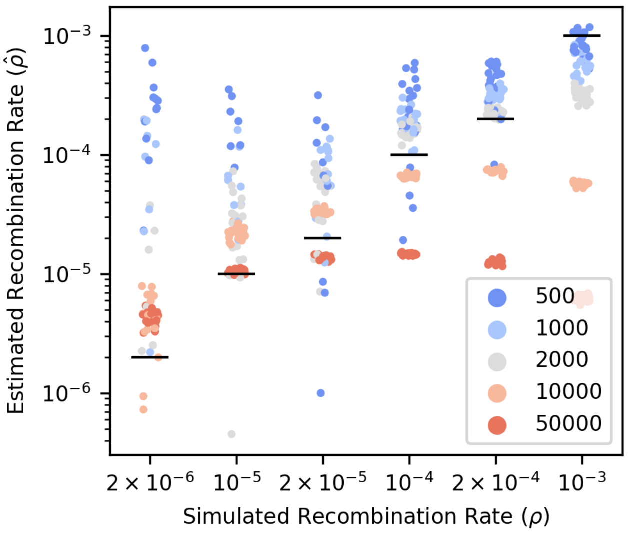

Figure S2: **Fitting lines to linkage decay measures with increasing lengths of distance · time ($d\Delta t$) shows that recombination rates are best recaptured at low $d\Delta t$ for high recombination rates while the opposite is true for low recombination rates.** Note that 77/600 fits resulted in negative estimates which are not shown on this plot due to the necessity for a log scale y-axis. All of the negative estimates were from fits with $d\Delta t$ =< 2000. Examples of the fits used to generate this figure are shown in Figure S3. Each point corresponds to the estimate produced from a single line fit.

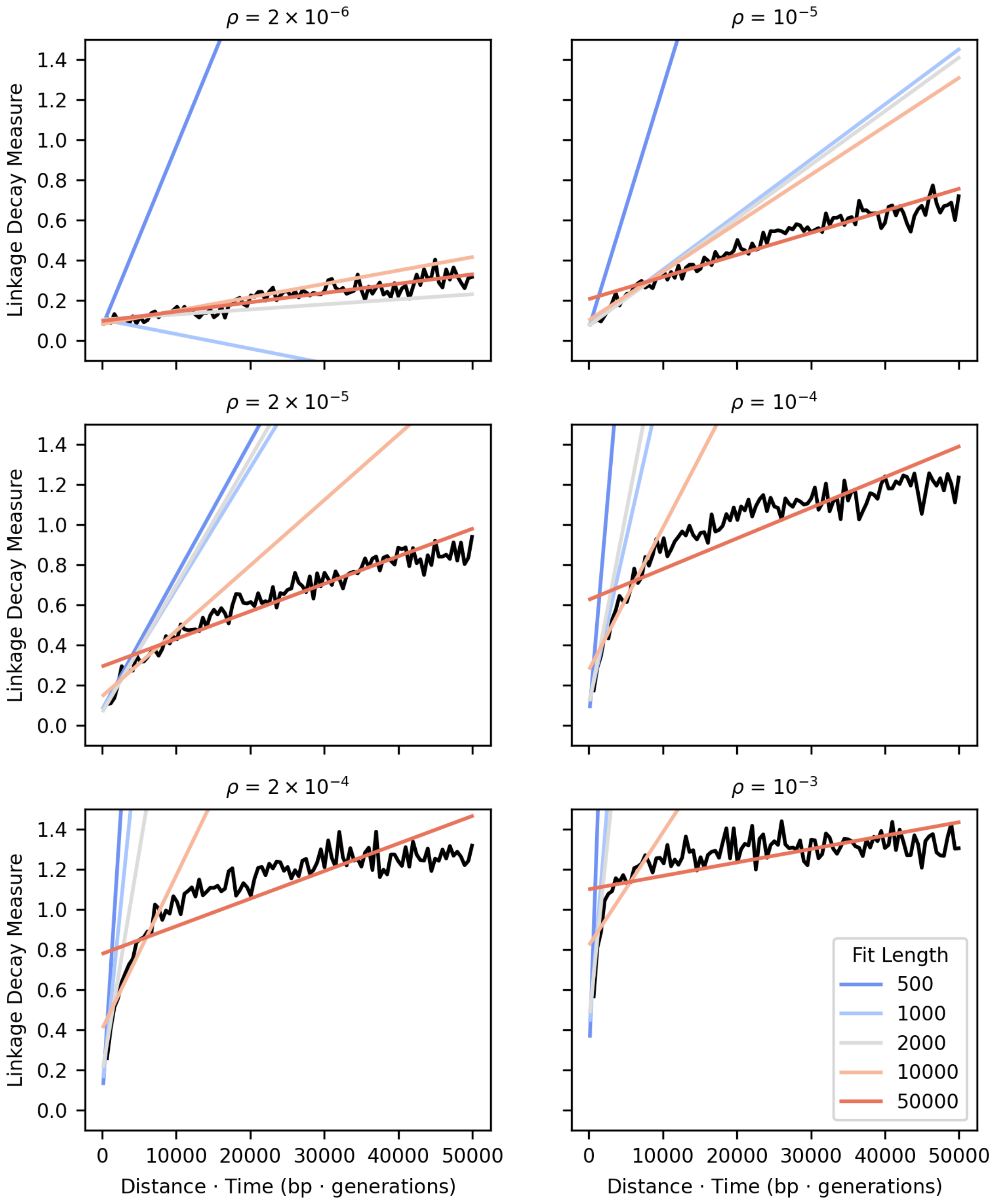

Figure S3: **Example line fits to data truncated at increasing distance · time values capture differing curve behaviors depending on the underlying recombination rate ρ.** The black line in each panel represents a binned average of the linkage decay measures for the panel's corresponding value of ρ (moving average for display purposes only, window size = 500 bp · generations). Linear fits to the LDM data truncated at varying values (shown in different colors), are plotted on top of the binned average.

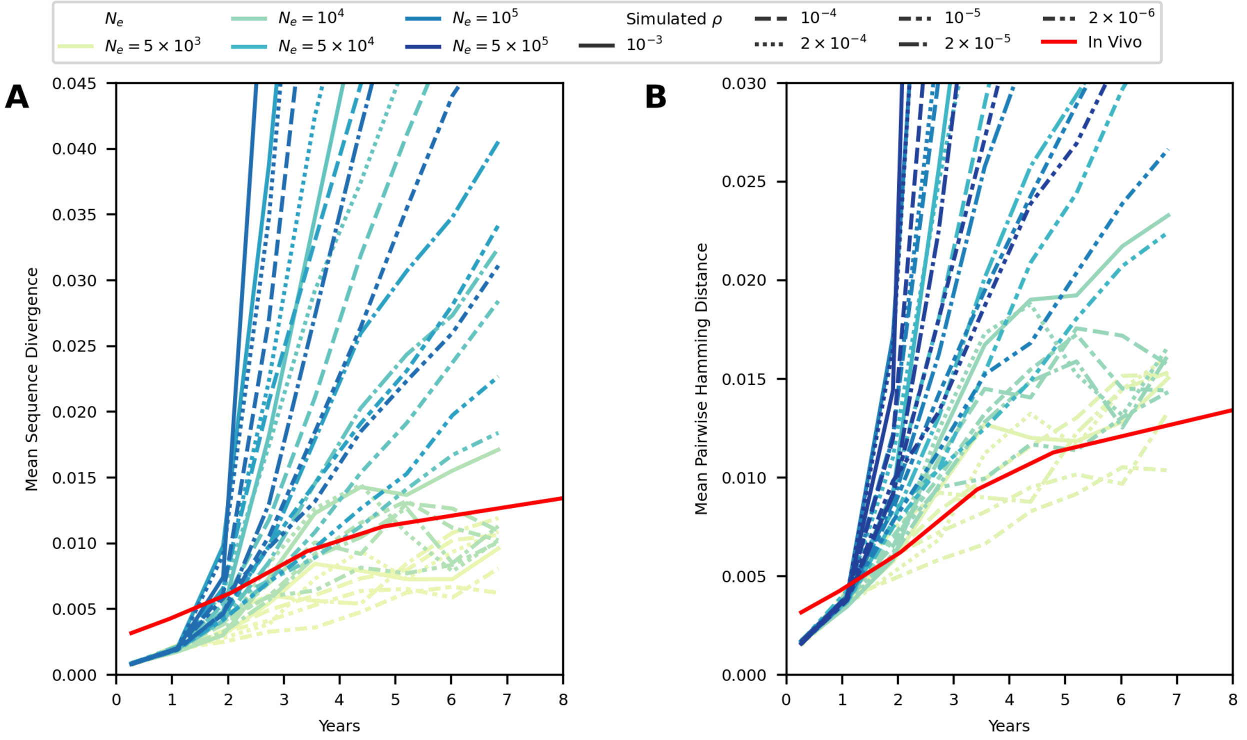

Figure S4: **Selection simulations approximately match sequence diversity and divergence observed *in vivo*.** Mean sequence divergence **(A)** and mean pairwise Hamming distance **(B)** are plotted over time. Selection simulations were performed with a range of effective population sizes ($N_e = 5 \times 10^3$, 10^4, 5×10^4, 10^5 or 5×10^5). Each line type corresponds with the underlying ρ value. Due to the increased simulation time necessary for larger populations, lines for N_e values $< 10^5$ represent data from 500 simulations while lines for N_e values $\geq 10^5$ represent data from 250 simulations. *In vivo* data from Zanini et al. 2015 (Figure 5, panels A and B [58]) are plotted in red for comparison, averaged across all proteins and mutation types.

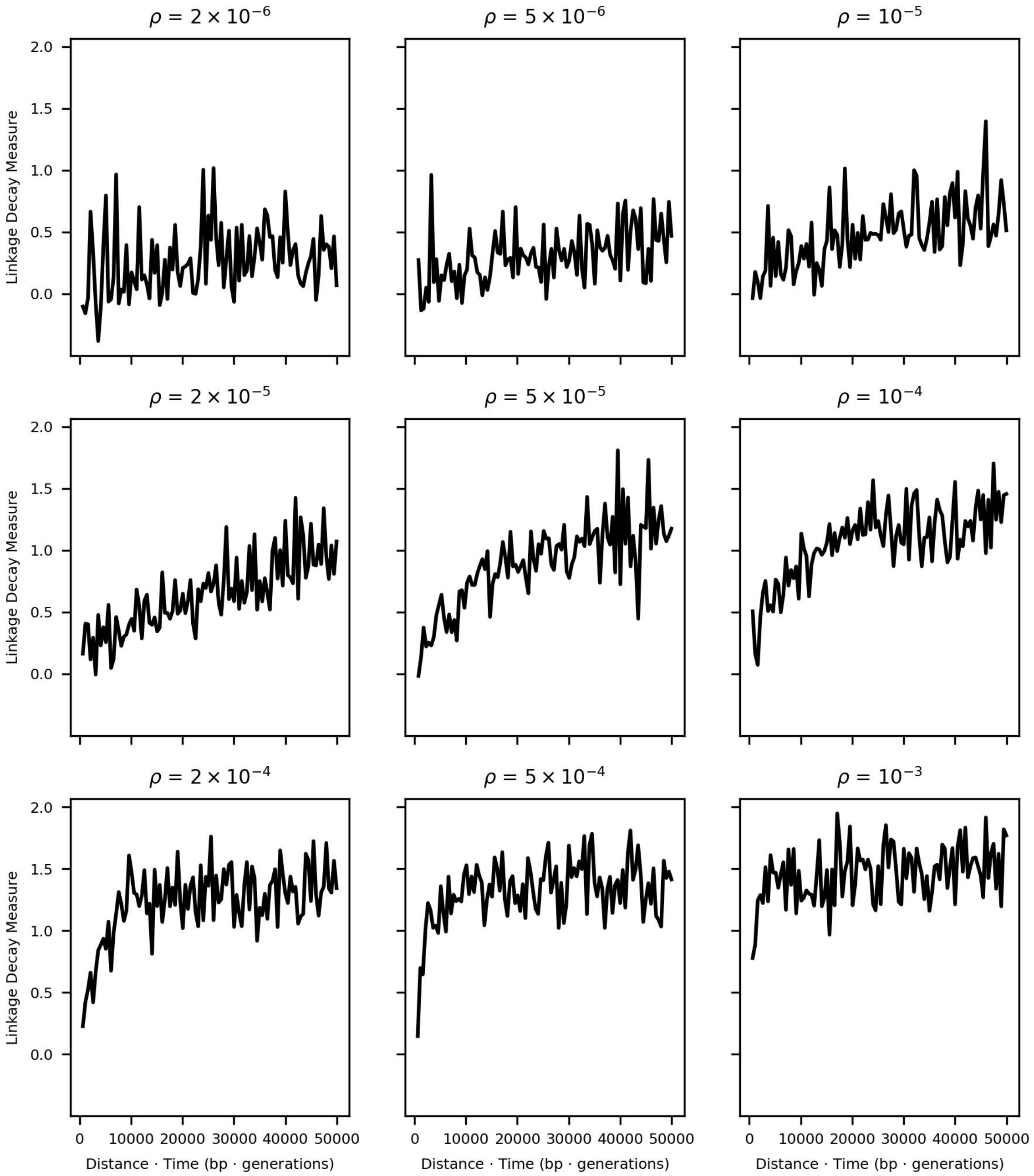

Figure S5: **Example LDM curves for the simulated selection data set.** A single example linkage decay curve is plotted for each simulated ρ value in the selection data set. Each curve represents the binned average LDM (window size = 500bp) from one estimation group. For direct comparisons to the neutral and *in vivo* data sets, see Figures S3 and 4B, respectively.

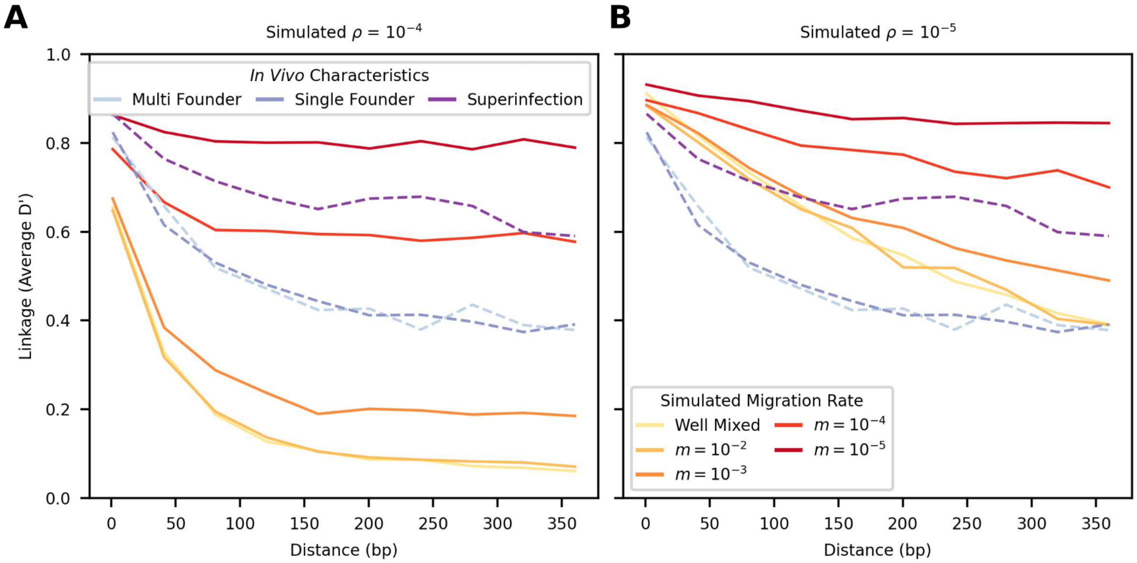

Figure S6: **Simulated scenarios of population substructure encapsulate linkage decay patterns observed *in vivo*.** The average D' value in simulated data sets sampled from two sub-populations interconnected by migration rate m is plotted at different genetic distances for $\rho = 10^{-4}$ **(A)** and $\rho = 10^{-5}$ **(B)**. In each panel, the 'well-mixed' category corresponds to simulated data sets with no population substructure. Each simulated line was formed by taking a binned average (window size = 40bp) of D' across 250 data sets (see Materials & Methods for full simulation and sampling details). The *in vivo* linkage decay is plotted with identical dashed lines on top of both panels for reference. These curves are colored depending on whether the intra-host population had diversity consistent with superinfection (Participants 4 and 7), transmission of multiple closely related founder viruses from the same individual (Participants 3 and 10), or transmission of a single founder virus (all other participants) [58].

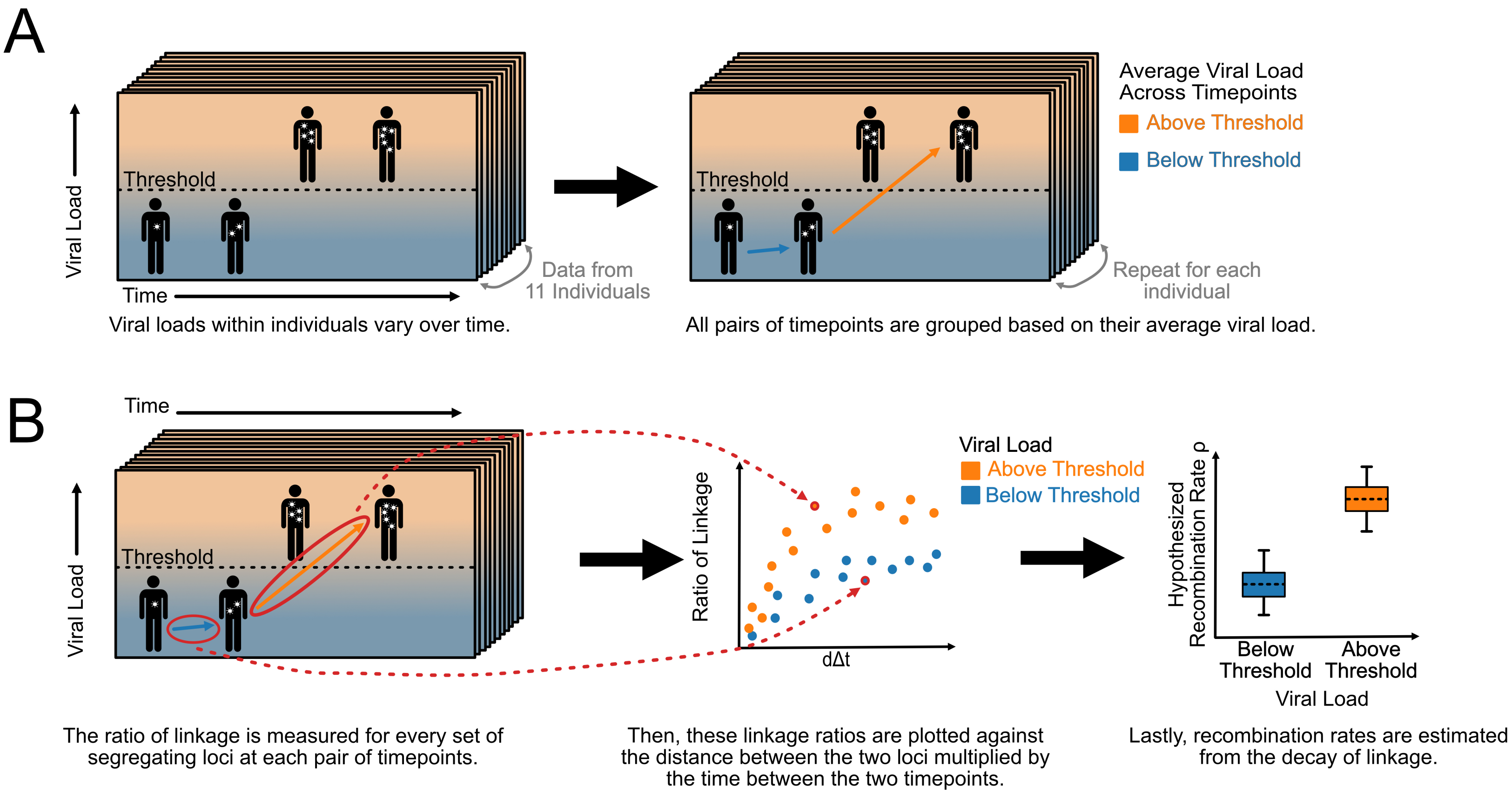

Figure S7: **Workflow for *in vivo* viral load analysis**

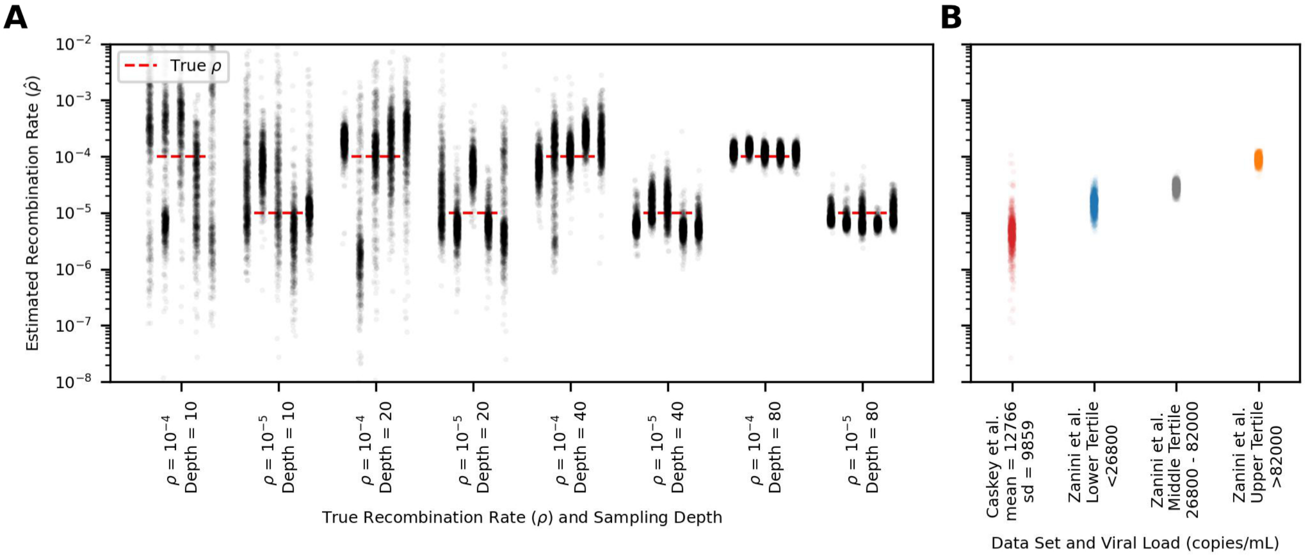

Figure S8: **Low sampling depth is associated with wide ρ bootstraps, but *in vivo* data sets have sufficient depth for RATS-LD estimation.** **(A)** Five estimation groups with 1000 bootstrapped $\hat{\rho}$ values are plotted for multiple sampling depths (10, 20, 40, or 80) and ρ values (10^{-5} or 10^{-4}). **(B)** 1000 bootstraps are plotted for each of the viral load tertiles in the Zanini et al. 2015 dataset [58] alongside a single estimation group for the Caskey et al., 2017 [7] single genome sequencing data set (See Materials & Methods for more details). The cutoff viral load is indicated in the x-axis labels for the Zanini et al. data while the Caskey et al. data set is labeled with the mean and standard deviation of the viral loads associated with LDMs used in its analysis. The variation in the Caskey bootstraps is consistent with variation in the simulated data with approximately the same sampling depth (≈ 23).

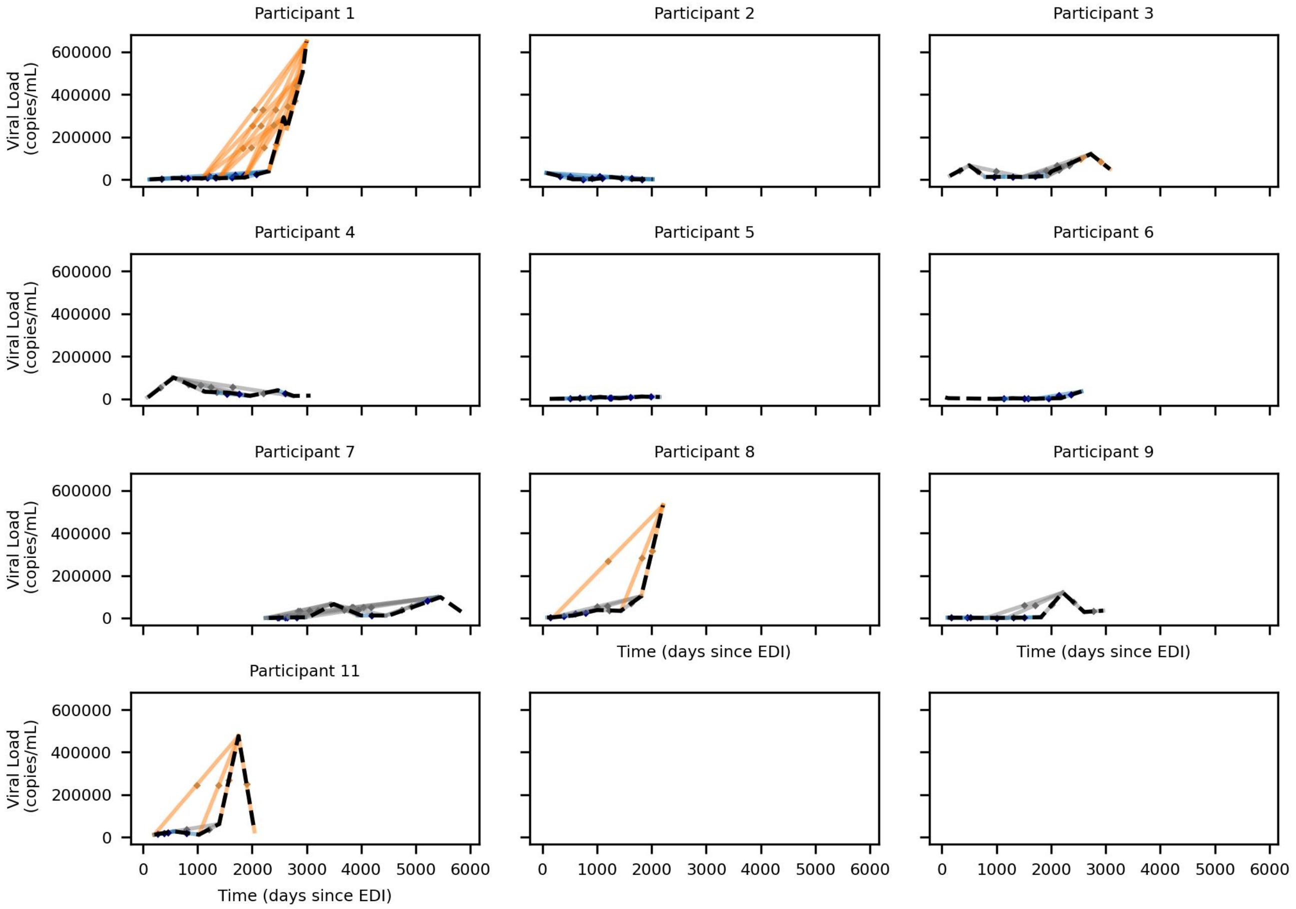

Figure S9: **Viral load trajectories of individuals over time.** In each plot, the black dashed line indicates the viral load measurements over time for a given individual. Each solid line segment connects a pair of time points included in the analysis and the time point pair's average viral load is indicated by a dot at the center of the segment. The segments are colored blue (lower tertile $< 26,800$), gray (middle tertile $26,800 - 82,000$), or orange (upper tertile $> 82,000$) based on their viral load groups.

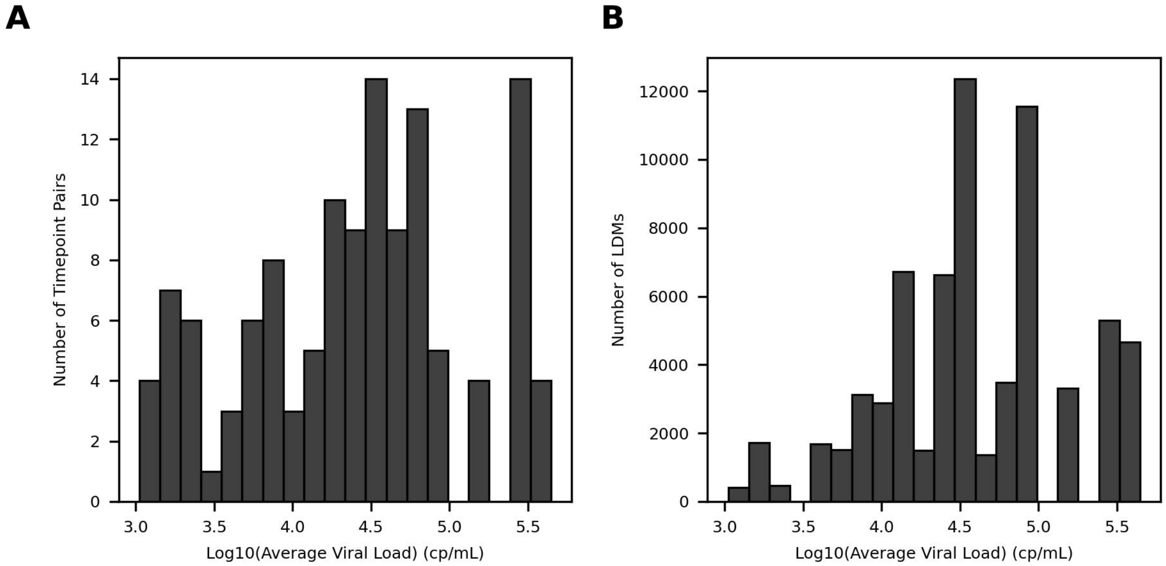

Figure S10: **Distributions of average time point pair viral loads in Zanini et al. [58].** Histogram of viral load measurements averaged over each time point pair used in the analysis (Figure 4) binned by **(A)** number of timepoints and **(B)** number of LDMs.

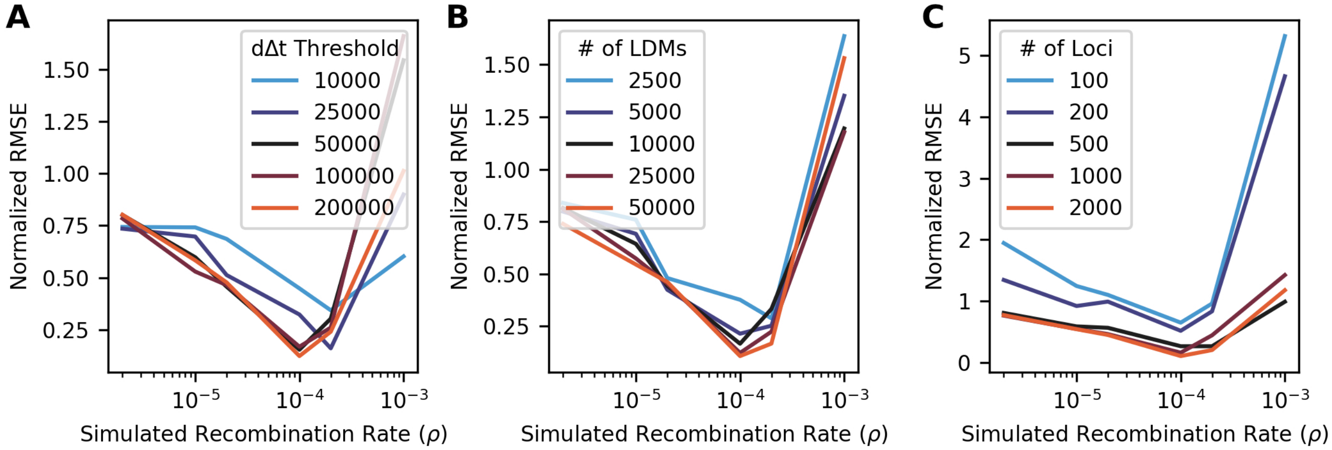

Figure S11: **RATS-LD accuracy under varying data set parameters. (A)** Normalized root mean squared error (RMSE) for RATS-LD estimation when data was truncated at a specified $d\Delta t$ threshold before curve fitting and estimation. Ten simulated data sets were used for the analysis at each ρ value with each data set in this panel containing $\approx 10,000$ LDMs. **(B)** Normalized RMSEs for 10 simulated data sets with varying numbers of linkage decay measures (LDMs) available for estimation. We downsampled each group by including a LDM in the downsampled data set only if both of its corresponding loci had been randomly drawn. Starting with a dataset containing 0 segregating loci, we drew new segregating loci until the total number of LDMs reached the desired data set size. Error decreases as more LDMs are used for estimation. **(C)** Normalized RMSE for 10 data sets with varying numbers of segregating loci. Segregating loci were sampled so each simulation in the data set contributed an equal number of loci. Estimation error decreases as more segregating loci are used for estimation.

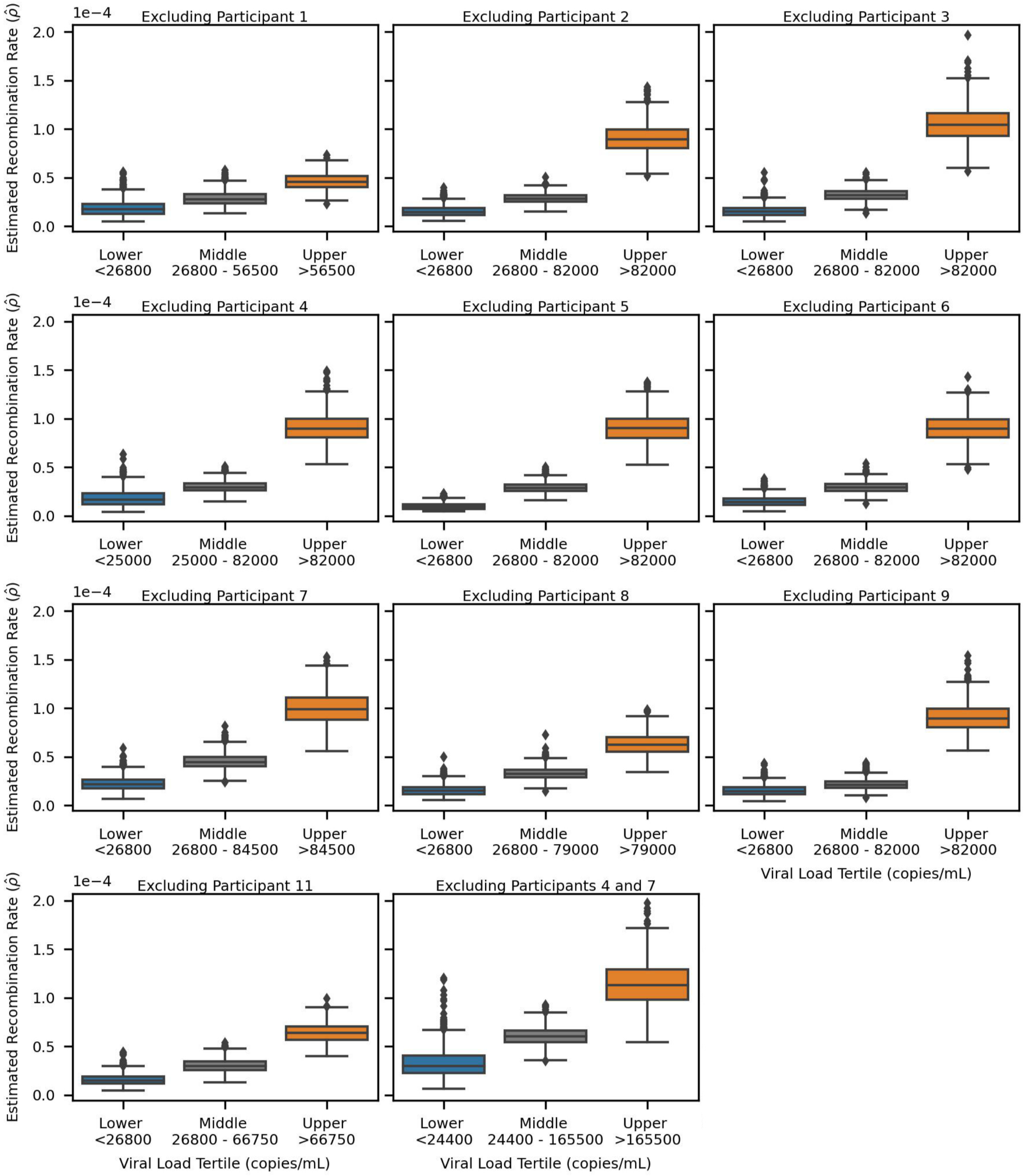

Figure S12: **Recombination rate estimates from *in vivo* leave one out analysis also show a positive association between viral load and recombination rate.** Each subplot indicates the bootstrapped distributions of recombination rate estimates (1000 Boostraps) when RATS-LD is run on all of the *in vivo* data except for loci pairs from the given participant. An additional panel is included where the data from both participants 4 and 7 are excluded since these individuals were superinfected [58] and show longer range linkage preservation than the other individuals (Figure S6). Each data set was split by three even tertiles to create lower, middle, and upper groups based on viral load. Each box indicates the interquartile range of the distribution.

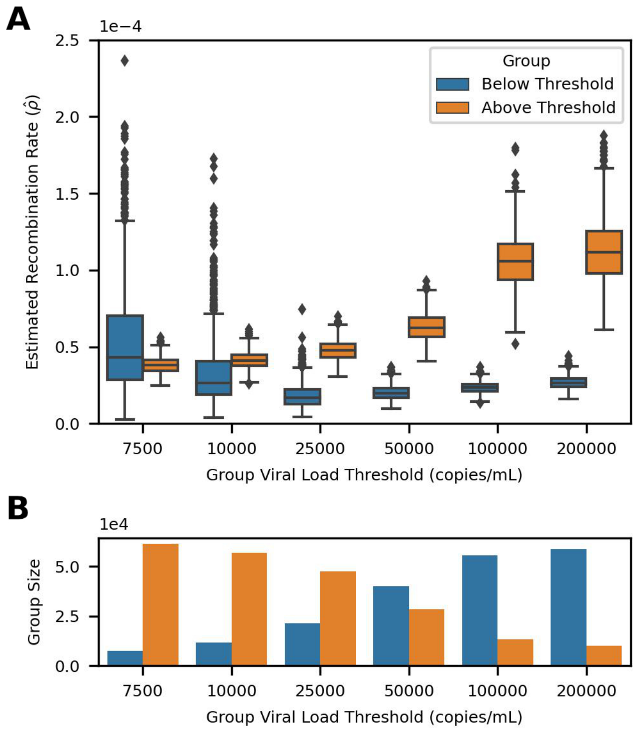

Figure S13: **Using a moving threshold to separate viral load groups reveals a dose dependent relationship between viral load and estimated recombination rate $\hat{\rho}$. (A)** Recombination rate estimates, $\hat{\rho}$, are plotted as a bootstrapped distribution (1000 bootstraps) for groups with varying viral load thresholds separating them. Each box represents the quartiles of the $\hat{\rho}$ distribution and the median is indicated by the central line. Estimates differ between groups at increasing viral load thresholds. **(B)** The number of LDMs available for estimation in each group is indicated by the height of its corresponding bar.

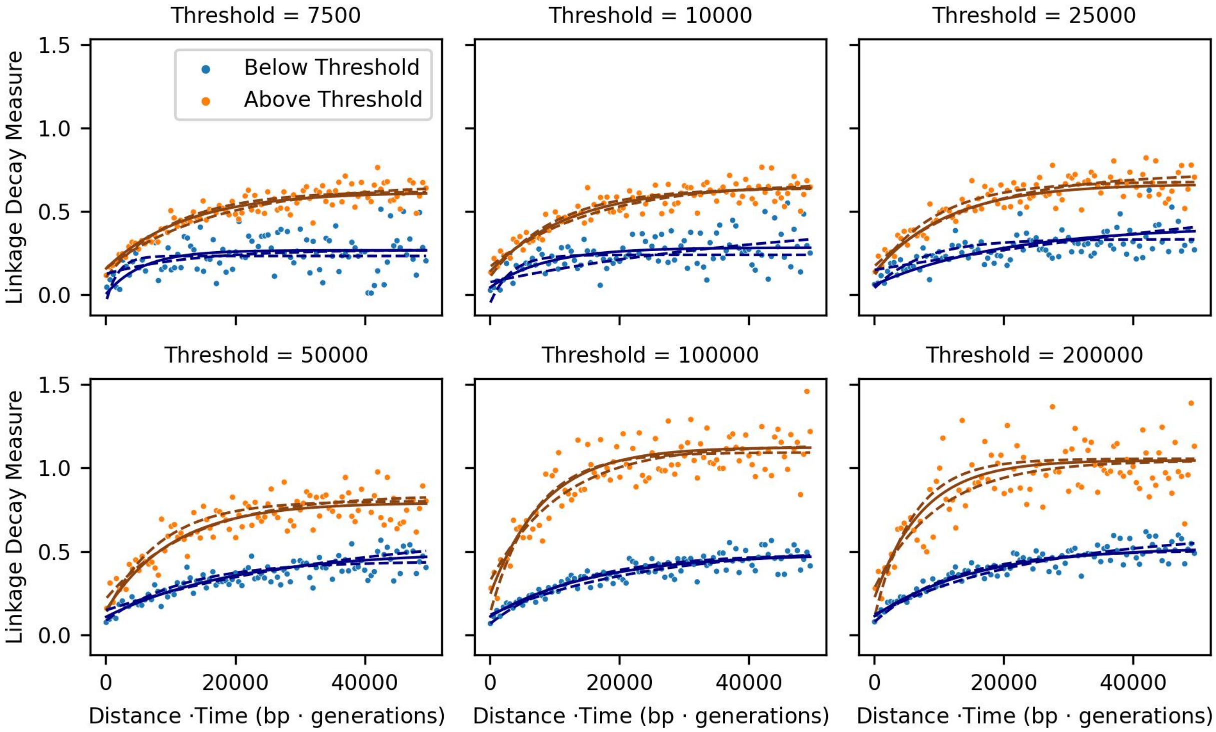

Figure S14: **Trends in mean linkage decay measure (LDM) show that linkage decays more rapidly as the viral load threshold (copies/mL) separating low and high viral load groups increases.** Each panel contains the linkage decay curves, with binned averages of the LDMs represented by dots, alongside several fits to the data (moving average for display purposes only, window size = 500 bp · generations). The fit producing the median estimate for the bootstrapped distribution (1000 bootstraps) is indicated by a solid line while the fits that produced the estimates at the 0.025 and 0.975 quantiles of the bootstrapped distribution are represented by dashed lines. Panels are separated by the viral load threshold dividing the two groups.

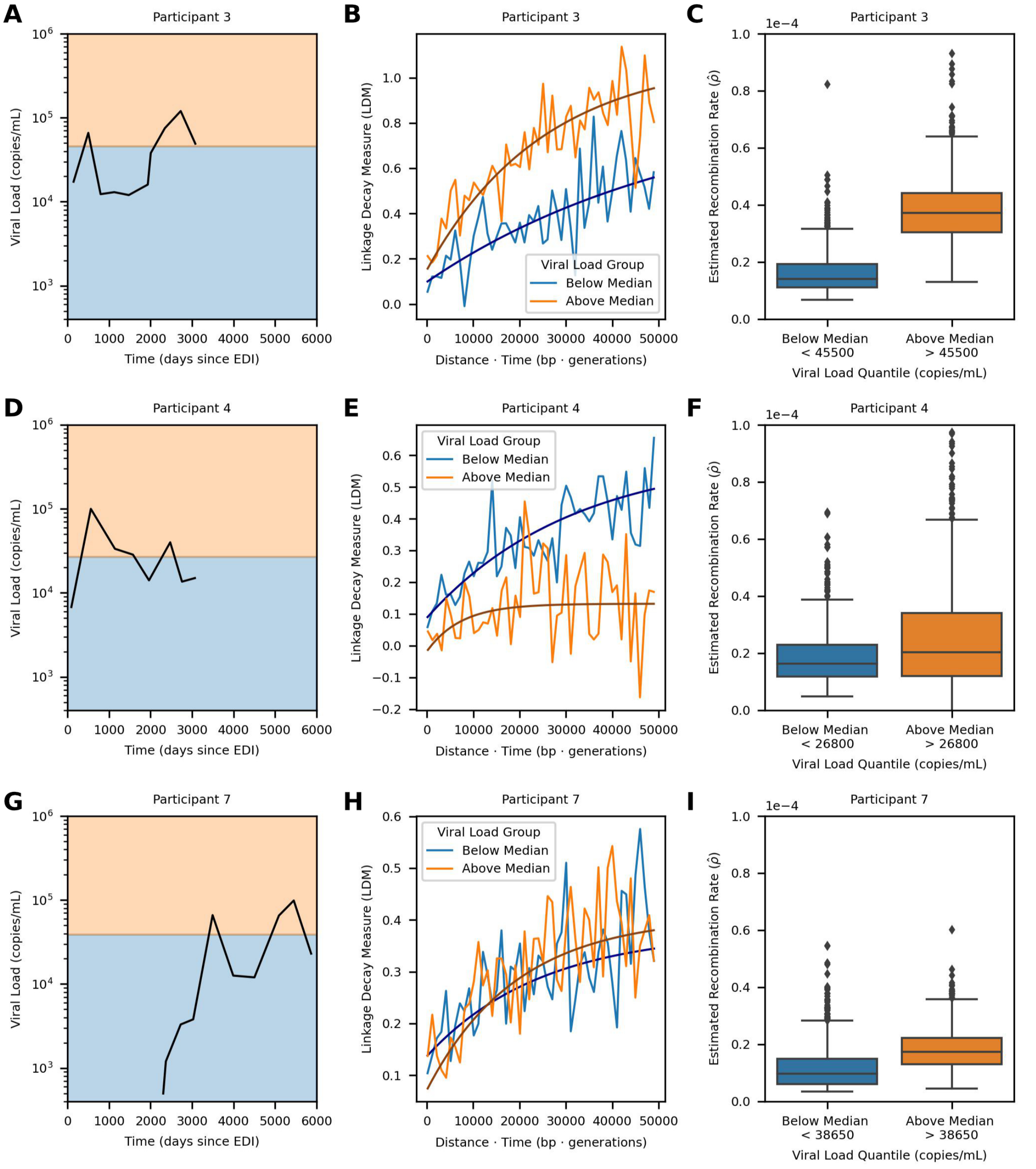

Figure S15: **Recombination rate estimation within individuals p3, p4, and p7.** Each row shows the results of a RATS-LD analysis run on only data from Participant 3 (top), 4 (middle), or 7 (bottom). The first plot in the row **(A, D, G)** shows the viral load trajectory for the given individual, with background shading indicating the two groups (above median in orange and below median in blue) that pairs of time points were divided into based on their average viral load. **(B, E, H)** RATS-LD curve fits representing the median bootstrapped estimate for each group are plotted on top of the binned average of the LDMs from the given participant's data (moving average used for display purposes only, window size = 1000 bp $\cdot$ generations). **(C, F, I)** Box and whisker plots, with each box representing the quartiles of the $\hat{\rho}$ distribution, show the bootstrapped distributions (1000 bootstraps) of recombination rate estimates, $\hat{\rho}$, for each individual.

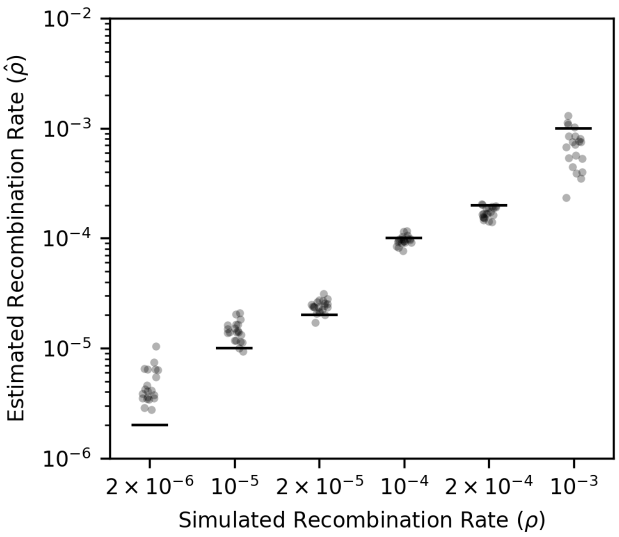

Figure S16: **Recombination rate can also be estimated by using the D statistic to measure linkage in simulated data.** Each dot represents the median of a bootstrapped estimate distribution (1000 bootstraps). Recombination rate estimates are from RATS-LD runs where the D statistic ($D = p_{12} - p_1 p_2$ where p_1 and p_2 are the frequencies of the the majority alleles at the corresponding loci and p_{12} is the haplotype frequency composed of the majority alleles) was used to measure linkage decay rather than the normalized version, D'.

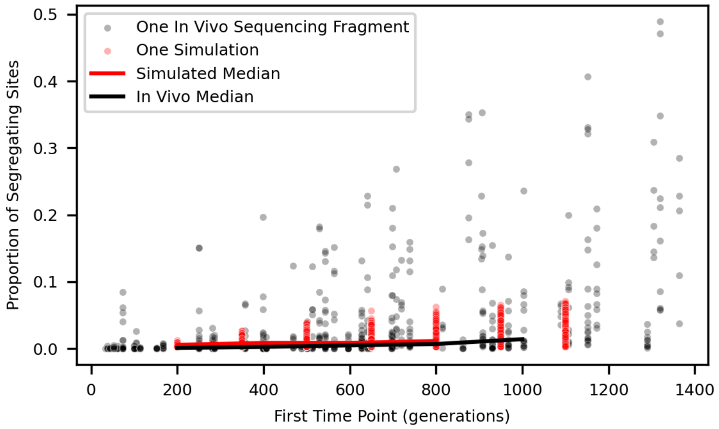

Figure S17: **Selection simulations match the proportion of segregating sites observed *in vivo*.** The number of segregating sites in simulated and *in vivo* [58] data sets is shown here after data filtering and LDM calculations (See Materials & Methods for details). For each time point pair in these data sets, the proportion of segregating sites (# of sites/sequence length) was plotted against the first time point in the pair. Each dot represents the result for one time point pair in either a simulation (red) or an *in vivo* sequencing fragment (black). Medians were calculated and displayed using 5 bins (bin width = 200 generations).

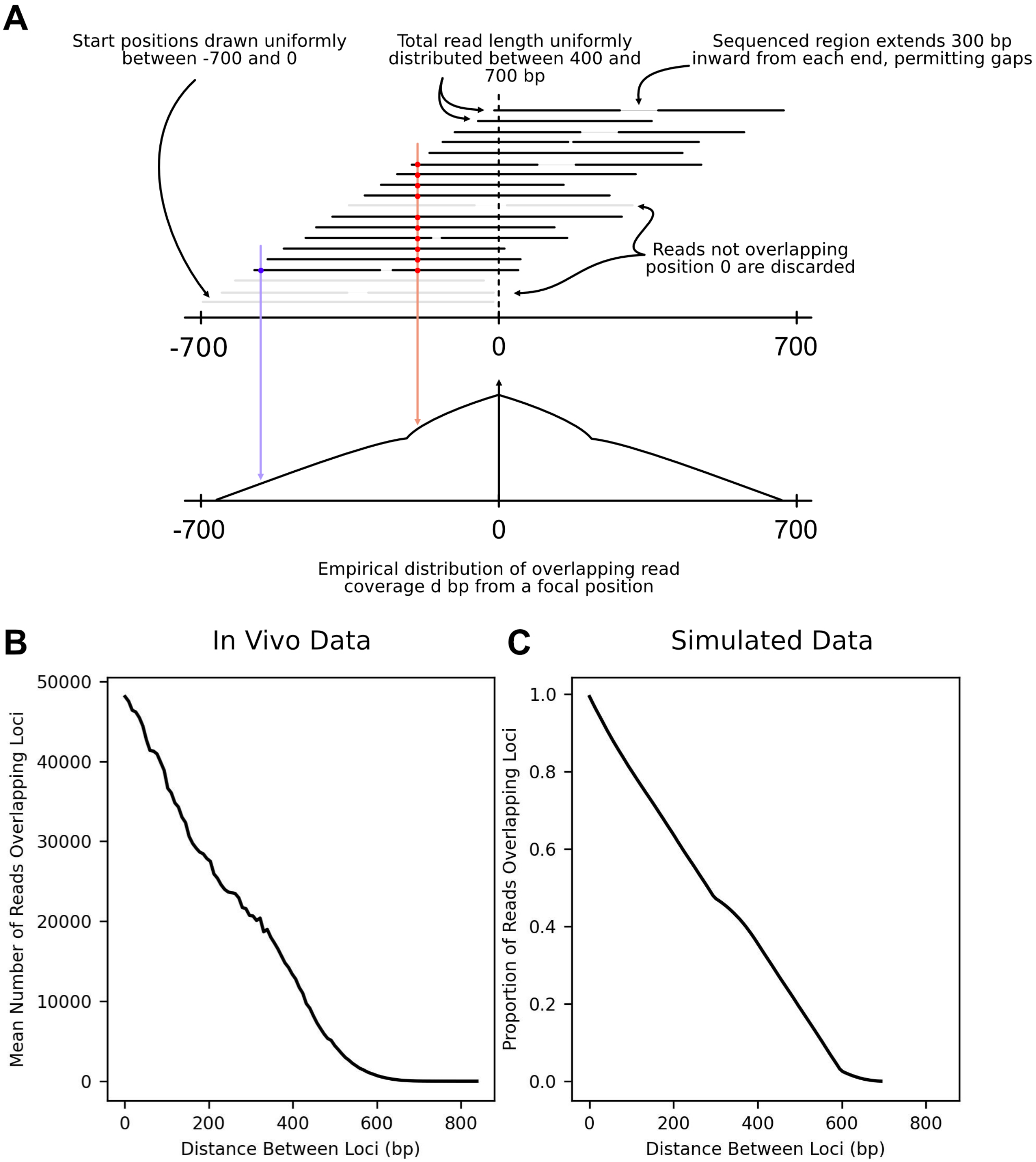

Figure S18: **An empirical distribution of read coverage is used to mimic the *in vivo* read coverage simulated data sets (A)**. Schematic of how the empirical distribution of read coverage is formed. **(B)** Moving average of the number of reads observed to be overlapping pairs of loci d distance apart in the *in vivo* data set (moving average window size = 8bp). **(C)** Moving average of the proportion of reads overlapping loci in the empirical distribution formed via the sampling scheme described in **(A)** (moving average window size = 8bp).
