# Supplementary figures and images for: Elevated HIV Viral Load is Associated with Higher Recombination Rate In Vivo
Source: Mol Biol Evol. 2024 Jan 10;41(1):msad260. doi: 10.1093/molbev/msad260 (PMC10777272; doi:10.1093/molbev/msad260)

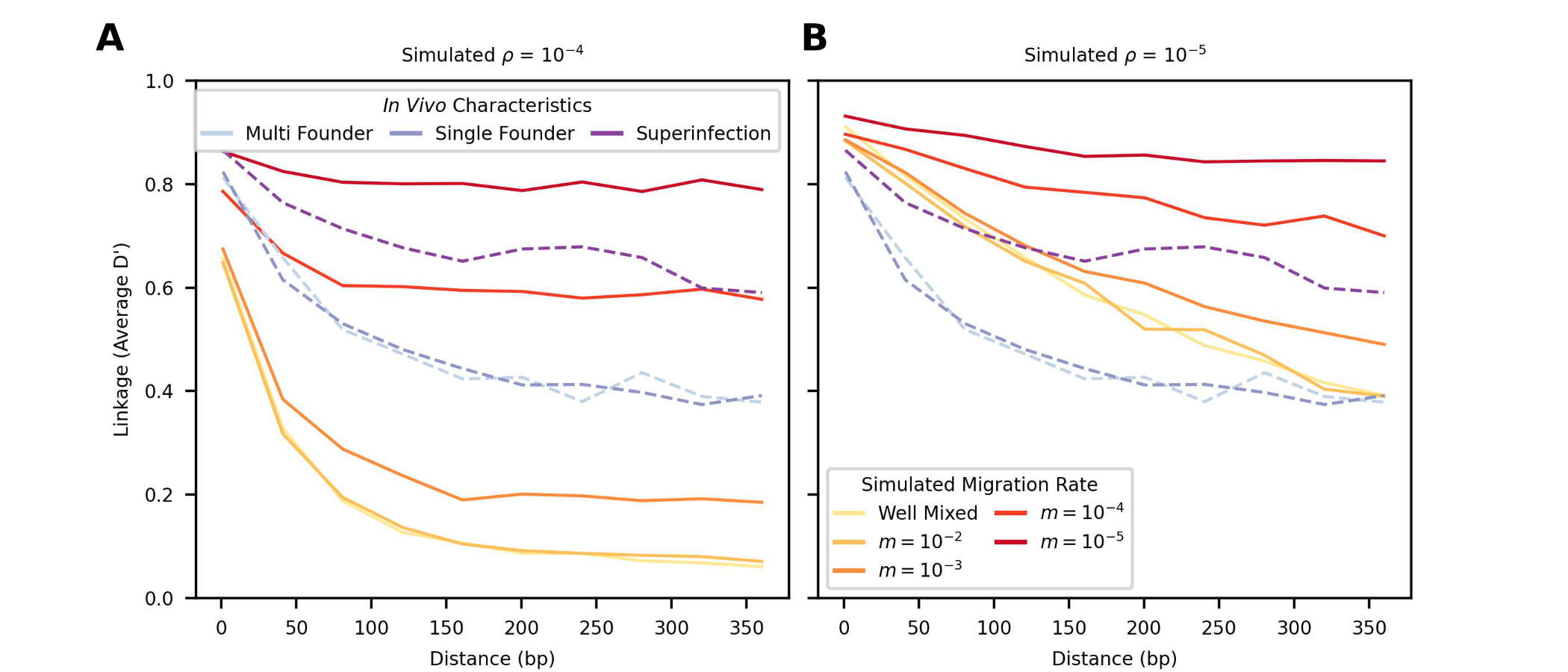

Supplement: msad260_Supplementary_Data [file msad260_supplementary_data.zip › linkage_comparison.jpg]

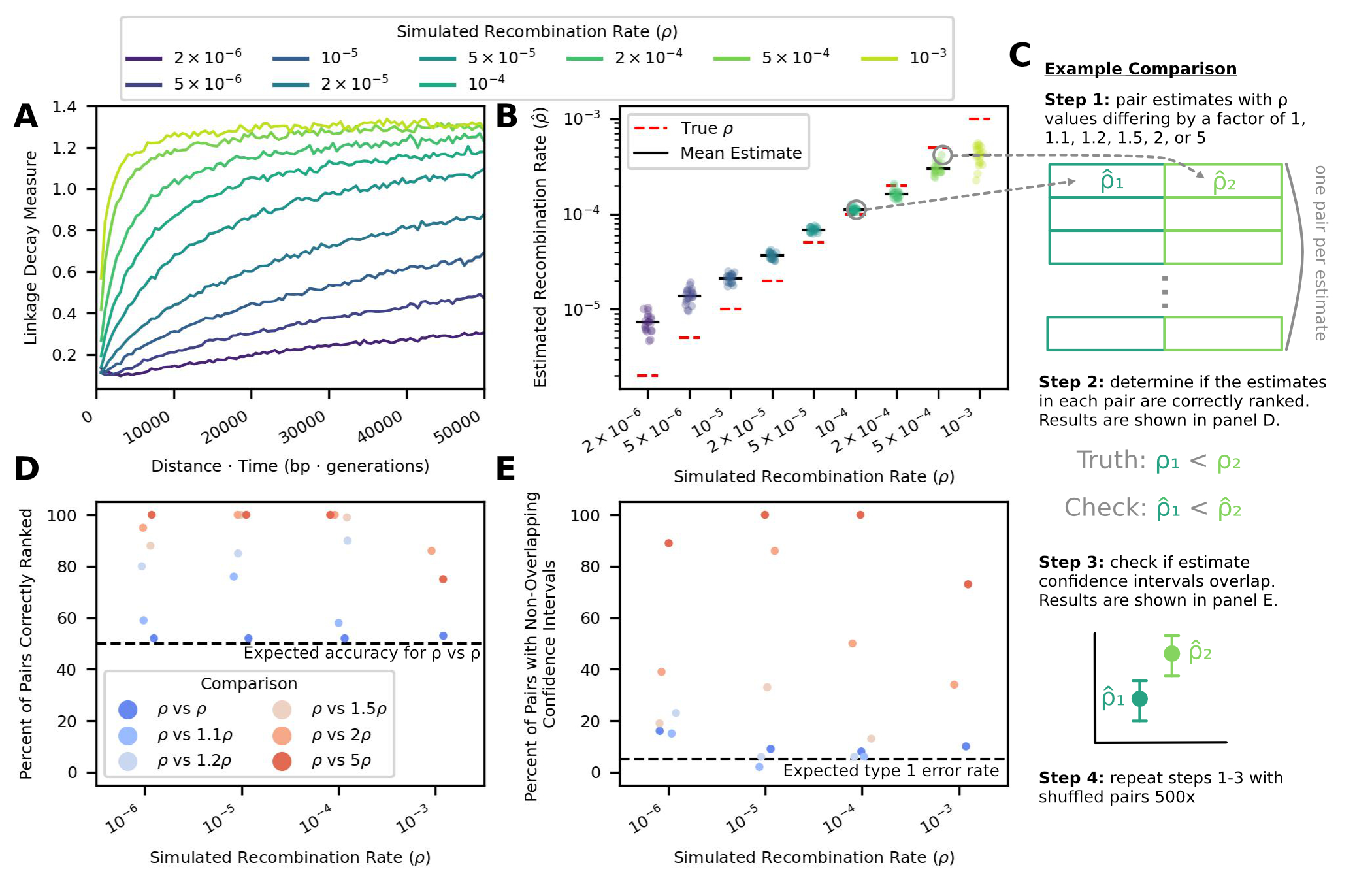

Supplement: msad260_Supplementary_Data [file msad260_supplementary_data.zip › neutral_accuracy.jpg]

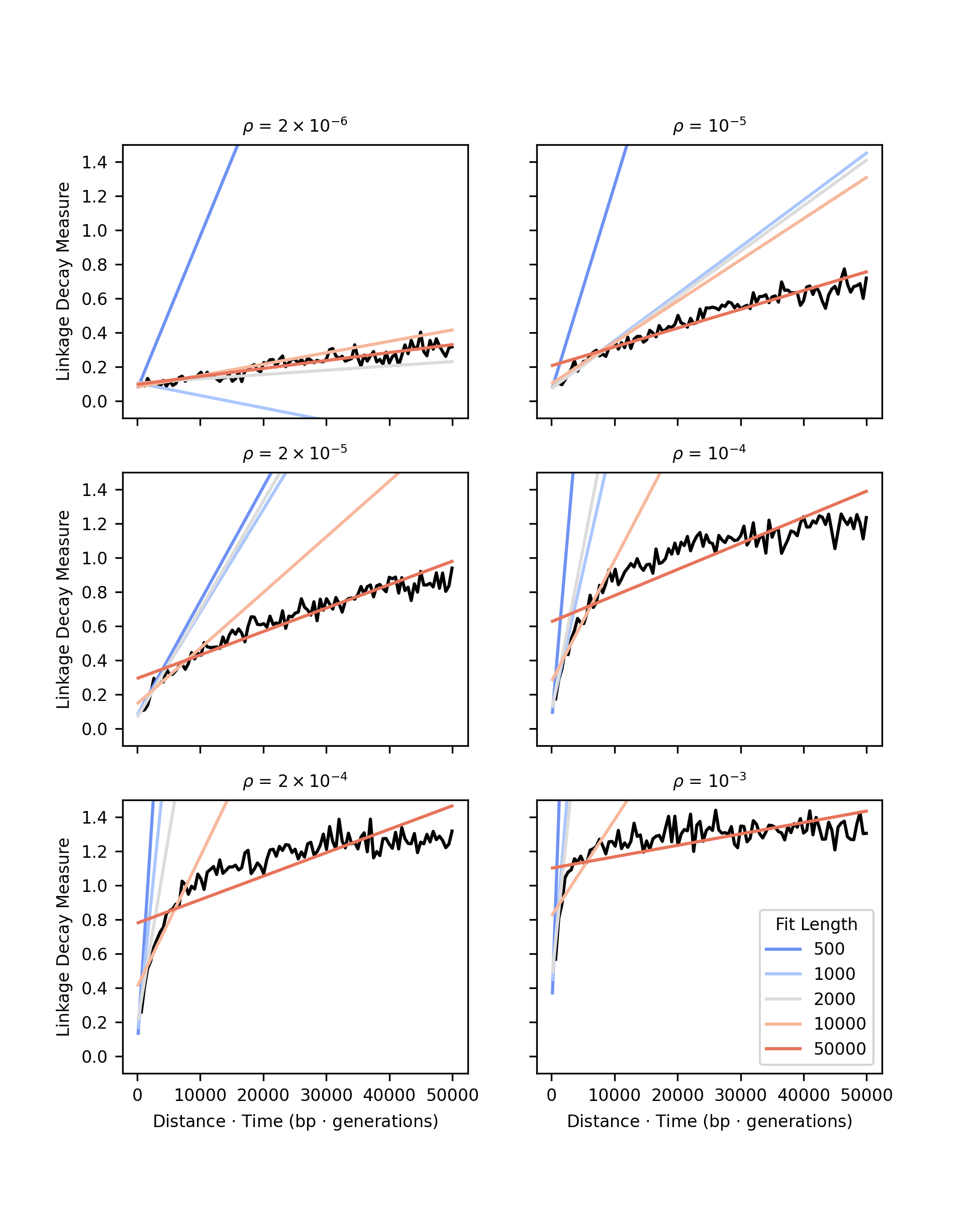

Supplement: msad260_Supplementary_Data [file msad260_supplementary_data.zip › resampled_fits.png]

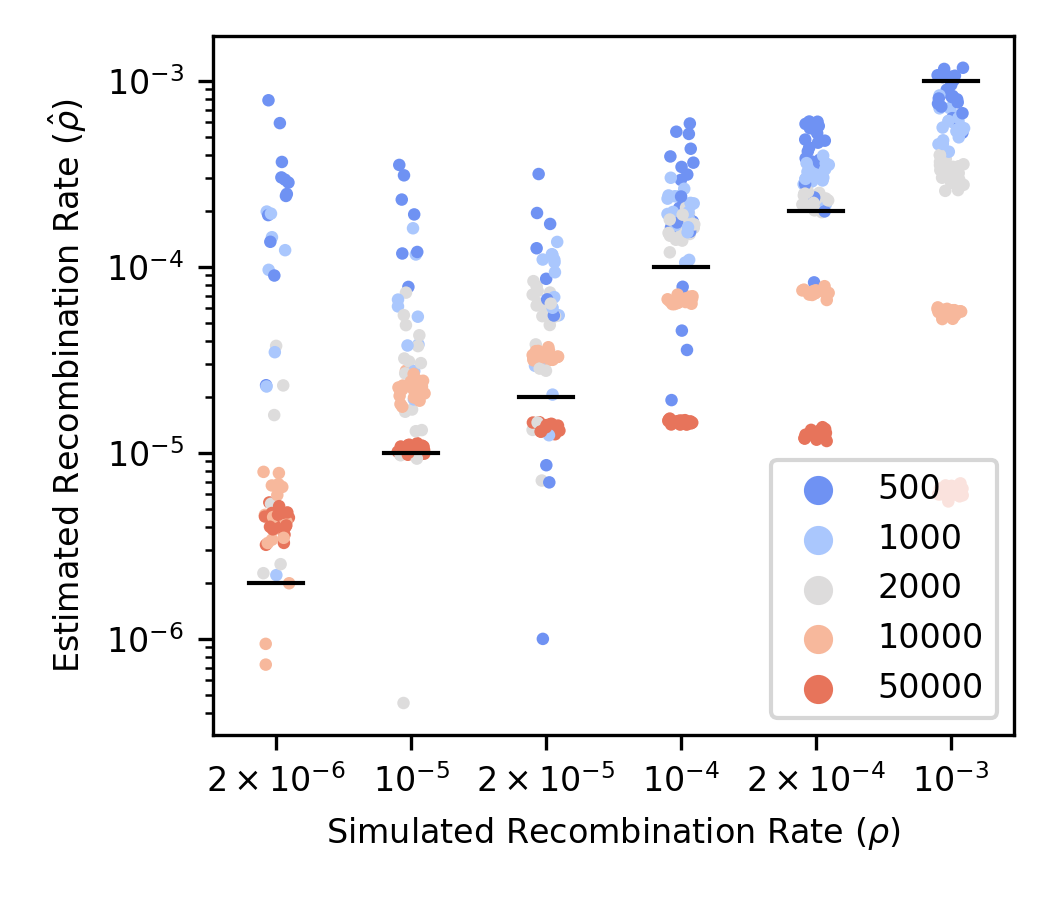

Supplement: msad260_Supplementary_Data [file msad260_supplementary_data.zip › resampled_line_fits_both_20.png]

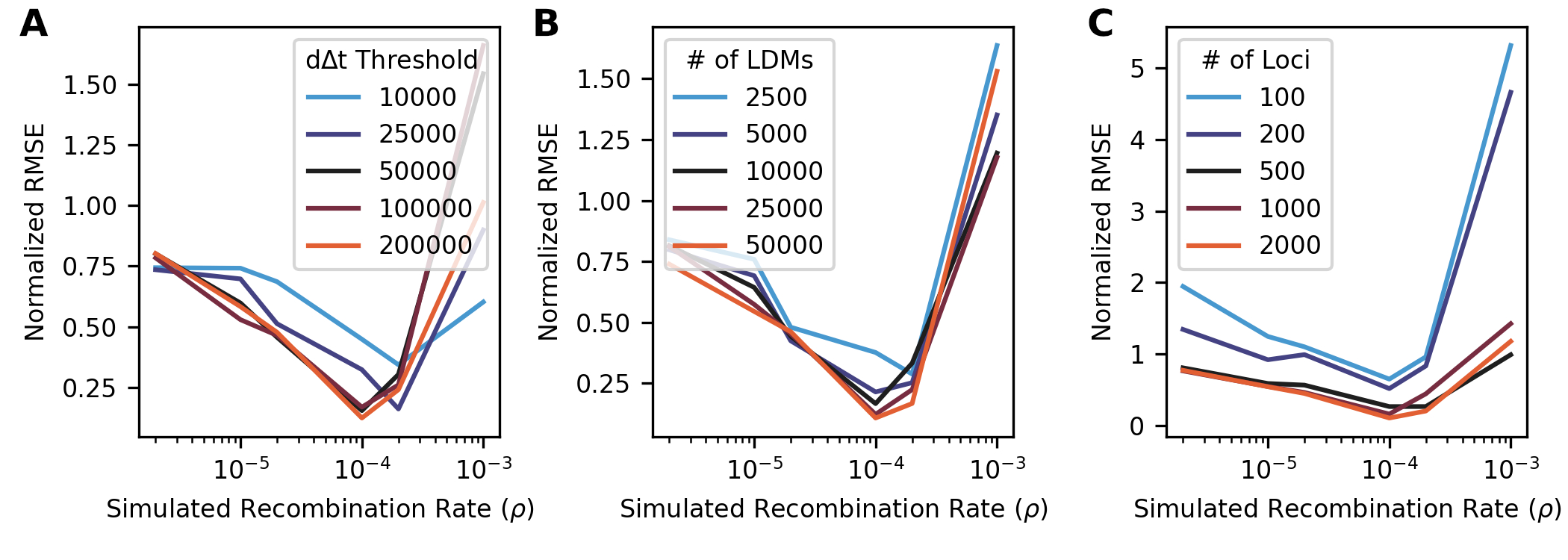

Supplement: msad260_Supplementary_Data [file msad260_supplementary_data.zip › rmse_neutral.jpg]

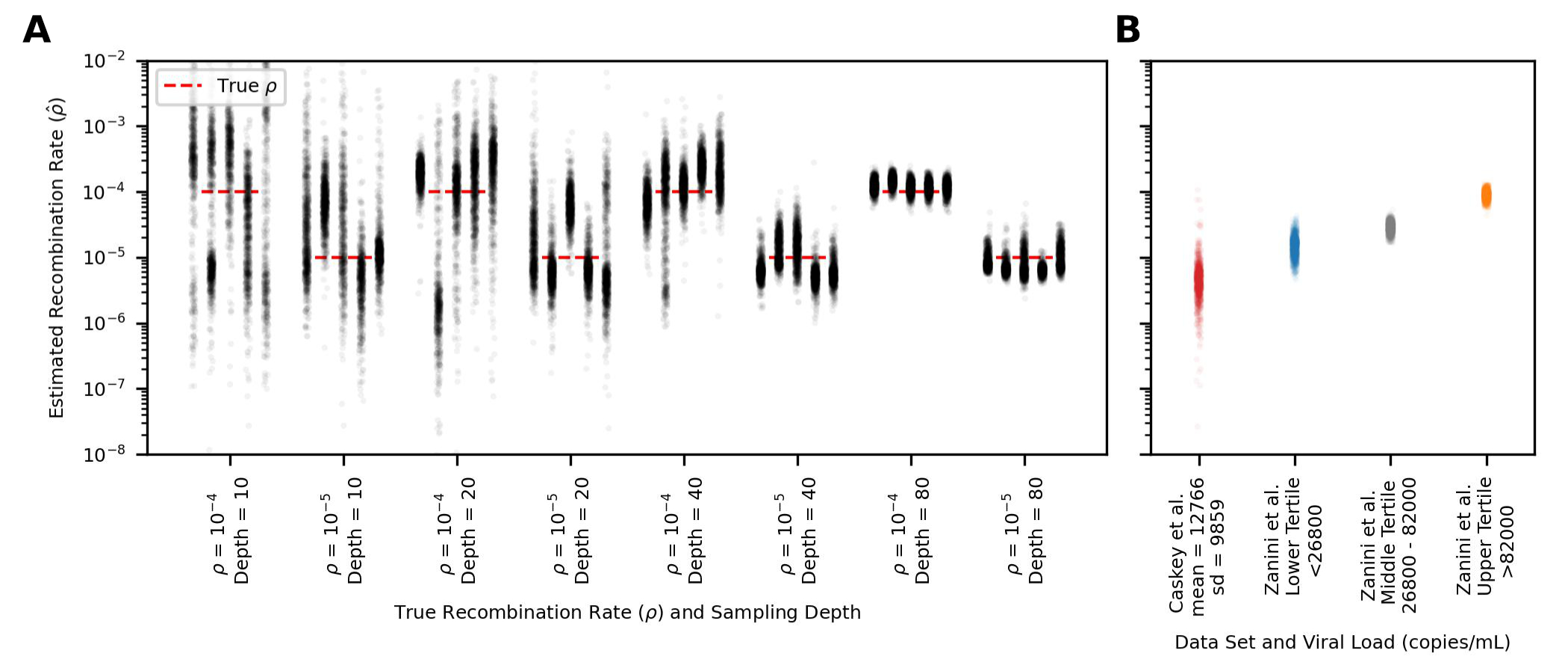

Supplement: msad260_Supplementary_Data [file msad260_supplementary_data.zip › sampling_depth.jpg]

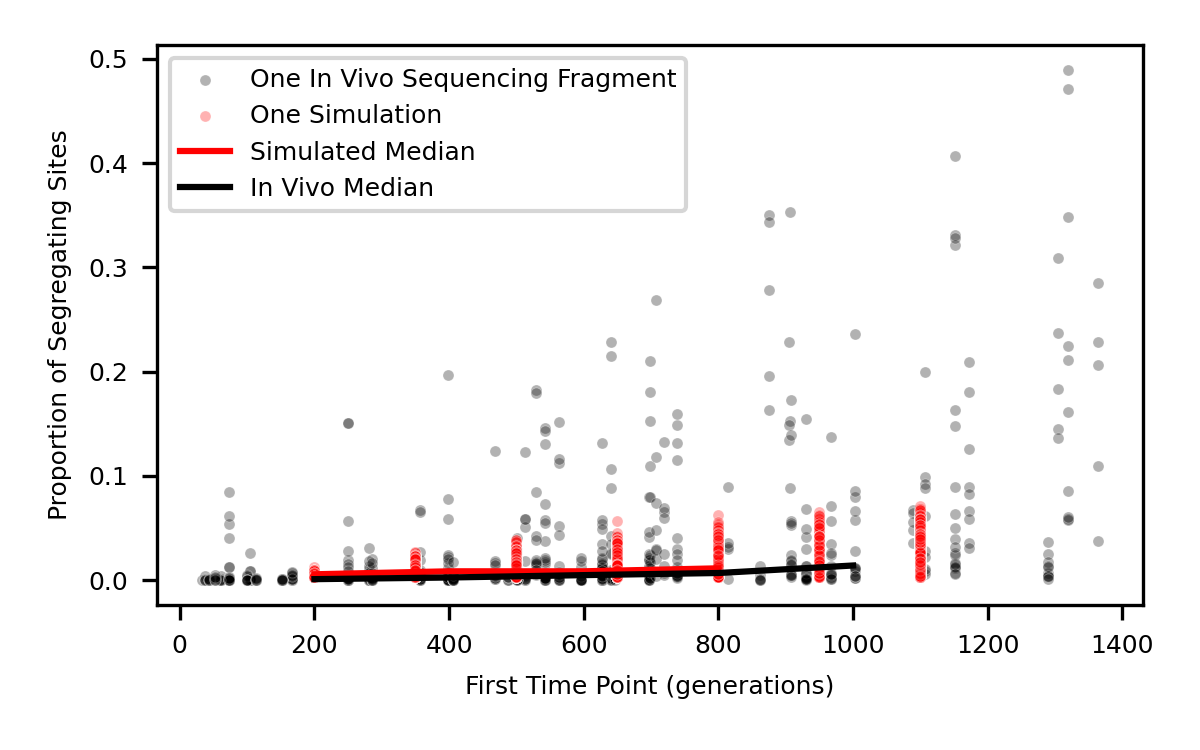

Supplement: msad260_Supplementary_Data [file msad260_supplementary_data.zip › segregating_sites_median.png]

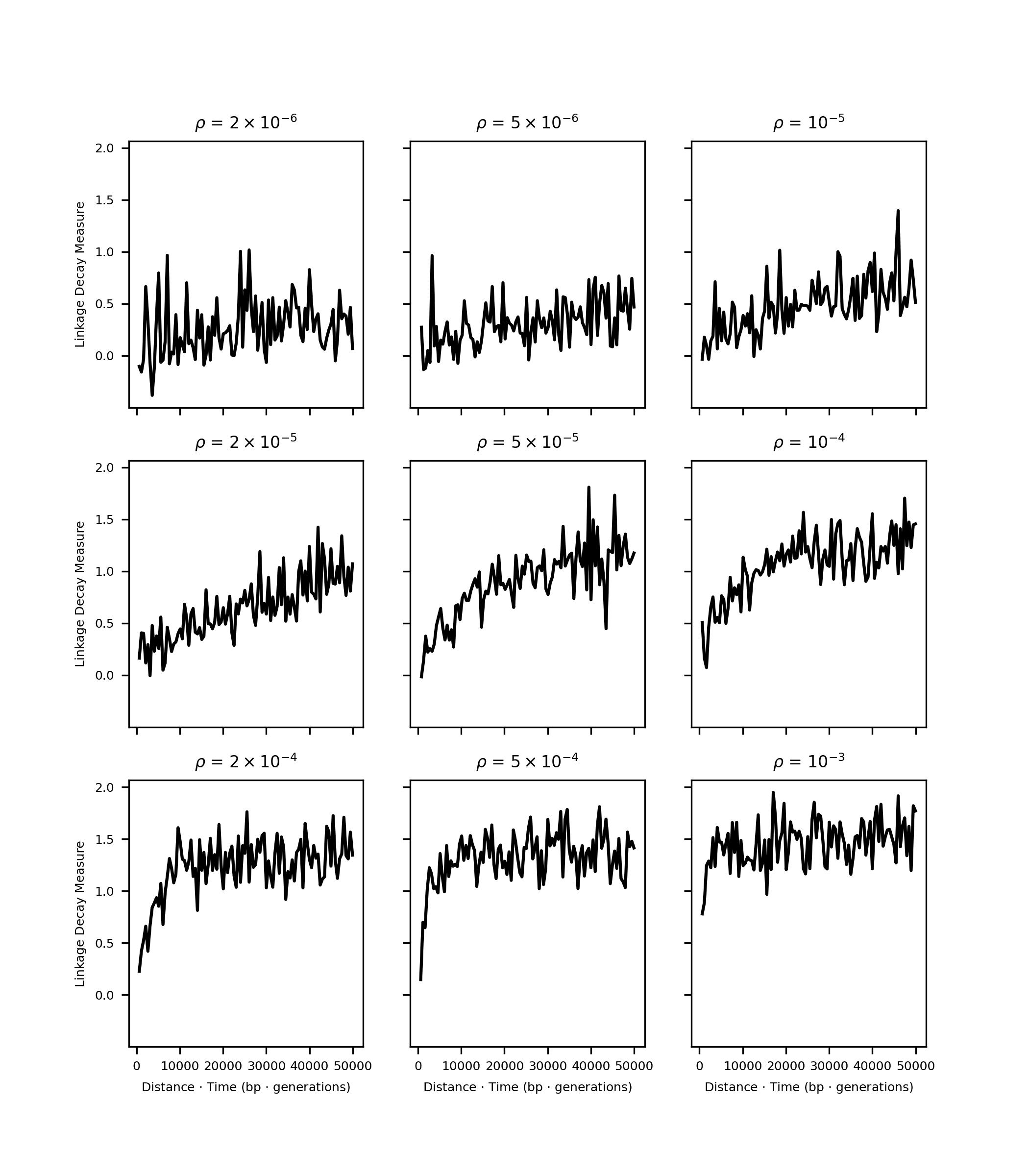

Supplement: msad260_Supplementary_Data [file msad260_supplementary_data.zip › sel_curves.jpg]

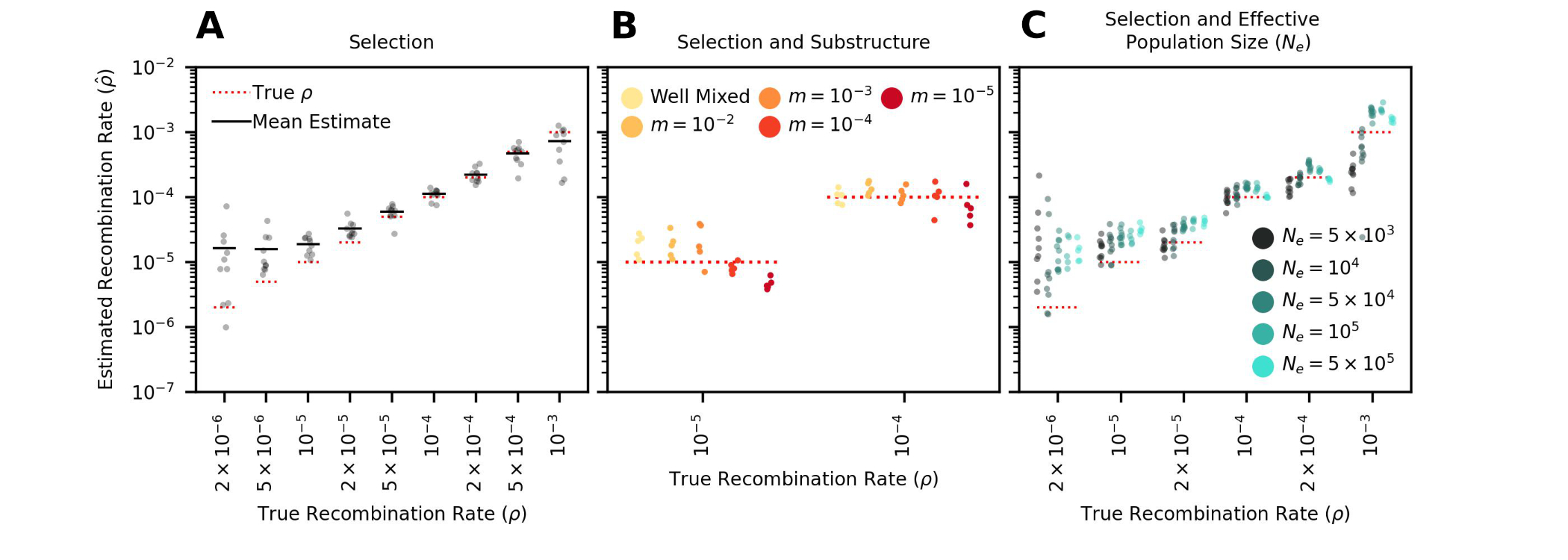

Supplement: msad260_Supplementary_Data [file msad260_supplementary_data.zip › selection_accuracy.jpg]

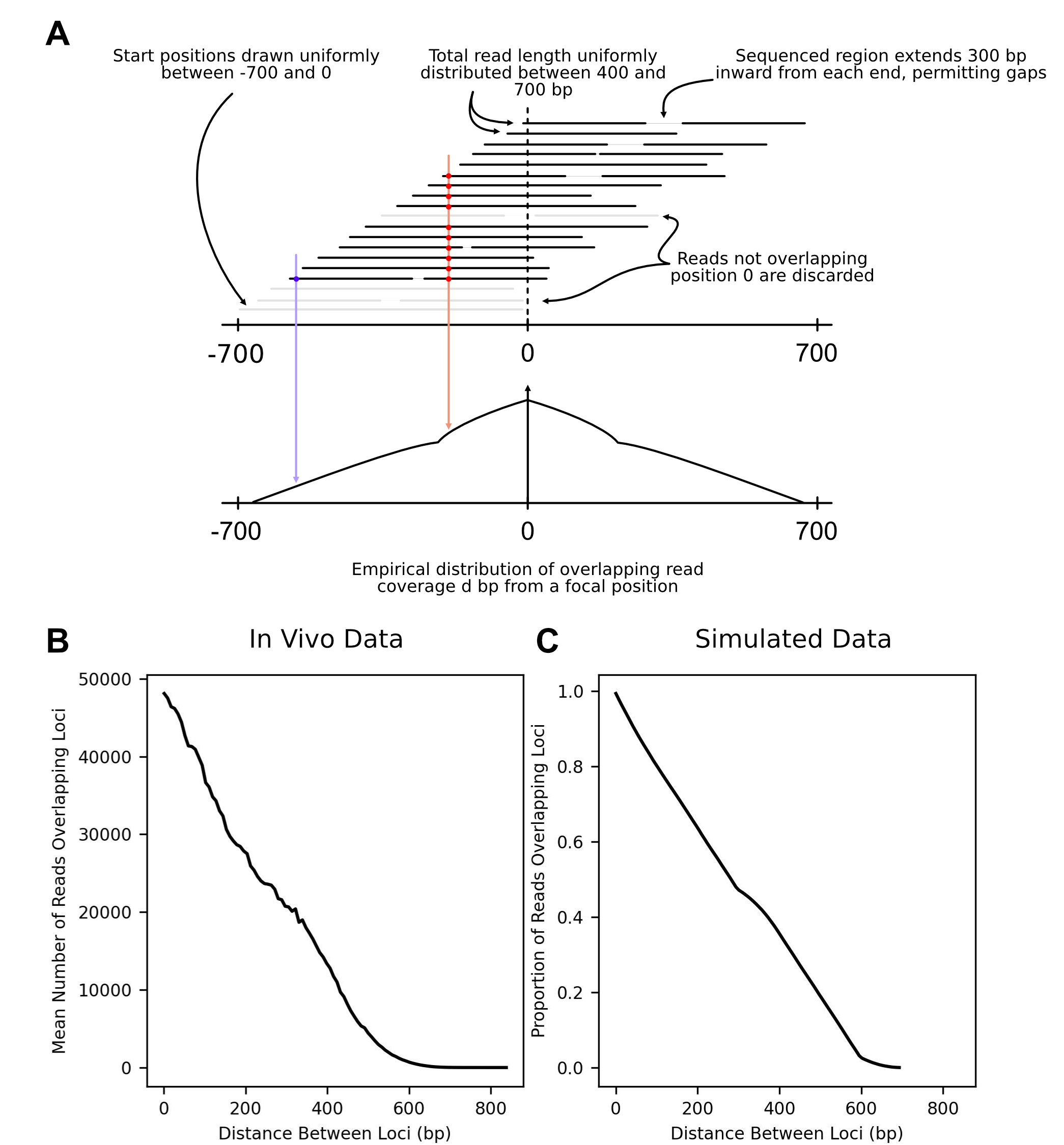

Supplement: msad260_Supplementary_Data [file msad260_supplementary_data.zip › supp_coverage.jpg]

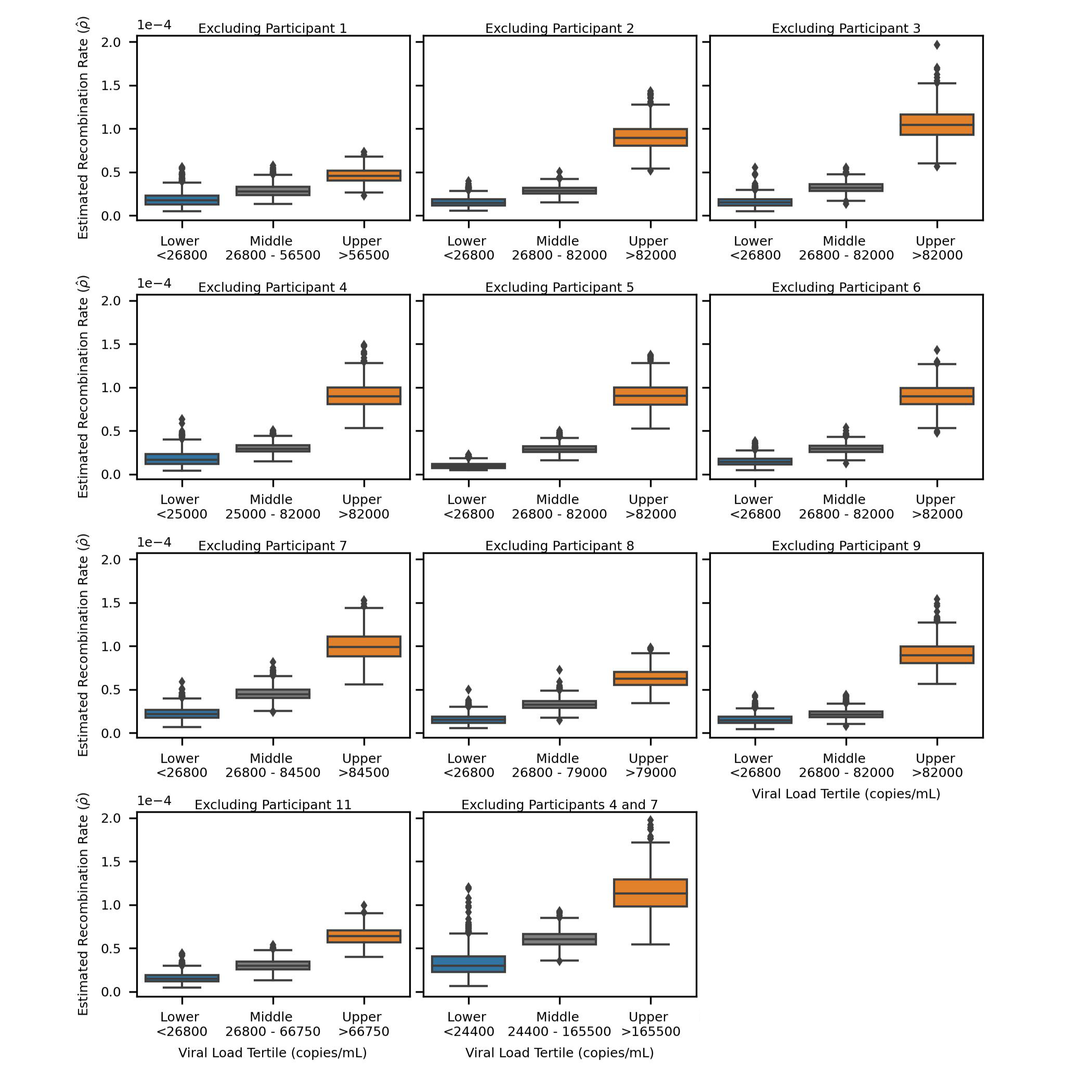

Supplement: msad260_Supplementary_Data [file msad260_supplementary_data.zip › supp_loo.jpg]

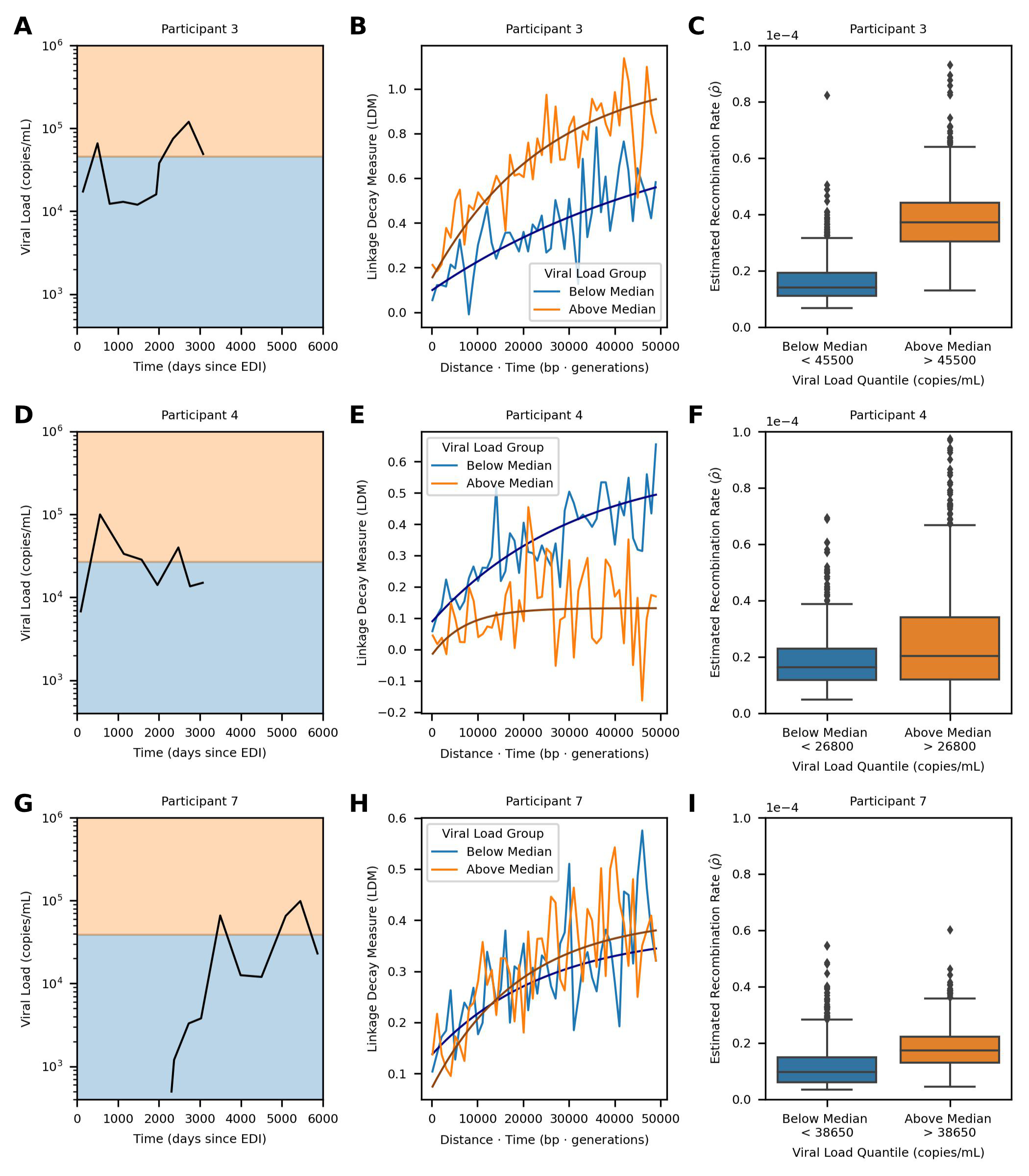

Supplement: msad260_Supplementary_Data [file msad260_supplementary_data.zip › supp_participant.jpg]

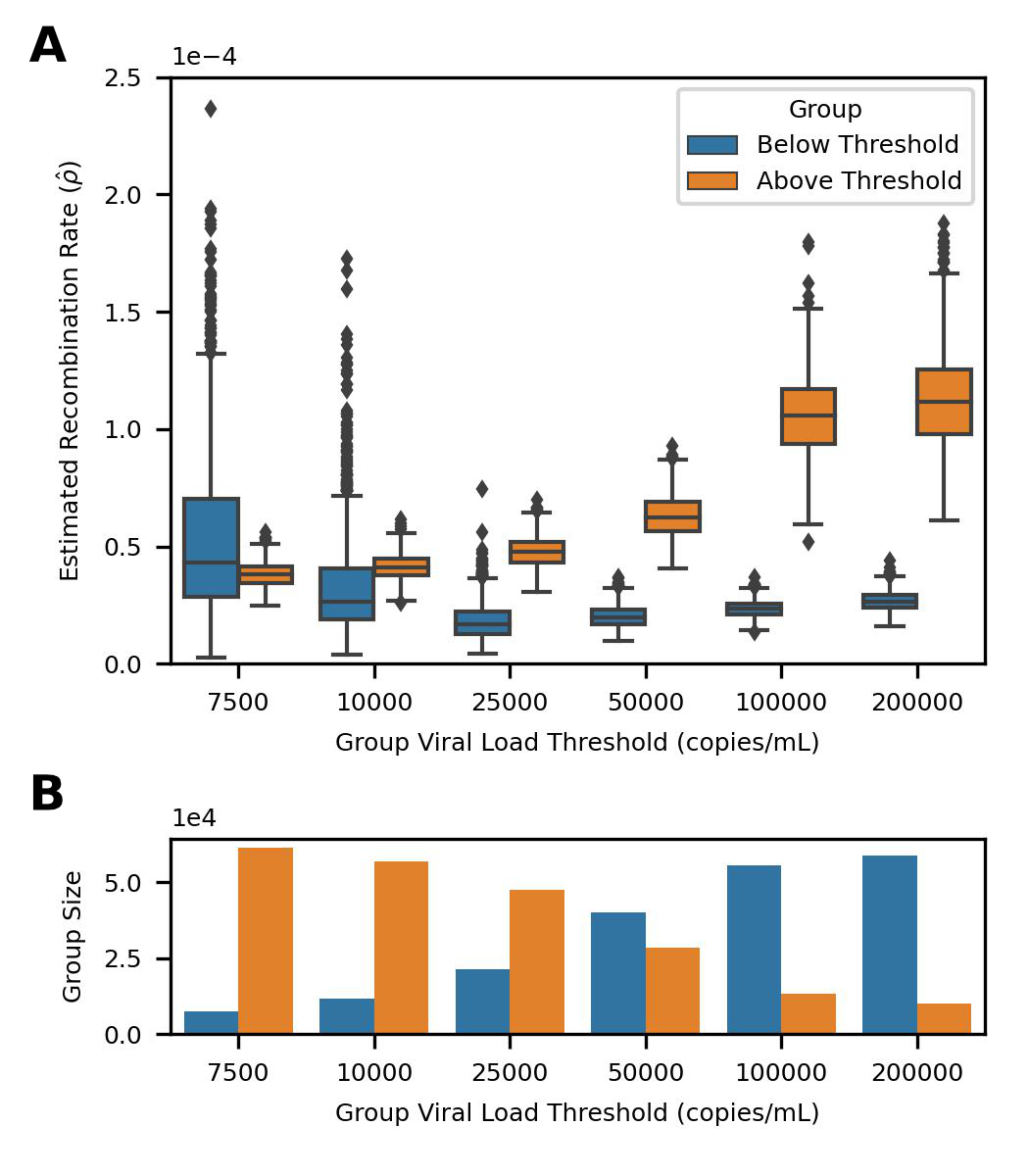

Supplement: msad260_Supplementary_Data [file msad260_supplementary_data.zip › supp_thresh.jpg]

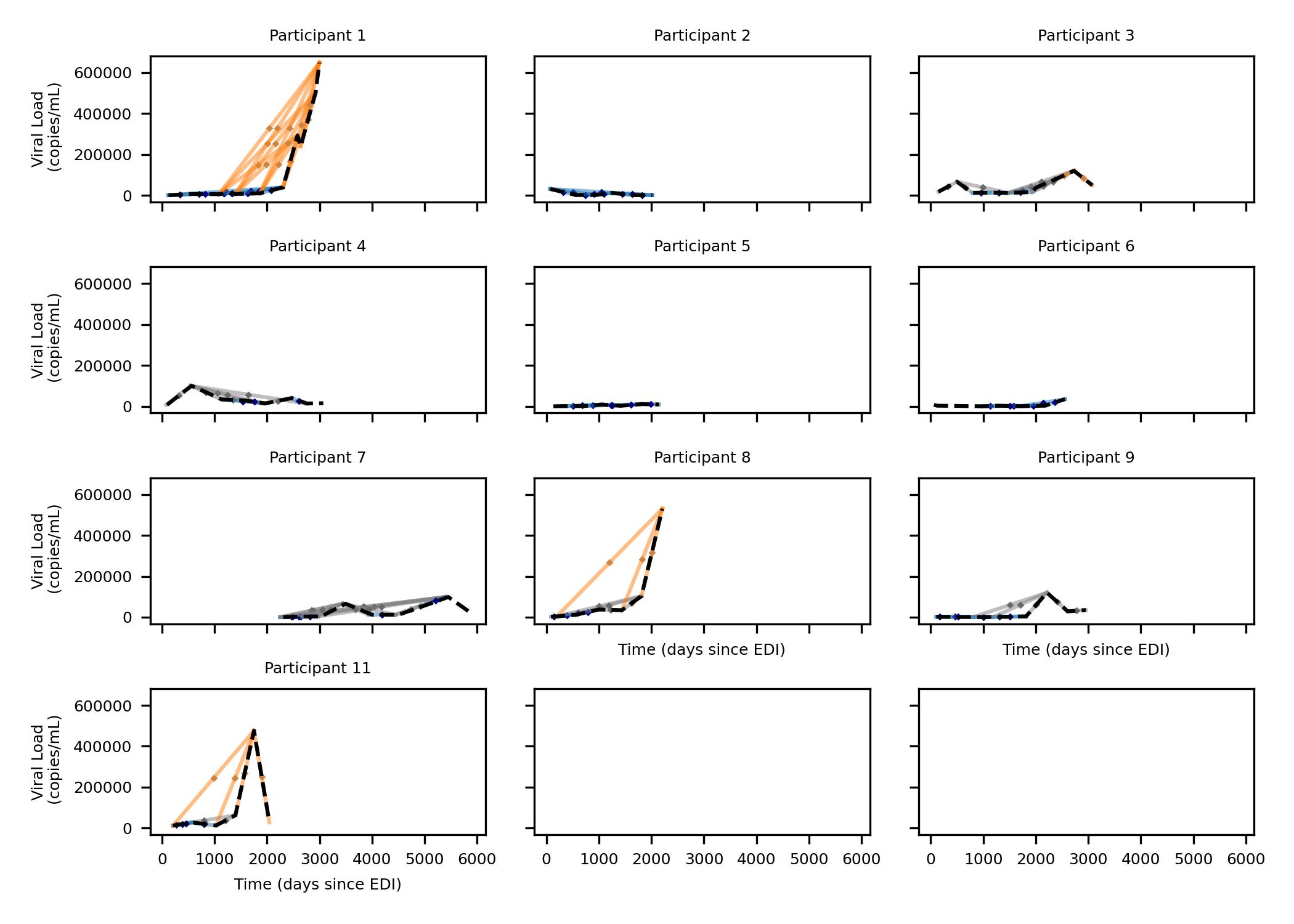

Supplement: msad260_Supplementary_Data [file msad260_supplementary_data.zip › supp_vl.jpg]

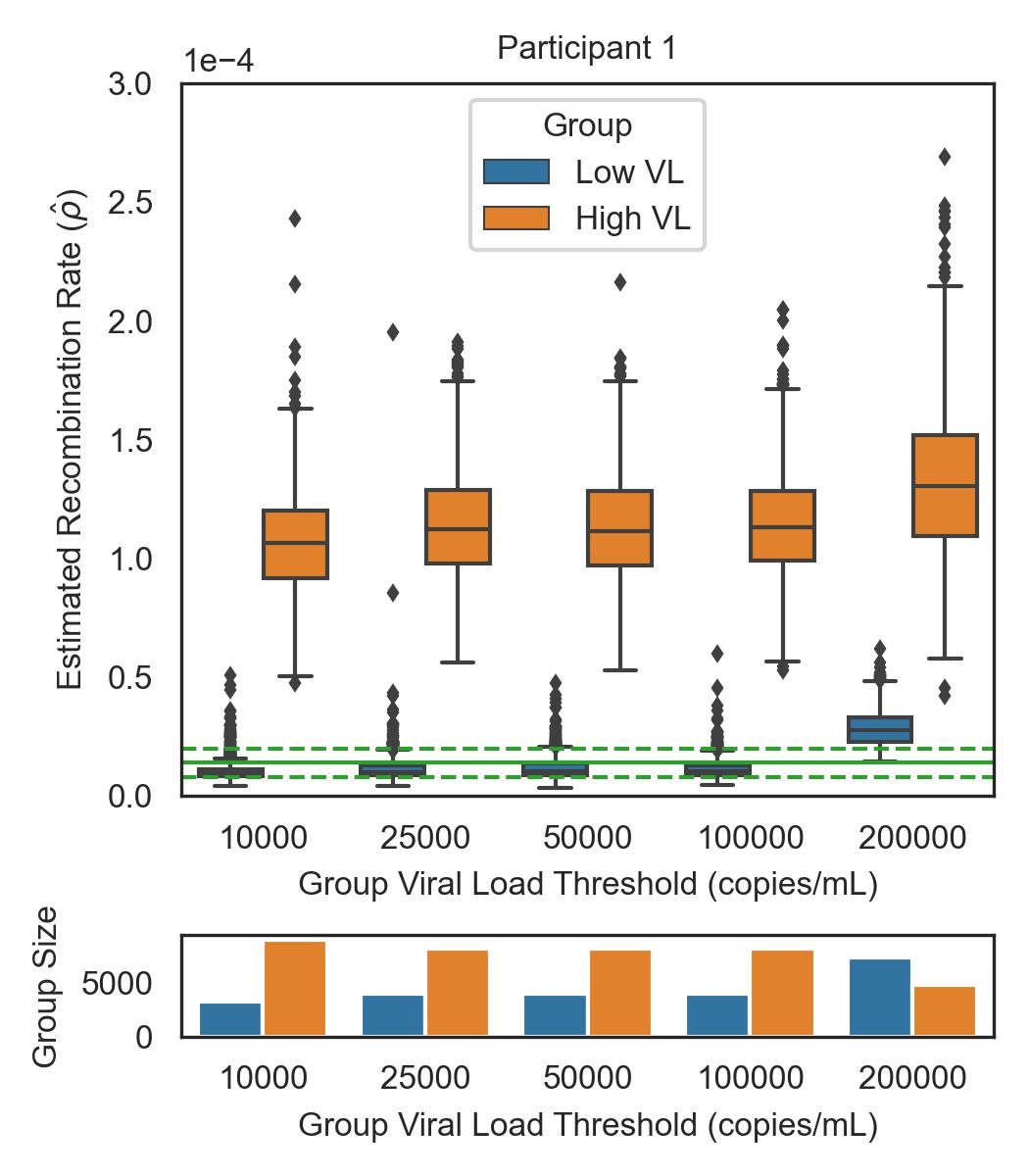

Supplement: msad260_Supplementary_Data [file msad260_supplementary_data.zip › supp_within_1000_p1.jpg]

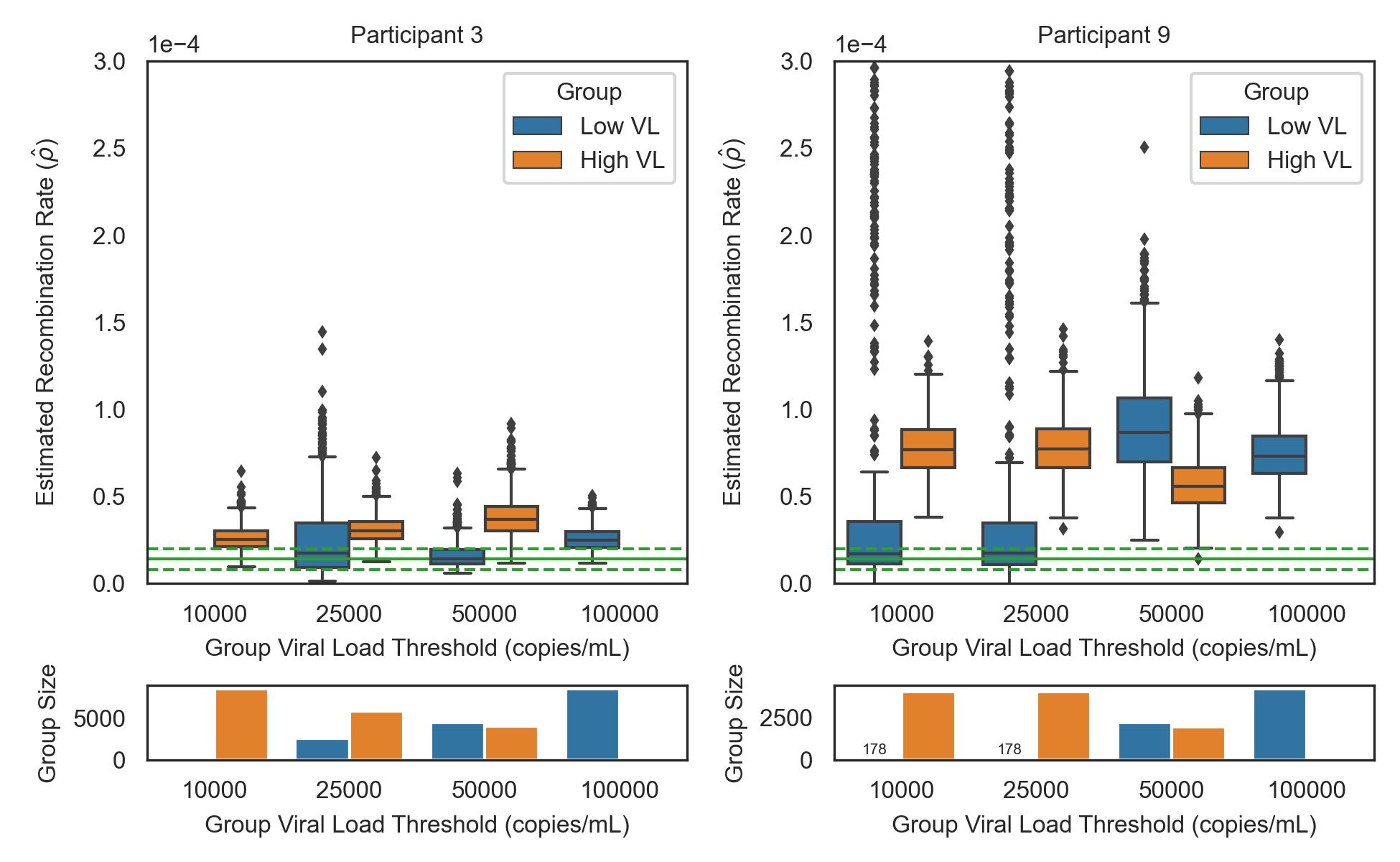

Supplement: msad260_Supplementary_Data [file msad260_supplementary_data.zip › supp_within1000.jpg]

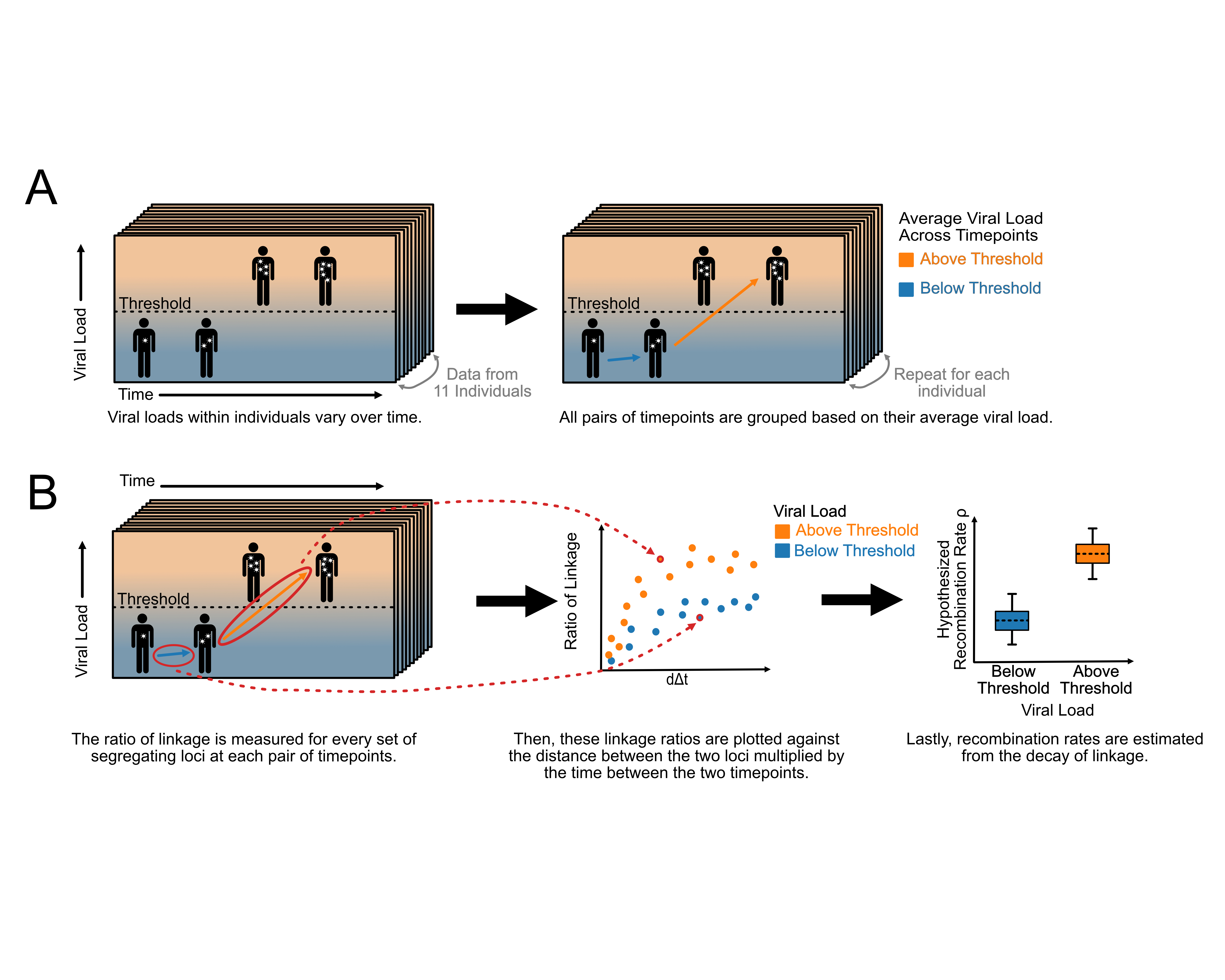

Supplement: msad260_Supplementary_Data [file msad260_supplementary_data.zip › supp_workflow.jpg]

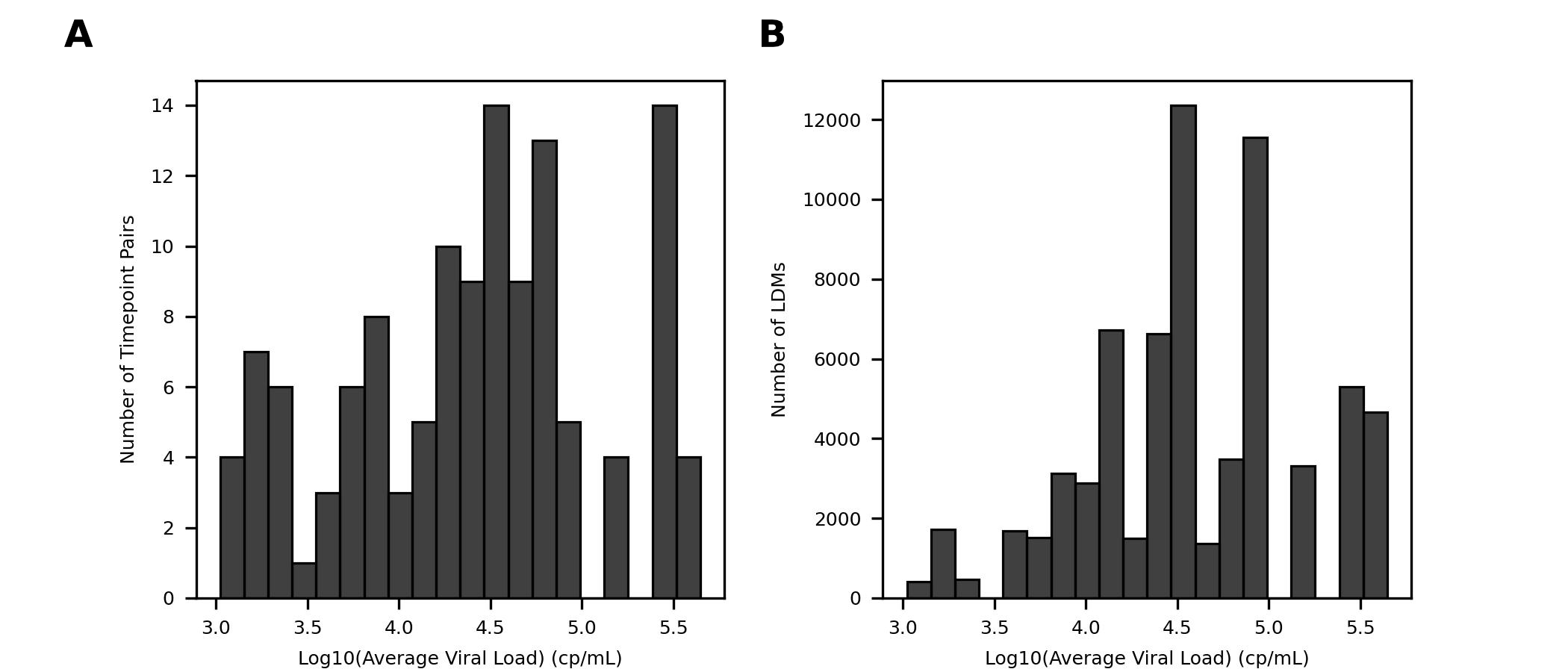

Supplement: msad260_Supplementary_Data [file msad260_supplementary_data.zip › vlHist.jpg]

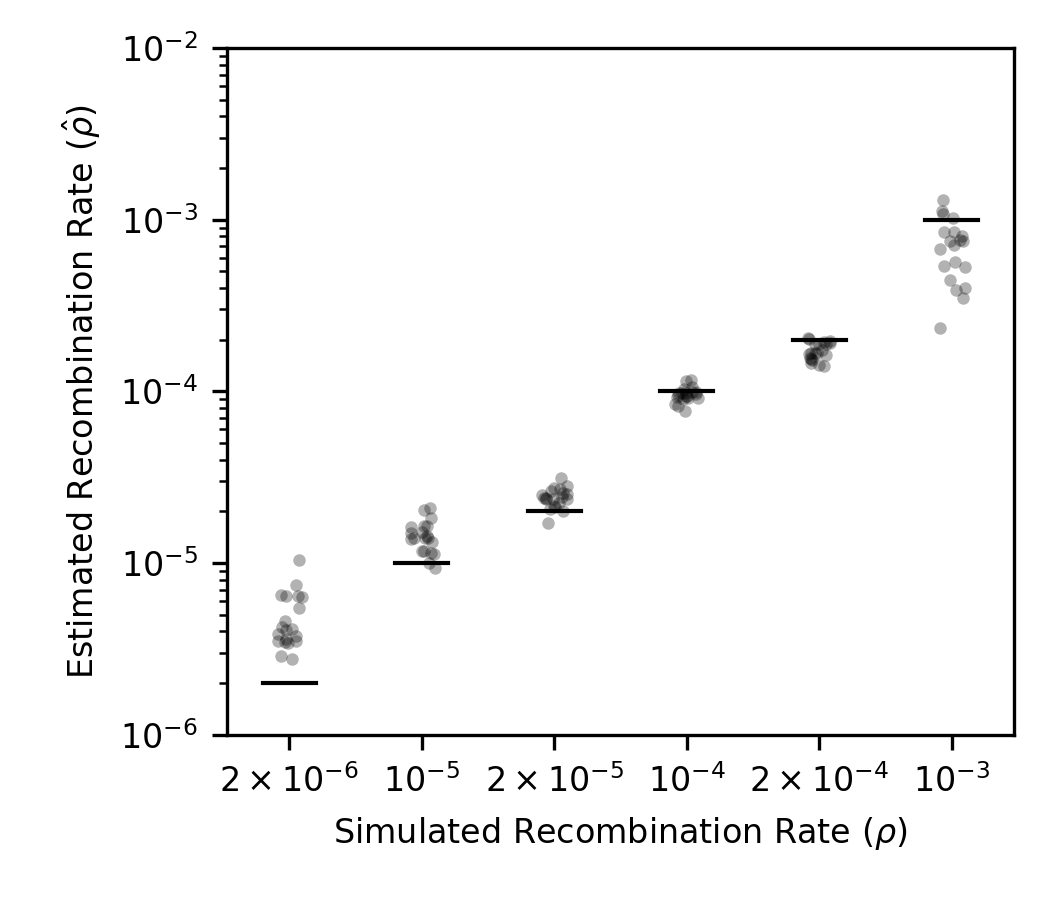

Supplement: msad260_Supplementary_Data [file msad260_supplementary_data.zip › accuracy_D1000.png]

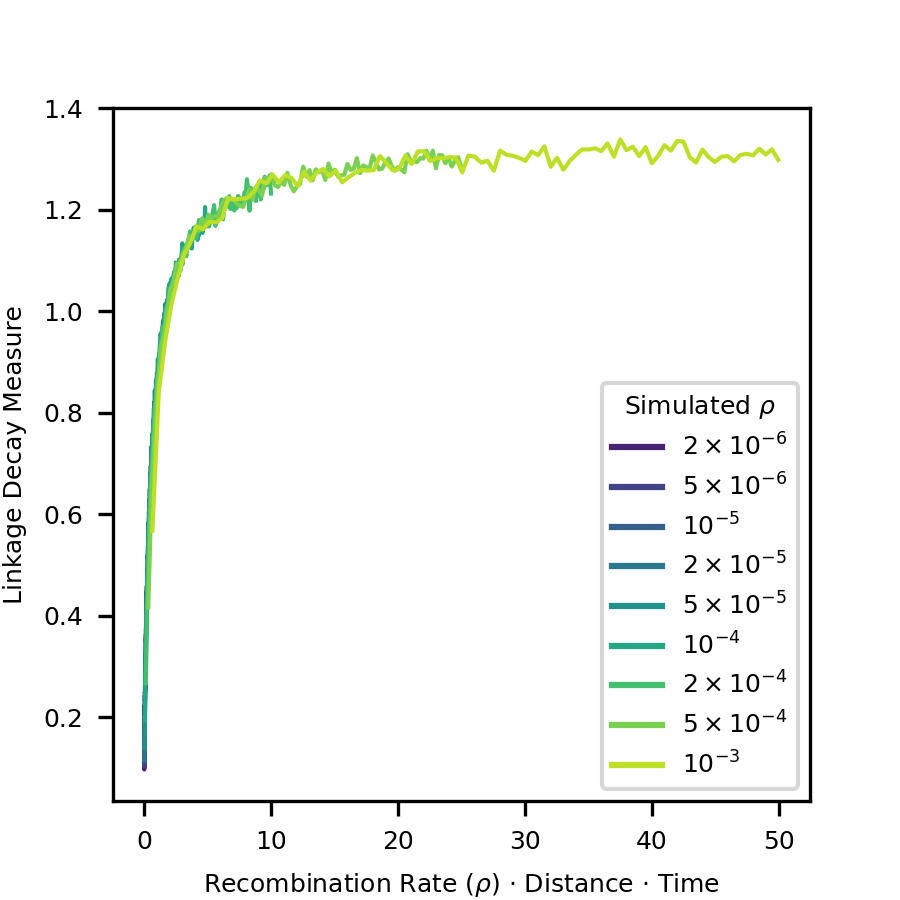

Supplement: msad260_Supplementary_Data [file msad260_supplementary_data.zip › collapsed_curves.png]

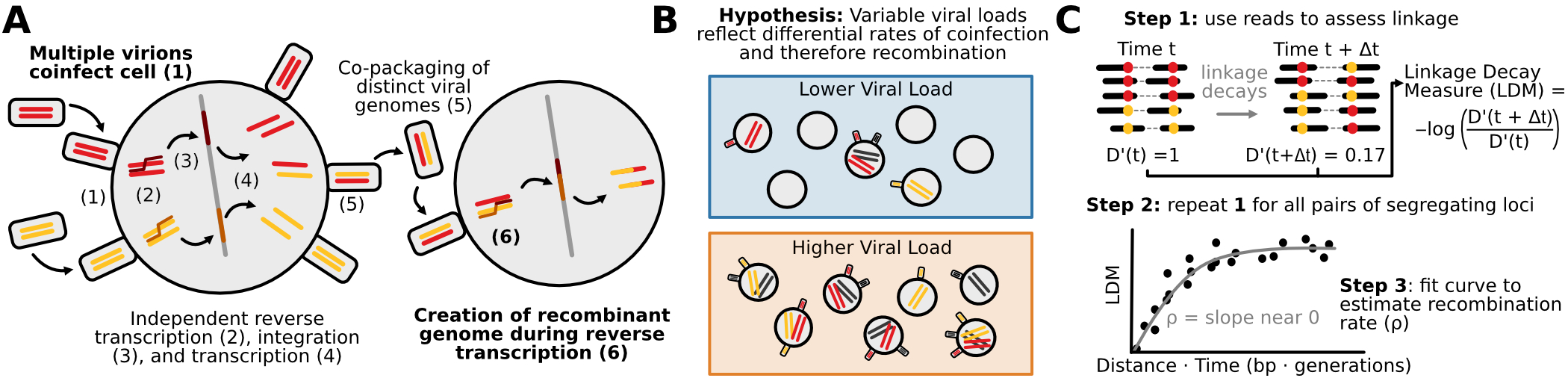

Supplement: msad260_Supplementary_Data [file msad260_supplementary_data.zip › combined_diagrams.jpg]

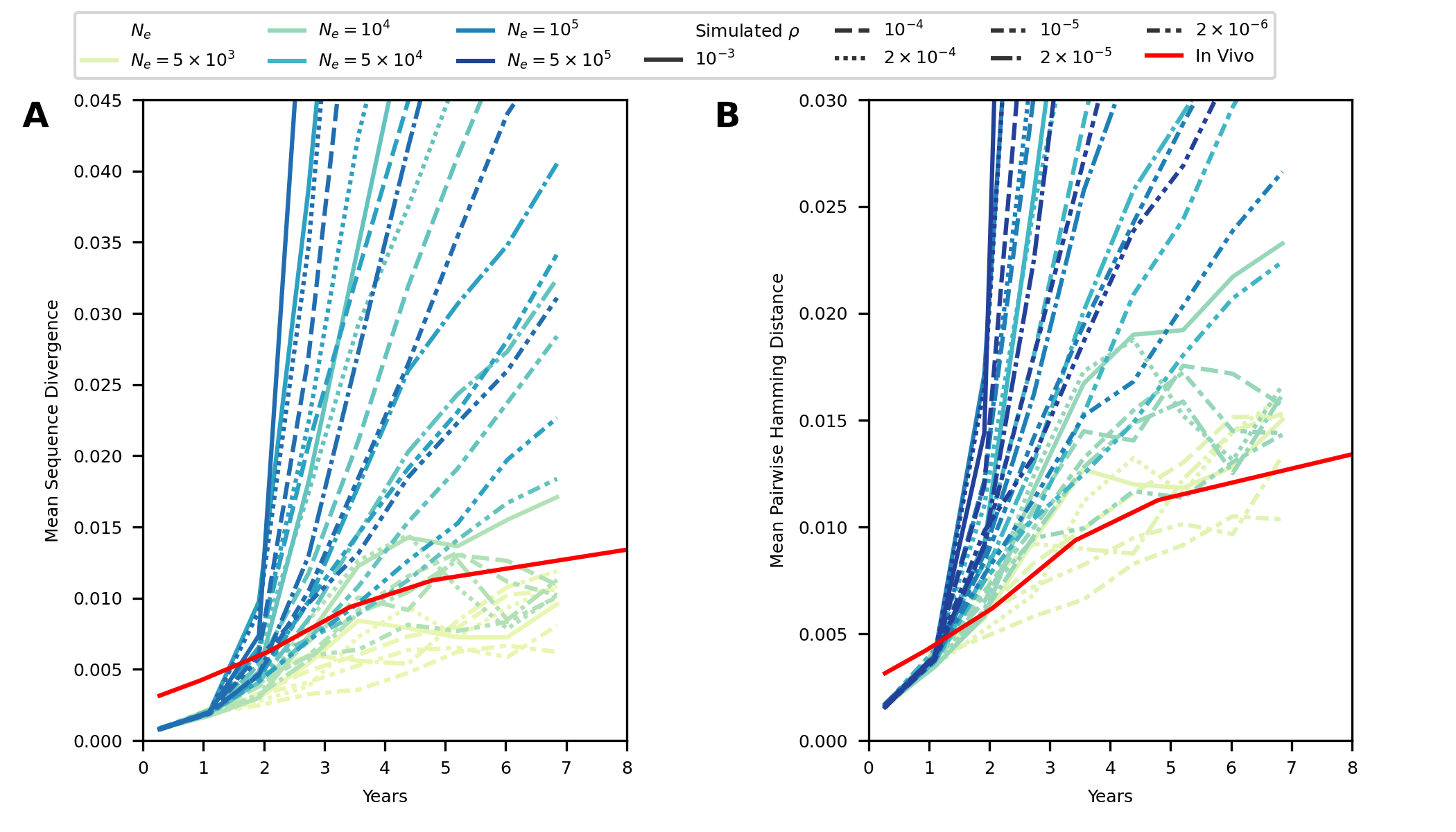

Supplement: msad260_Supplementary_Data [file msad260_supplementary_data.zip › div_div.jpg]

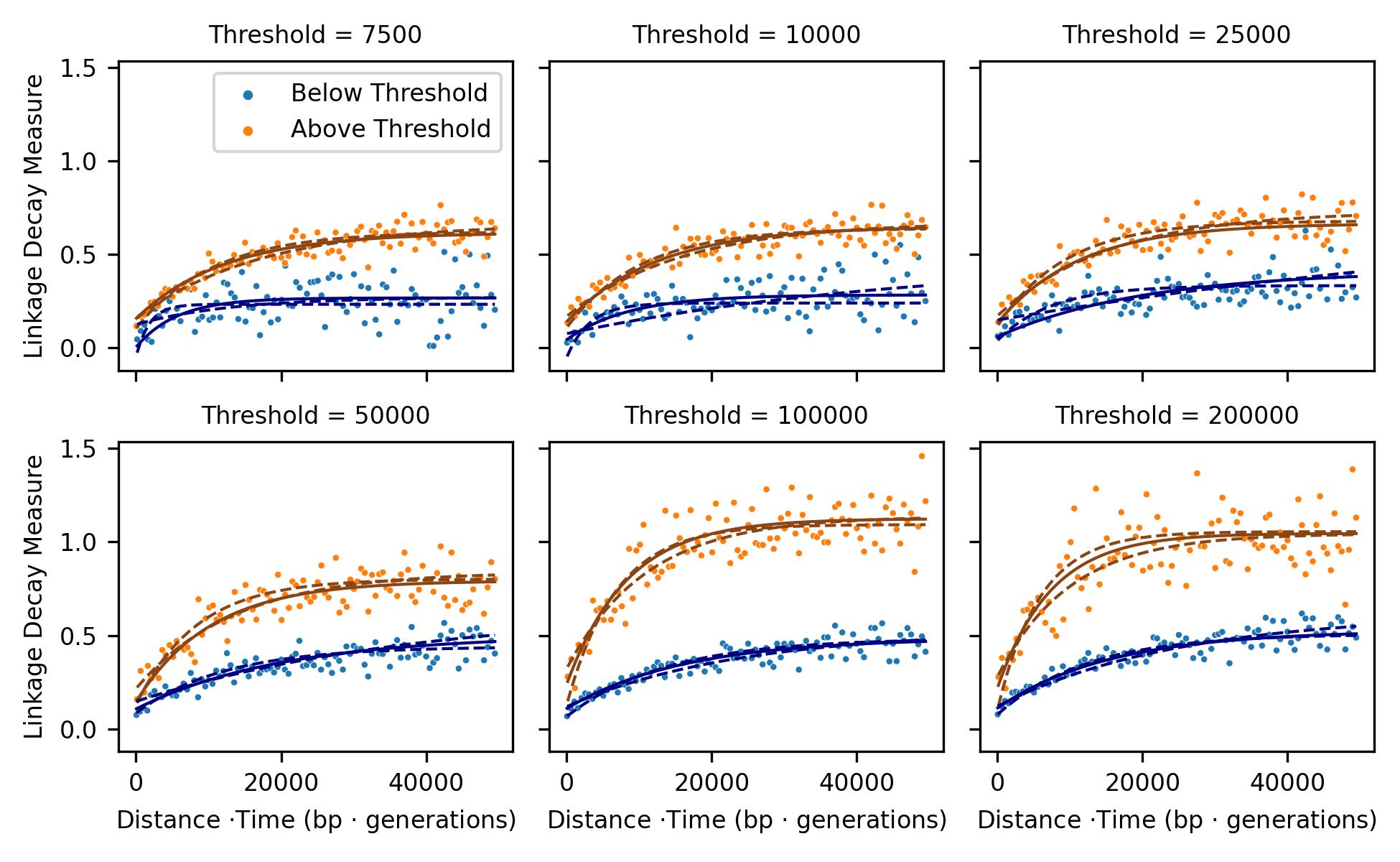

Supplement: msad260_Supplementary_Data [file msad260_supplementary_data.zip › fits_sep_groups1000.jpg]

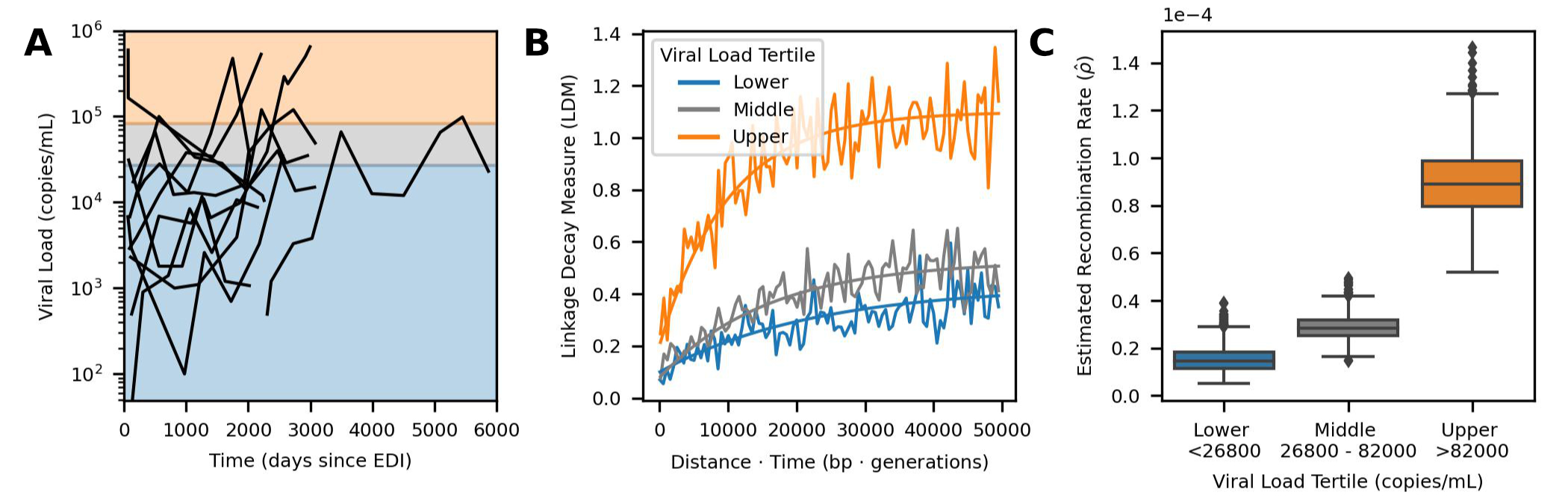

Supplement: msad260_Supplementary_Data [file msad260_supplementary_data.zip › inVivo1.jpg]

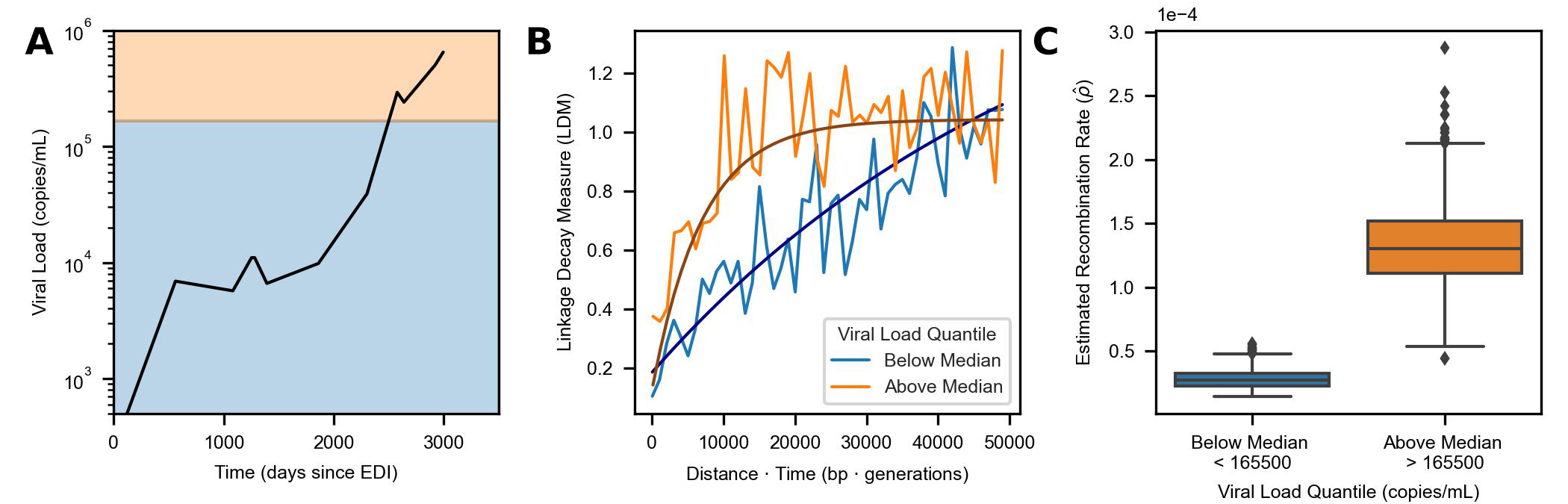

Supplement: msad260_Supplementary_Data [file msad260_supplementary_data.zip › inVivo2.jpg]
